# The Dose-Dependent Effects of Multifunctional Enkephalin Analogs on the Protein Composition of Rat Spleen Lymphocytes, Cortex, and Hippocampus; Comparison with Changes Induced by Morphine

**DOI:** 10.3390/biomedicines10081969

**Published:** 2022-08-14

**Authors:** Hana Ujcikova, Lenka Roubalova, Yeon Sun Lee, Jirina Slaninova, Jana Brejchova, Petr Svoboda

**Affiliations:** 1 Laboratory of Neurochemistry, Institute of Physiology of the Czech Academy of Sciences, Videnska 1083, 14220 Prague, Czech Republic; 2 Department of Pharmacology, University of Arizona, Tucson, AZ 85724, USA

**Keywords:** multifunctional enkephalin analogs, morphine, chronic pain treatment, rat brain, rat spleen lymphocytes, proteomic analysis, label-free quantification

## Abstract

This work aimed to test the effect of 7-day exposure of rats to multifunctional enkephalin analogs **LYS739** and **LYS744** at doses of 3 mg/kg and 10 mg/kg on the protein composition of rat spleen lymphocytes, brain cortex, and hippocampus. Alterations of proteome induced by **LYS739** and **LYS744** were compared with those elicited by morphine. The changes in rat proteome profiles were analyzed by label-free quantification (MaxLFQ). Proteomic analysis indicated that the treatment with 3 mg/kg of **LYS744** caused significant alterations in protein expression levels in spleen lymphocytes (45), rat brain cortex (31), and hippocampus (42). The identified proteins were primarily involved in RNA processing and the regulation of cytoskeletal dynamics. In spleen lymphocytes, the administration of the higher 10 mg/kg dose of both enkephalin analogs caused major, extensive modifications in protein expression levels: **LYS739** (119) and **LYS744** (182). Among these changes, the number of proteins associated with immune responses and apoptotic processes was increased. **LYS739** treatment resulted in the highest number of alterations in the rat brain cortex (152) and hippocampus (45). The altered proteins were functionally related to the regulation of transcription and cytoskeletal reorganization, which plays an essential role in neuronal plasticity. Administration with **LYS744** did not increase the number of altered proteins in the brain cortex (26) and hippocampus (26). Our findings demonstrate that the effect of κ-OR full antagonism of **LYS744** is opposite in the central nervous system and the peripheral region (spleen lymphocytes).

## 1. Introduction

The pain-relieving effects of morphine and other opioid drugs have been known for centuries. Nevertheless, the administration of high doses or prolonged treatment with morphine can cause adverse effects. Therefore, the search for new pain therapies is critically needed [1]. There are three major types of opioid receptors (ORs): μ-OR, δ-OR, and κ-OR, which serve as the primary molecular target for opioid drugs. These receptors belong to the rhodopsin family of G protein-coupled receptors (GPCRs) and are known to inhibit adenylyl cyclase (AC) activity in a pertussis toxin-dependent manner by activation of the Gi/Go class of trimeric G proteins [2,3].

This study found an unequivocal desensitization of μ- and δ-OR-stimulated G protein response in the plasma membrane-enriched fraction prepared from the cerebral cortex of rats exposed to increasing doses of morphine (10–50 mg/kg) for 10 days [4]. Furthermore, using immunoblot analysis, we have observed a specific up-regulation of AC I (8-fold) and AC II (2.5-fold) in cortical plasma membranes after 10-day morphine exposure [5]. These findings suggested that the decreased functional response of G proteins of the Gi/Go family to μ- and δ-OR agonists [4] represents the primary impulse for the subsequent compensatory increase of AC I and AC II. The expression level of AC I and AC II returned to control levels after a 20-day withdrawal period [5,6].

Chronic pain is associated with the time-dependent structural and functional reorganization of the prefrontal cortex and other brain regions, which may reflect the pain-compensatory or pain-promoting mechanisms [7]. Effective designing of innovative drug therapies remains a medical priority [8]. Multifunctional opioid ligands that interact with multiple opioid receptors have demonstrated their therapeutic potential to reduce adverse effects [9,10]. Opioid peptides are clinically limited because of their poor bioavailability, which is mainly due to poor metabolic stability and low lipophilicity, confining their ability to cross the blood-brain barrier (BBB) [11,12]. To improve their bioavailability, enkephalin-like tetrapeptides were modified by incorporating a lipophilic moiety of fentanyl to the C-terminus [11,12]. Lee et al. [10] found that modification resulted in the discovery of new multifunctional enkephalin analogs interacting with a κ-OR as well as a partial antagonist (**LYS739**) or a full antagonist (**LYS744**).

The aim of our work was to analyze the dose-dependent effects (3 mg/kg and 10 mg/kg) of **LYS739** (µ-OR/δ-OR agonist and κ-OR partial antagonist) and **LYS744** (µ-OR/δ-OR agonist and κ-OR full antagonist) on protein composition in vivo (in the rat brain cortex, hippocampus, and spleen lymphocytes), which have never been tested. Proteome changes induced by these compounds were compared with those elicited by morphine administration. Rat brain cortex and hippocampus were selected as a part of the central nervous system; rat spleen lymphocytes represented the peripheral region. Our previous data showed the opposite effect of morphine treatment and withdrawal on proteome changes in cortical and hippocampal samples [13,14,15,16]. Label-free quantification (MaxLFQ) was used for all proteomic analyses [13,14,15,17].

## 2. Materials and Methods

### 2.1. Enkephalin Analogs and Other Chemicals

Enkephalin analogs **LYS739** (Dmt-DNle-Gly-Phe(p-F)-Ppp) and **LYS744** (Dmt-DNle-Gly-Phe(p-Cl)-Ppp) were synthesized and provided by Dr. Lee from the University of Arizona [10]. Trypsin was from Promega (Madison, WI, USA).

Complete Protease Inhibitor Cocktail Tablets (1697498001) were from Roche (Basel, Switzerland). All other materials and chemicals were purchased from Sigma-Aldrich (St. Louis, MO, USA), GE Healthcare (Chicago, IL, USA) and Thermo Fischer Scientific (Waltham, MA, USA) and were of the highest purity available.

### 2.2. Morphine and Peptide Treatment of Male Wistar Rats

All animal procedures were approved by the Animal Care and Use Committee of the Institute of Physiology of the Czech Academy of Sciences IPHYS (license number 46/2019) to be in agreement with Animal Protection Law of the Czech Republic as well as the European Communities Council Directive (86/609/EEC). Male Wistar rats (weighting 150–200 g) were kept on 12/12 light/dark cycle and had ad libitum access to food and water. The first experiment was performed with groups of rats (*n* = 3) which were injected subcutaneously for 7 consecutive days with 3 mg/kg per day of morphine (group 1) or enkephalin analogs **LYS739** (group 2) and **LYS744** (group 3) dissolved in 0.9% NaCl. Control animals (group 4) (*n* = 3) received 0.9% NaCl for 7 consecutive days. The second experiment was performed in the following manner: the morphine (group 5) and enkephalin analogs **LYS739** (group 6) and **LYS744** (group 7) (*n* = 4) were injected subcutaneously for 7 consecutive days with 10 mg/kg per day of these OR ligands dissolved in 0.9% NaCl. Control animals (group 8) (*n* = 4) received 0.9% NaCl for 7 consecutive days.

### 2.3. Preparation of Samples

#### 2.3.1. Preparation of Post-Nuclear Supernatant (PNS) Fraction from Rat Brain Cortex and Hippocampus

We used the post-nuclear supernatant (PNS) fraction for all proteomic analyses according to our previous experimental experience [13,14,18]. Rats were sacrificed by decapitation under ether narcosis, cortices and hippocampi dissected, washed from remaining blood by ice-cold saline, frozen in liquid nitrogen, and stored at −80 °C until use. Pooled tissue pieces (2 g w.w. per 10 mL) were diluted in STEM medium (250 mM sucrose, 20 mM Tris-HCl, 3 mM MgCl2, 1 mM EDTA, pH 7.6) containing complete protease inhibitor cocktail and fresh 1 mM phenylmethylsulfonyl fluoride (PMSF), homogenized in Teflon-glass homogenizer for 7 min at 1800 rpm, and centrifuged for 7 min at 1200× *g*. The PNS fractions were snap frozen in liquid nitrogen and stored at −80 °C until use.

#### 2.3.2. Isolation of Lymphocytes from Spleen; Preparation of Post-Nuclear Supernatant (PNS) Fraction

Isolation of lymphocytes from spleens was performed as described by Cechova et al. [19]. Briefly, spleens were dissected, and the single-cell suspension was prepared in RPMI-1640 medium (Sigma-Aldrich, St. Louis, MO, USA) supplemented with 5% (*v*/*v*) heat-inactivated Fetal Bovine Serum (FBS) GibcoTM (Thermo Fischer Scientific, Waltham, MA, USA). Subsequently, the single-cell suspension was layered on the top of Ficoll-Paque Plus (GE Healthcare, Chicago, IL, USA) and centrifuged for 35 min at 800× *g* at 19 °C. The lymphocyte fraction was washed twice in RPMI-1640 medium containing 5% FBS and twice in PBS. Finally, lymphocytes were resuspended in 0.5 mL of PBS containing complete protease inhibitor cocktail, snap frozen in liquid nitrogen, and stored at −80 °C until use.

When preparing PNS fraction, suspension of lymphocytes was diluted in 50 mM Tris-HCl (pH 7.6), 3 mM MgCl2, 1 mM EDTA (TME buffer) containing fresh 1 mM PMSF and complete protease inhibitor cocktail, and homogenized in tight Teflon-glass homogenizer for 7 min at 1400 rpm on ice. The cell homogenates were centrifuged for 7 min at 500× *g*. The PNS fractions were snap frozen in liquid nitrogen and stored at −80 °C until use.

### 2.4. Label-Free Quantification (LFQ)

LFQ data were evaluated in two rounds (the effect of 3 mg/kg—round 1, the effect of 10 mg/kg—round 2) for 12 samples in three technical replicates of each round: lymphocytes samples (LMOR 1-3, LLYS739 1-3, LLYS744 1-3, LCTR 1-3), cortical samples (CMOR 1-3, CLYS739 1-3, CLYS744 1-3, CCTR 1-3) and hippocampal samples (HMOR 1-3, HLYS739 1-3, HLYS744 1-3, HCTR 1-3). Sample preparation was performed as described before in Ujcikova et al. [13,14]. Eluting peptide cations converted to gas-phase ions were analyzed on Thermo Orbitrap Fusion (Thermo ScientificTM) mass spectrometer. Raw data were analyzed and quantified by using the MaxQuant software. The false discovery rate (FDR) was set to 1% for proteins and peptides (the minimum of peptide length was 7 amino acids). The MS/MS spectra search against the Uniprot *Rattus norvegicus* database of protein sequences (https://www.uniprot.org, accessed on 3 November 2021) was done by using the Andromeda search engine (enzyme specificity: C-terminal to arginine and lysine; fixed modification: carbamidomethylation of cysteine; variable modifications: N-terminal protein acetylation, methionine oxidation). Quantifications with the label-free algorithm were performed in MaxQuant according to Cox et al. [17], data were analyzed using Perseus 1.6.15.0 software. Binary logarithms of intensity ratios were then median calculated for each group, and the difference between control and treated samples was determined. Only at least 2-fold significant differences calculated for at least two measured values from 3 replicates were taken into consideration. *p*-values (*p* ≤ 0.05) were calculated via GraphPad Prism 8.3.0 software (San Diego, CA, USA).

### 2.5. Protein Determination

Lowry method [20] was used for determination of protein concentration in all samples: LMOR, LLYS739, LLYS744, LCTR, CMOR, CLYS739, CLYS744, CCTR, HMOR, HLYS739, HLYS744, and HCTR. Bovine serum albumin (Fraction V) was used as a standard (Sigma-Aldrich, St. Louis, MO, USA).

## 3. Results and Discussion

### 3.1. LFQ Analysis of Rat Spleen Lymphocytes after 7-Day Treatment with Morphine, **LYS739**, and **LYS744**: The Dose-Effect of 3 mg/kg

**Figure 1:**
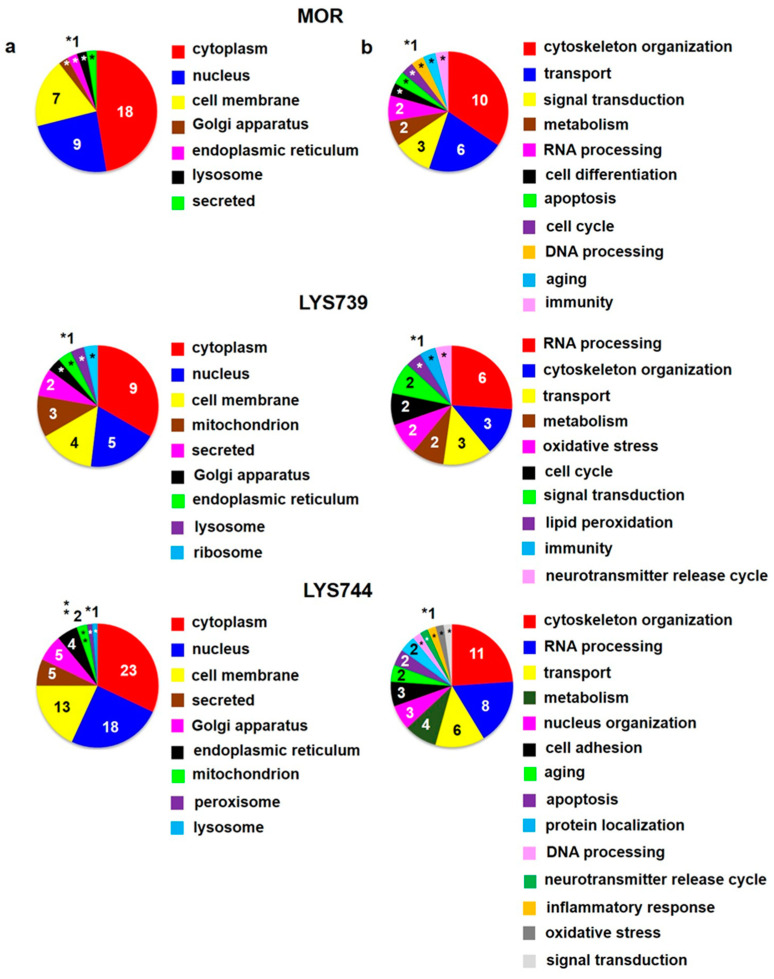
Subcellular localization (**a**) and function (**b**) of significantly altered proteins (at least 2-fold) identified in rat spleen lymphocytes after 7-day treatment with 3 mg/kg of **morphine** (**MOR**, **upper** panels), **LYS739** (**middle** panels), and **LYS744** (**lower** panels); according to the current annotations in the Uniprot database (https://www.uniprot.org, accessed on 28 January 2022). The numbers represent proteins found within each subcellular localization and functional category, *1, **2.

The study had some limitations. The first one is that we had to work with small amounts of peptides **LYS739** and **LYS744**. For that reason, each testing group included only three of four animals. Second, this study was not designed to search for differences between male and female rats and only included male rats as in our previous proteomic analyses [13,14,15,18]. The third one is connected with mass spectrometry-based data. Some measurements may result in technical failures. Therefore, three technical replicates of each sample enabled us to perform statistical analysis. Label-free quantification (LFQ) analysis revealed 27 altered proteins in rat spleen lymphocytes after 7-day morphine treatment (3 mg/kg). The minimum of 2-fold expression differences were calculated for at least 2 measured values from 3 technical replicates, Table S1a. The 12 proteins were upregulated, and 15 proteins were downregulated. Their subcellular localization and functional significance unveiled most proteins of cytoplasmic origin related to the cytoskeletal changes, Figure 1a,b (upper panels). These proteins were associated with actin filament bundle assembly: beta-adducin (*Add2*, ↑4.0-fold), spectrin beta chain (*Sptbn2*, ↑3.9-fold); microtubule polymerization: tubulin polymerization-promoting protein family member 3 (Tppp3, ↑2.6-fold), tubulin beta-3 chain (*Tubb3*, ↓5.1-fold), or intermediate filament organization: vimentin (*Vim*, ↓2.8-fold), Table S1a.

Proteomic analysis indicated that 7-day treatment with 3 mg/kg of **LYS739** altered the expression level of 20 proteins more than twice, 5 were upregulated and 15 downregulated, Table S1a. Among these, 6 proteins were involved in RNA processing: H/ACA ribonucleoprotein complex subunit 4 (*Dkc1*, ↑2.2-fold), hepatoma-derived growth factor-related protein 2 (*Hdgfrp2*, ↑2.0-fold), treacle ribosome biogenesis factor 1 (*Tcof1*, ↓3.0-fold), 60S acidic ribosomal protein P2 (*Rplp2*, ↓2.6-fold), serine/arginine repetitive matrix 2 (*Srrm2*, ↓2.6-fold), and CREB-regulated transcription coactivator 2 (*Crtc2*, ↓2.0-fold). Modulation of transcription factors (TF), such as cyclic AMP response element binding protein (CREB) may represent a potential mechanism for persisted opioid-induced plasticity in the brain [21]. According to Algmandi and Alshehri [22], morphine administration was accompanied by a reduced mRNA expression level of CREB in the nucleus accumbens. Other functional categories associated with the identified proteins were organization of the cytoskeleton (3), transport (3), metabolism (2), oxidative stress (2), cell cycle (2), signal transduction (2), lipid peroxidation (1), immunity (1), and neurotransmitter release cycle (1), Figure 1a,b (middle panels).

**Figure 2:**
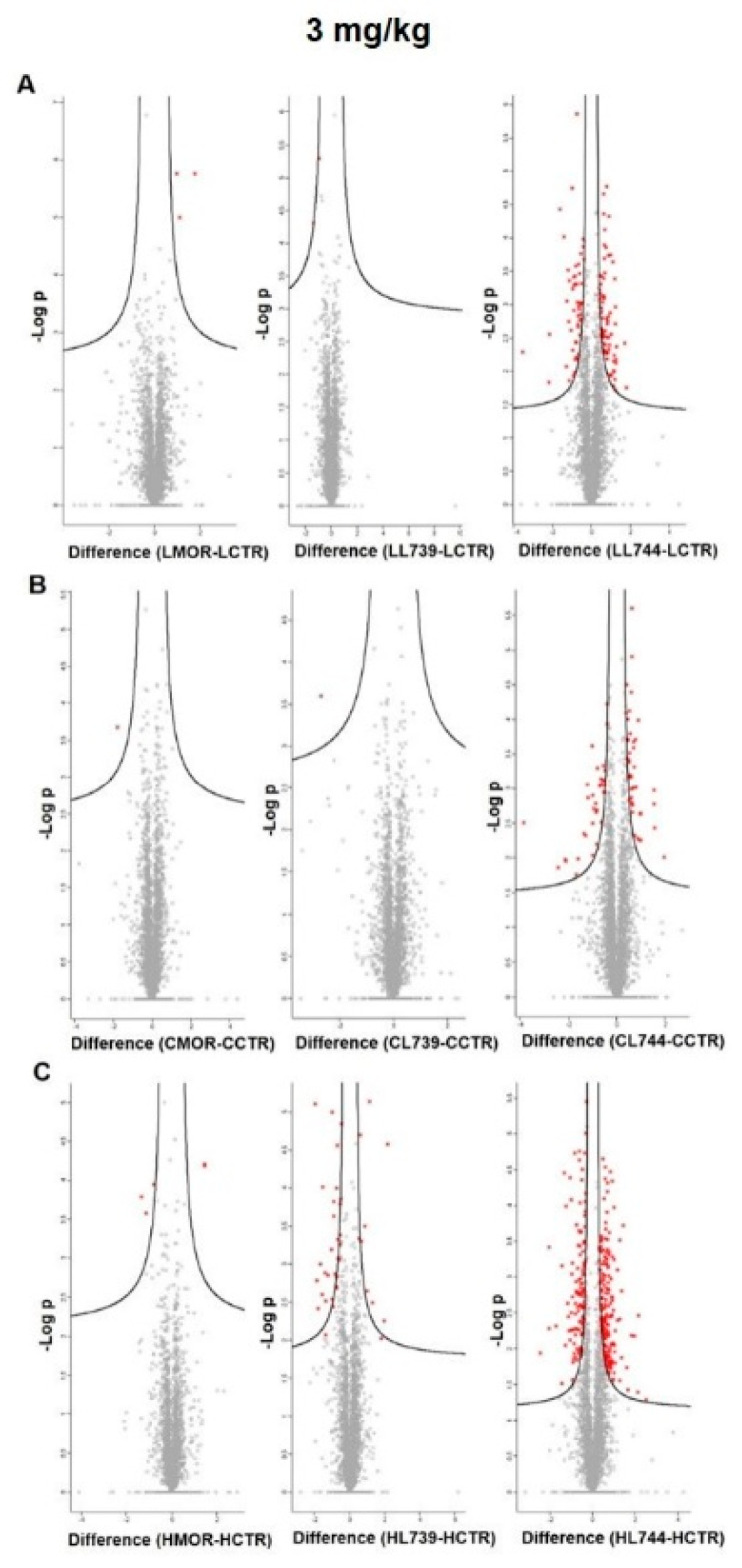
Volcano plots indicating significantly altered proteins identified in rat spleen lymphocytes (**A**), brain cortex (**B**), and hippocampus (**C**) after 7-day treatment with 3 mg/kg of **morphine** (**MOR**, **left** panels), **LYS739** (**middle** panels), and **LYS744** (**right** panels). Significantly altered proteins are labeled in red (the star symbols); performed with Perseus software version 1.6.15.0. L739, **LYS739**; L744, **LYS744**.

**Figure 3:**
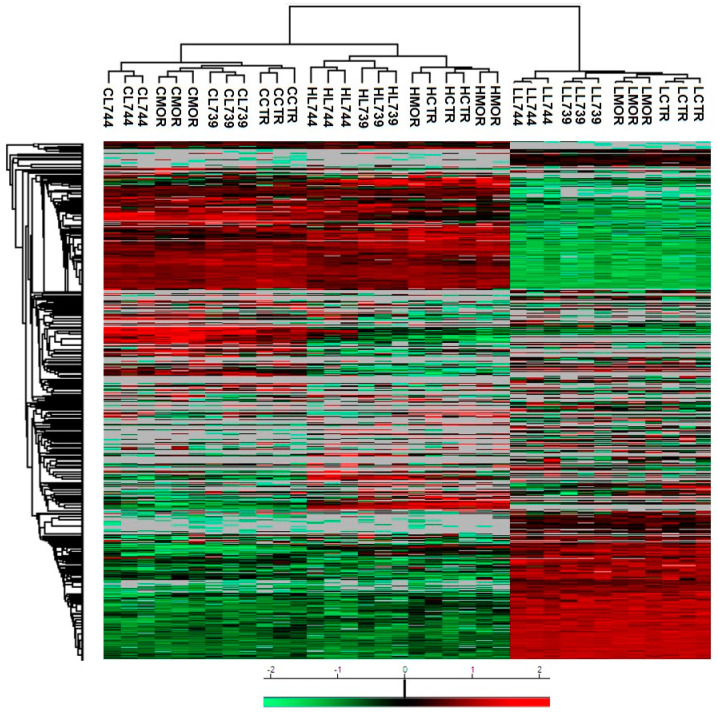
Heatmap showing hierarchical clustering performed on LFQ intensity values (with Perseus software version 1.6.15.0) of all 4717 proteins identified in rat spleen lymphocytes (L), brain cortex (C), and hippocampus (H) after 7-day treatment with 3 mg/kg of **morphine** (**MOR**), **LYS739**, and **LYS744**. Significantly upregulated proteins are labeled in red, significantly downregulated proteins are labeled in green. L739, **LYS739**; L744, **LYS744**.

**LYS744** had the most significant effect on the change of protein expression levels in rat spleen lymphocytes. LFQ analysis detected 45 altered proteins: 29 proteins upregulated more than 2-fold and 16 proteins downregulated, Table S1a. More than half of these were related to actin cytoskeleton organization (11), RNA processing (8), and transport (6), Figure 1a,b (lower panels). DNA helicase (*Chd4*, ↑3.8-fold) was found to be the most significantly upregulated, while globin c2 (*Hba-a2*, ↓11.6-fold) the most decreased. DNA helicases play key roles in DNA replication, transcription, DNA repair, and many other processes to maintain genomic integrity and cellular homeostasis [23]. Helicase variants are frequently upregulated in cancerous tissues [24]. Volcano plots representing significantly altered proteins identified in rat spleen lymphocytes after treatment with 3 mg/kg of morphine, **LYS739**, and **LYS744** are depicted in Figure 2A. Hierarchical heatmap clustering of all identified protein expression profiles in rat spleen lymphocytes is presented in Figure 3.

### 3.2. LFQ Analysis of Rat Brain Cortex after 7-Day Treatment with Morphine, **LYS739**, and **LYS744**: The Dose-Effect of 3 mg/kg

**Figure 4:**
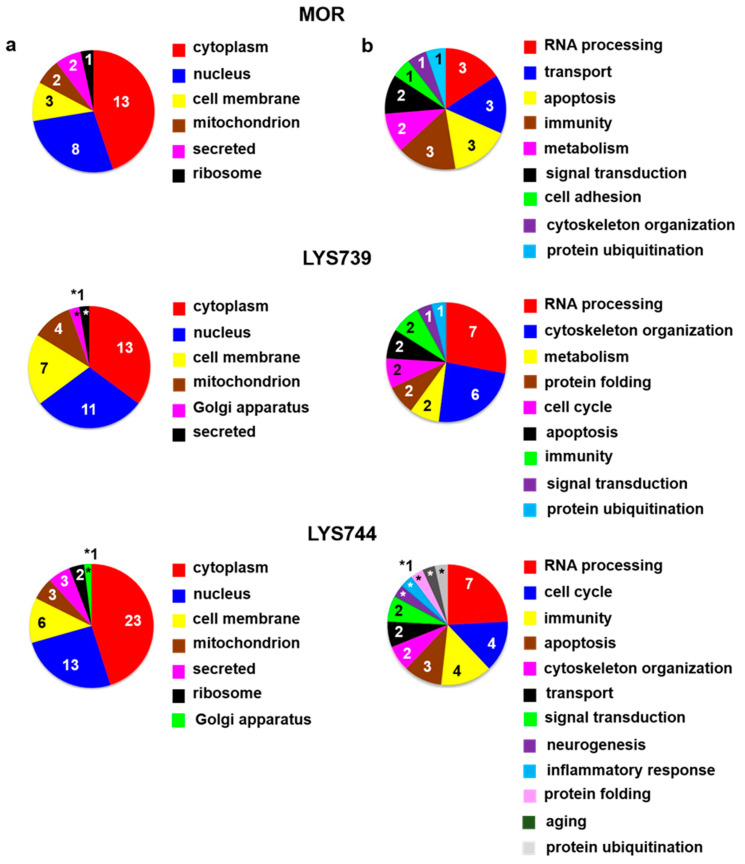
Subcellular localization (**a**) and function (**b**) of significantly altered proteins (at least 2-fold) identified in rat brain cortex after 7-day treatment with 3 mg/kg of **morphine** (**MOR**, **upper** panels), **LYS739** (**middle** panels), and **LYS744** (**lower** panels); according to the current annotations in the Uniprot database (https://www.uniprot.org, accessed on 28 January 2022). The numbers represent proteins found within each subcellular localization and functional category, *1.

The effect of 7-day morphine treatment (3 mg/kg) on protein alterations in the rat brain cortex was slightly less than that detected in the proteome profile of spleen lymphocytes. In cortical samples, LFQ analysis detected 5 proteins significantly upregulated and 10 proteins were downregulated, Table S1b. From the functional point of view, 3 proteins were involved in RNA processing: U1 small nuclear ribonucleoprotein 70 kDa (*Snrnp70*, ↓3.5-fold), DAZ-associated protein 1 (*Dazap1*, ↓2.3-fold), nucleolar RNA helicase 2 (*Ddx21*, ↓2.2-fold), 3 proteins participated in oxygen and protein transport: globin c2 (*Hba-a2*, ↓13.6-fold), exportin 5 (*Xpo5*, ↓2.4-fold), alpha globin (*Hba-a3*, ↓2.2-fold), and 3 proteins were related to apoptosis: ribosomal protein S6 kinase (*Rps6ka3*, ↑2.7-fold), protein S100-A9 (*S100a9*, ↓3.7-fold), programmed cell death protein 4 (*Pdcd4*, ↓2.3-fold). *S100a9* is a member of the S100 family proteins representing essential factors in inflammation and apoptosis-inducing activities [25]. Decreased levels of *S100a9* and *Pdcd4* may suggest the activation of compensatory defense mechanisms, as chronic morphine treatment was related to a strong upregulation of the pro-apoptotic Fas receptor and a moderate downregulation of the anti-apoptotic Bcl-2 [26]. The other functional categories were associated with immunity (3), metabolism (2), signal transduction (2), cell adhesion (1), cytoskeleton organization (1), and protein ubiquitination (1), Figure 4a,b (upper panels).

**LYS739** treatment resulted in 20 altered proteins (7 upregulated, 13 downregulated), mainly involved in RNA processing: polypyrimidine tract-binding protein 1 (*Ptbp1*, ↓8.4-fold), U1 small nuclear ribonucleoprotein 70 kDa (*Snrnp70*, ↓6.5-fold), heterogeneous nuclear ribonucleoprotein U-like 2 (*Hnrnpul2*, ↓5.4-fold), probable ATP-dependent RNA helicase DDX46 (*Ddx46*, ↓4.2-fold), nucleolar RNA helicase 2 (*Ddx21*, ↓3.2-fold), splicing factor 3b, subunit 1 (*Sf3b1*, ↓2.2-fold), and small nuclear ribonucleoprotein Sm D2 (*Snrpd2*, ↓2.0-fold). Six cytoskeletal proteins were found to differ significantly: filamin A (*Flna*, ↑3.5-fold), prefoldin 1 (*Pfdn1*, ↑2.4-fold), formin-like 1 (*Fmnl1*, ↑2.3-fold), protein S100-A9 (*S100a9*, ↓10.5-fold), IQ motif-containing GTPase-activating protein 1 (*Iqgap1*, ↓5.2-fold), and CD2-associated protein (*Cd2ap*, ↓4.7-fold), Table S1b, Figure 4a,b (middle panels).

As in the case of spleen lymphocytes, treatment with **LYS744** identified the highest number of alterations in the brain cortex. Among 31 proteins, 11 were upregulated, and 20 proteins had the decreased expression levels, Table S1b. LFQ analysis identified proteins related to the regulation of RNA processes (7), regulation of cell cycle (4), immunity (4), apoptotic processes (3), reorganization of the cytoskeleton (2), protein transport (2), signal transduction (2), brain development, (1) or protein folding (1), Figure 4a,b (lower panels). *Flna* (↑3.9-fold), *Pfdn1* (↑2.6-fold), *Ptbp1* (↓14.4-fold), *S100a9* (↓5.4-fold), *Snrnp70* (↓4.4-fold), *Cd2ap* (↓3.2-fold), *Hba-a2* (↓2.6-fold), *Dazap1* (↓2.4-fold), and *Ddx21* (↓2.0-fold) were found dysregulated after morphine administration and **LYS739** treatment as well, see above. Volcano plots representing significantly altered proteins identified in rat brain cortex after treatment with 3 mg/kg of morphine, **LYS739**, and **LYS744** are depicted in Figure 2B. Hierarchical heatmap clustering of all identified protein expression profiles in the rat brain cortex is presented in Figure 3.

### 3.3. LFQ Analysis of Rat Hippocampus after 7-Day Treatment with Morphine, **LYS739**, and **LYS744**: The Dose-Effect of 3 mg/kg

**Figure 5:**
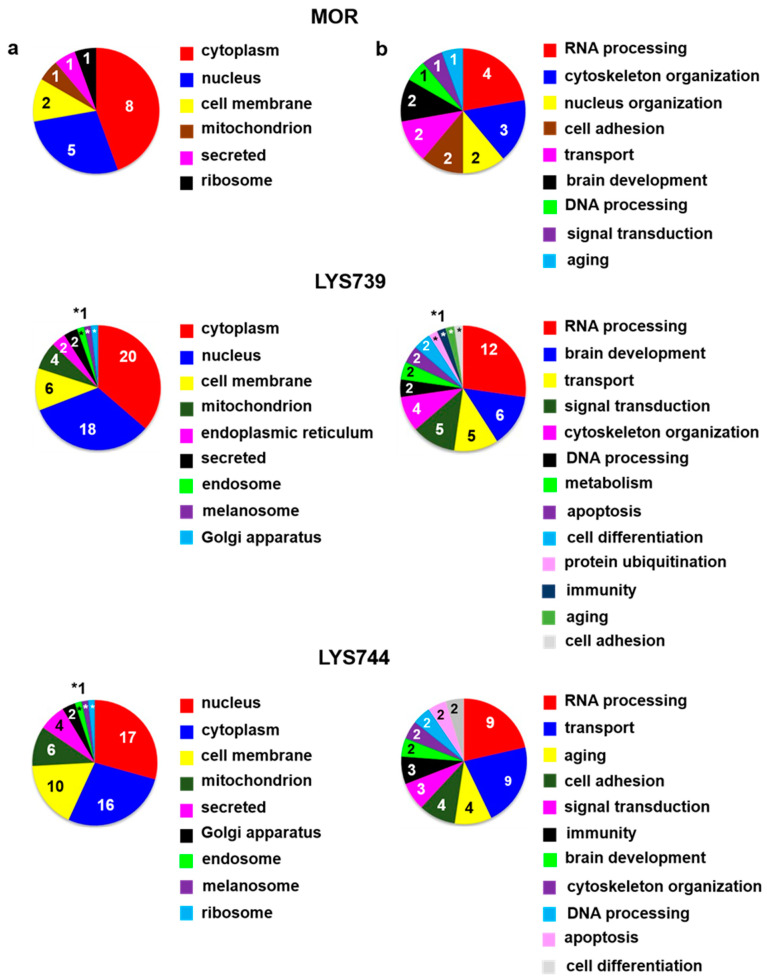
Subcellular localization (**a**) and function (**b**) of significantly altered proteins (at least 2-fold) identified in rat hippocampus after 7-day treatment with 3 mg/kg of **morphine** (**MOR**, **upper** panels), **LYS739** (**middle** panels), and **LYS744** (**lower** panels); according to the current annotations in the Uniprot database (https://www.uniprot.org, accessed on 28 January 2022). The numbers represent proteins found within each subcellular localization and functional category, *1.

LFQ analysis indicated 14 proteins with changed expression levels at least 2-fold after 7-day treatment with morphine. This amount is similar to the number of alterations in the rat brain cortex (15) but lower twice in comparison with spleen lymphocytes (27). Eight proteins were upregulated, 6 had decreased levels, Table S1c. According to the current annotations in the Uniprot database, these proteins were mainly associated with the regulation of transcription and translation: methyl-CpG-binding protein 2 (*Mecp2*, ↑2.6-fold), general transcription factor II-I (*Gtf2i*, ↑2.2-fold), CXXC-type zinc finger protein 1 (*Cxxc1*, ↑2.2-fold), 60S ribosomal protein L29 (*Rpl29*, ↑2.0-fold), and cytoskeletal changes: vasodilator-stimulated phosphoprotein (*Vasp*, ↑4.1-fold), transgelin (*Tagln*, ↑2.7-fold), alpha-internexin (*Ina*, ↓2.2-fold), Figure 5a,b (upper panels). Interestingly, in our previous study, the level of *Ina* was detected as hypophosphorylated (↓2.3-fold) in rat hippocampal samples after 3-month morphine withdrawal [15].

The effect of **LYS739** on rat hippocampal protein profile was higher than that of morphine treatment; proteomic analysis detected 36 altered proteins (8 upregulated, 28 downregulated), as shown in Table S1c. Among different biological processes, most proteins were involved in RNA processing (12), brain development (6), transport (5), and signal transduction (5), Figure 5a,b (middle panels). Notably, all proteins involved in brain development were decreased: cell cycle exit and neuronal differentiation protein 1 (*Cend1*, ↓3.9-fold), neurofilament heavy polypeptide (*Nefh*, ↓3.7-fold), metallothionein-3 (*Mt3*, ↓3.5-fold), *Ina* (↓2.0-fold), plasma membrane calcium-transporting ATPase 4 (*Atp2b4*, ↓2.0-fold), myristoylated alanine-rich C-kinase substrate (*Marcks*, ↓2.0-fold). Opioid drugs were reported to change hippocampal plasticity and inhibit adult neurogenesis [27]. Contrarily, opioid withdrawal was connected with increased neuronal plasticity [28,29].

Treatment with **LYS744** revealed 42 alterations in protein expression levels, which is more than in the brain cortex (31) and similar to spleen lymphocytes (45). Twenty-four proteins were upregulated and 18 proteins were downregulated, Table S1c. RNA processing (9) and transport (9) were the main functional categories, Figure 5a,b (lower panels). *Mecp2* (↑2.6-fold) and *Rpl29* (↑2.0-fold) were found significantly upregulated after morphine treatment as well, see above. Among upregulated proteins, four of these were associated with the change in energy metabolism: electron transfer flavoprotein-ubiquinone oxidoreductase, mitochondrial (*Etfdh*, ↑2.6-fold), ATPase inhibitor, mitochondrial (*Atpif*,↑2.4-fold), complex I-B17 (*Ndufb6*, ↑2.4-fold), and cytochrome c oxidase subunit 2 (*Mtco2*, ↑2.3-fold). We previously reported that the majority of altered hippocampal proteins were related to energy metabolism after both chronic morphine treatment (10–50 mg/kg, 10 days) and subsequent drug withdrawal (3 weeks, 3 months, 6 months) [14,15,16]. Volcano plots representing significantly altered proteins identified in rat hippocampus after treatment with 3 mg/kg of morphine, **LYS739**, and **LYS744** are depicted in Figure 2C. Hierarchical heatmap clustering of all identified protein expression profiles in rat hippocampus is presented in Figure 3.

### 3.4. LFQ Analysis of Rat Spleen Lymphocytes after 7-Day Treatment with Morphine, **LYS739**, and **LYS744**: The Dose-Effect of 10 mg/kg

**Figure 6:**
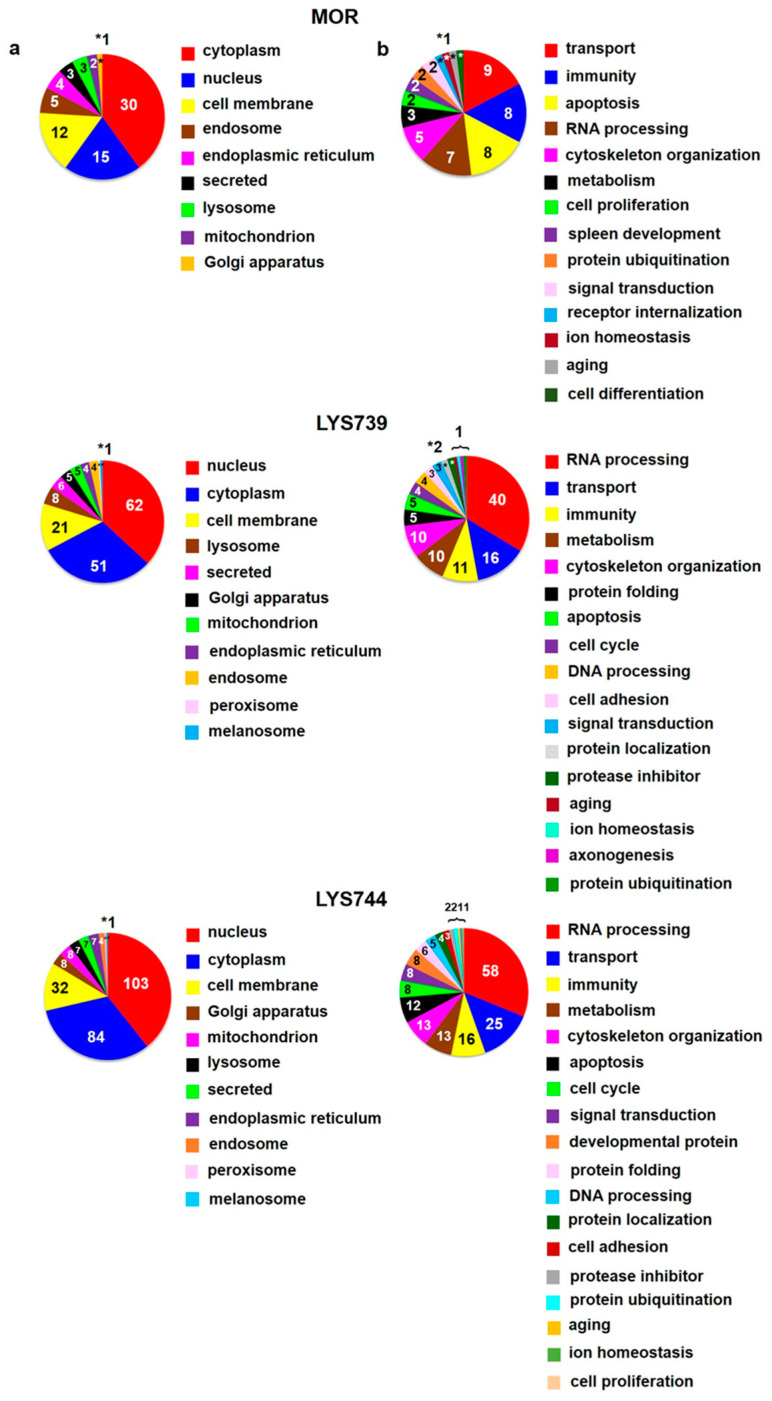
Subcellular localization (**a**) and function (**b**) of significantly altered proteins (at least 2-fold) identified in rat spleen lymphocytes after 7-day treatment with 10 mg/kg of **morphine** (**MOR**, **upper** panels), **LYS739** (**middle** panels), and **LYS744** (**lower** panels); according to the current annotations in the Uniprot database (https://www.uniprot.org, accessed on 22 April 2022). The numbers represent proteins found within each subcellular localization and functional category, *1 or *2.

In the second round of our experiments, we increased the dose of morphine and enkephalin analogs from 3 mg/kg to 10 mg/kg. We applied this amount of OR drugs to rats for 7 consecutive days to test the effect of increased doses on protein changes in spleen lymphocytes, cortex, and hippocampus. This scheme was modified according to our previously established protocols [4,5,13,14,18]. LFQ analysis revealed 45 altered proteins (at least 2-fold) in spleen lymphocytes after morphine treatment. Upregulation was found for 29 proteins; 16 proteins were downregulated, Table S2a. According to the current annotations in the Uniprot database, their molecular functions and biological processes were mostly involved in transport (9), immunity (8), apoptosis (8), and RNA processing (7), Figure 6a,b (upper panels). When compared with the effect of a lower dose, the number of proteins associated with immune responses and apoptotic processes was increased, suggesting the state of cell dysfunction and neuroinflammation after chronic morphine administration. Among upregulated apoptotic proteins were interferon-induced protein with tetratricopeptide repeats 3 (*Ifit3*, ↑4.9-fold), sialic acid-binding Ig-like lectin 1 (*Siglec1*, ↑3.8-fold), interferon-induced protein with tetratricopeptide repeats 2 (*Ifit2*, ↑3.4-fold), MAP kinase-activating death domain protein (*Madd*, ↑2.8-fold), galectin-5 (*Lgals5*, ↑2.3-fold), and interferon activated gene 204 (*Mnda*, ↑2.2-fold).

**LYS739** treatment resulted in the change of 119 proteins (51 proteins were upregulated, 68 downregulated). The number of altered proteins was six times higher than after the dose of 3 mg/kg, Table S2a. The majority of identified proteins were related to RNA processing (40), transport (16), immunity (11), metabolism (10), and cytoskeletal changes (10), Figure 6a,b (middle panels). Seven proteins that participated in immune responses were upregulated: Ig gamma-2C chain C region (*N/A*, ↑27.5-fold), proto-oncogene vav (*Vav1*, ↑9.5-fold), tyrosine-protein kinase (*Btk*, ↑3.4-fold), *Mnda* (↑2.8-fold), macrophage-expressed gene 1 protein (*Mpeg1*, ↑2.4-fold), tap-binding protein (*Tapbp*, ↑2.3-fold), TNF receptor-associated factor 6 (*Traf6*, ↑2.2-fold).

**Figure 7:**
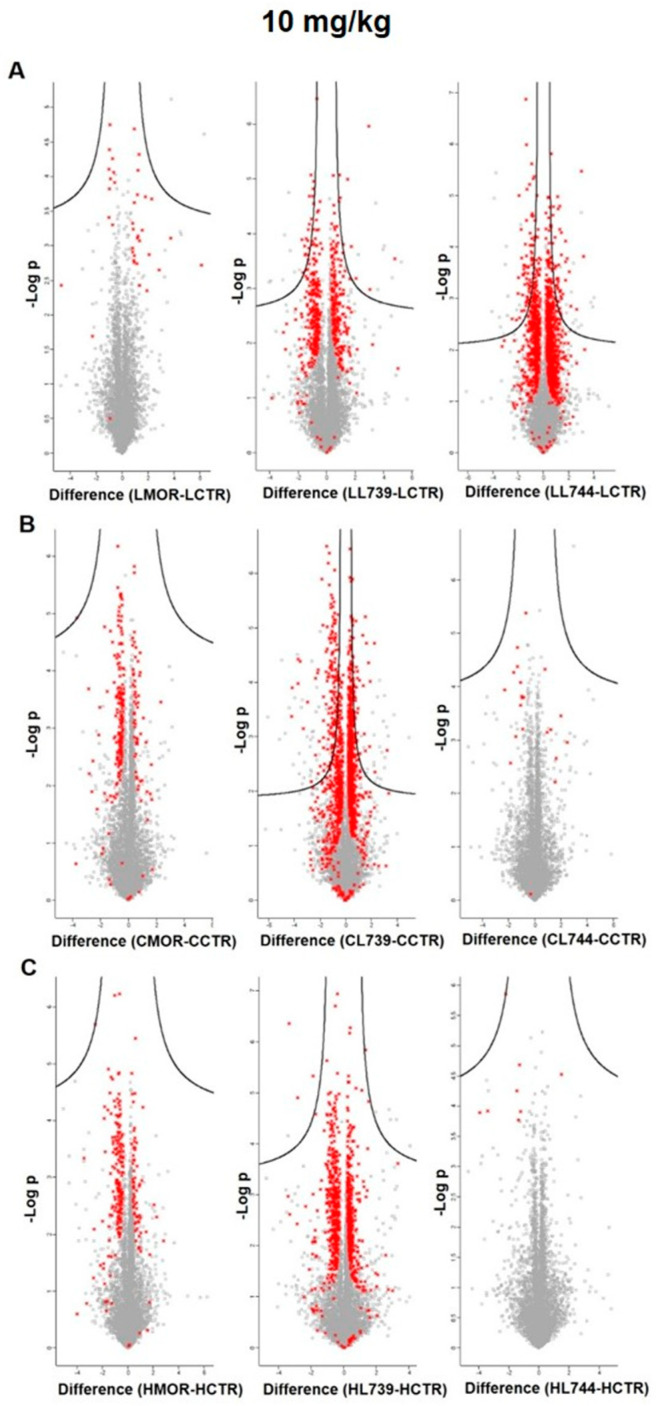
Volcano plots indicating significantly altered proteins identified in rat spleen lymphocytes (**A**), brain cortex (**B**), and hippocampus (**C**) after 7-day treatment with 10 mg/kg of **morphine** (**MOR**, **left** panels), **LYS739** (**middle** panels), and **LYS744** (**right** panels). Significantly altered proteins are labeled in red (the star symbols); performed with Perseus software version 1.6.15.0. L739, **LYS739**; L744, **LYS744**.

**Figure 8:**
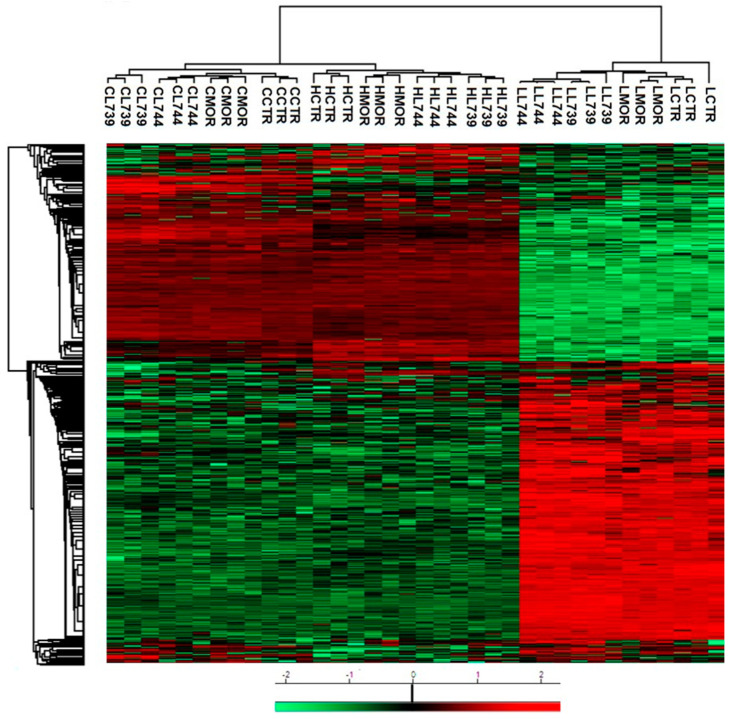
Heatmap showing hierarchical clustering performed on LFQ intensity values (with Perseus software version 1.6.15.0) of all 6228 proteins identified in rat spleen lymphocytes (L), brain cortex (C), and hippocampus (H) after 7-day treatment with 10 mg/kg of **morphine** (**MOR**), **LYS739**, and **LYS744**. Significantly upregulated proteins are labeled in red, significantly downregulated proteins are labeled in green. L739, **LYS739**; L744, **LYS744**.

The treatment with **LYS744** caused the most significant differences in protein expression levels of spleen lymphocytes. Four times more proteins were altered (182 proteins, 88 upregulated, 94 downregulated) in comparison with the lower dose of **LYS744**, Table S2a. Identified proteins were primarily associated with RNA processing (58), transport (25), immune responses (16), metabolic changes (13), cytoskeletal changes (13), and apoptotic processes (12), Figure 6 a,b (lower panels). *Vav1* (↑3.7-fold), *Mnda* (↑3.4-fold), *Mpeg1* (↑2.6-fold), and *Btk* (↑2.5-fold) were identified in samples treated with LYS739 as well, see above. Both immunosuppressive and immuno-stimulating effects have been described after opioid administration [30,31,32]. Recent studies have shown that *Vav* proteins are essential for the homeostasis of the central nervous, cardiovascular, and immune systems. They are considered potential therapeutic targets for several pathological conditions [33,34]. Volcano plots representing significantly altered proteins identified in rat spleen lymphocytes after treatment with 10 mg/kg of morphine, **LYS739**, and **LYS744** are depicted in Figure 7A. Hierarchical heatmap clustering of all identified protein expression profiles in rat spleen lymphocytes is presented in Figure 8.

### 3.5. LFQ Analysis of Rat Brain Cortex after 7-Day Treatment with Morphine, **LYS739**, and **LYS744**: The Dose-Effect of 10 mg/kg

**Figure 9:**
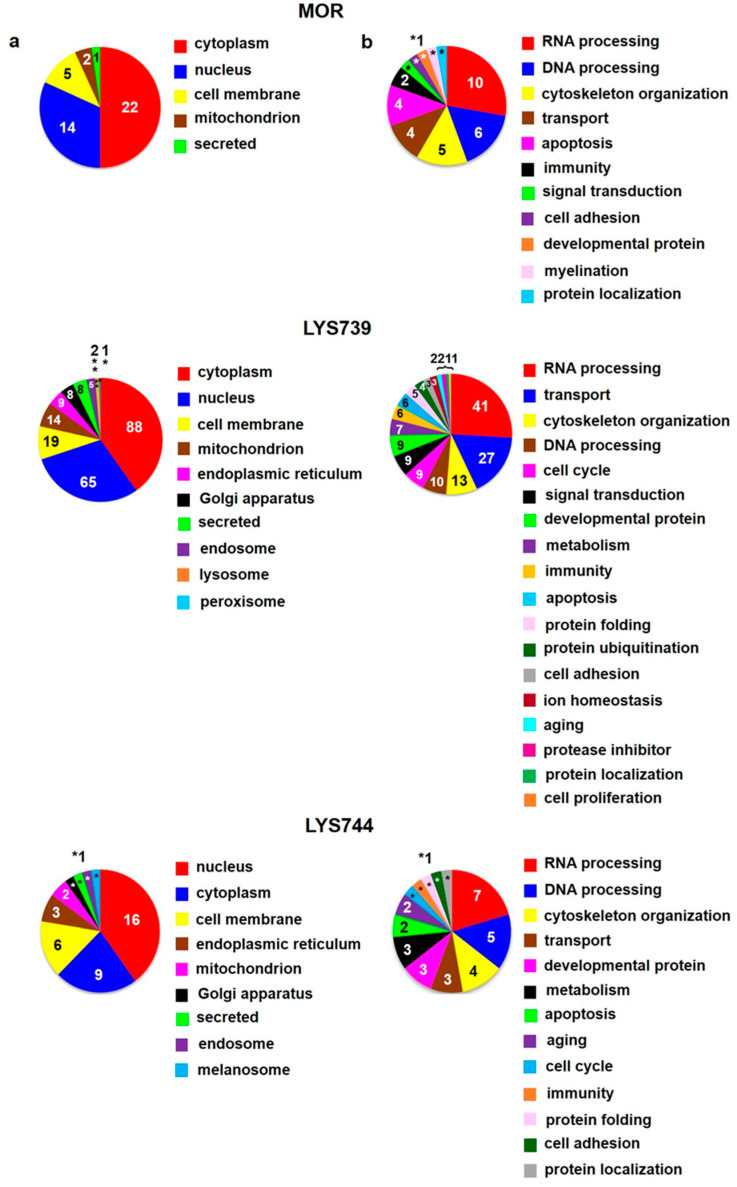
Subcellular localization (**a**) and function (**b**) of significantly altered proteins (at least 2-fold) identified in rat brain cortex after 7-day treatment with 10 mg/kg of **morphine** (**MOR**, **upper** panels), **LYS739** (**middle** panels), and **LYS744** (**lower** panels); according to the current annotations in the Uniprot database (https://www.uniprot.org, accessed on 22 April 2022). The numbers represent proteins found within each subcellular localization and functional category, *1, **2.

The effect of a higher morphine dose on protein composition in the brain cortex was increased from 15 to 31 proteins (9 were upregulated, 22 downregulated) when compared with a lower dose, Table S2b. The functional significance of these proteins was mainly related to RNA processing (10), DNA processing (6), cytoskeletal changes (5), transport (4), and apoptosis (4), Figure 9a,b (upper panels). Among the ten morphine-regulated proteins involved in RNA processes were: Mtr4 exosome RNA helicase (*Mtrex*, ↑3.2-fold), thyroid hormone receptor-associated protein 3 (*Thrap3*, ↑2.5-fold), cyclin-dependent kinase 17 (*Cdk17*, ↑2.2-fold), lamina-associated polypeptide 2, isoform beta (*Tmpo*, ↓7.3-fold), A-kinase anchor protein 8 (*Akap8*, ↓7.2-fold), *Snrnp70* (↓7.1-fold), non-histone chromosomal protein HMG-17 (*Hmgn2*, ↓3.2-fold), *Ddx46* (↓2.8-fold), *Mecp2* (↓2.2-fold), and UPF3B, regulator of nonsense mediated mRNA decay (*Upf3b*, ↓2.1-fold). The results of Wu et al. [35] revealed that dependence-associated genes and transcription regulators have essential roles in opioid-induced analgesia and tolerance. Morphine regulates the expression of transcription factors, such as CREB, ΔFosB, and nuclear factor-kappa B (NF-κB). Of particular interest in the study of addiction is ΔFosB, which is expressed in the brain after acute drug treatment but can persist for weeks or even months after the drug is withdrawn. It represents a mechanism by which drugs of abuse produce lasting changes in gene expression patterns long after the cessation of drug administration [21,36].

**LYS739** treatment unveiled 152 altered cortical proteins, which is 4.9 times more than morphine and 7.6 times more than **LYS739** lower dose. Interestingly, only 30 proteins exhibited significant upregulation, whereas 122 proteins were downregulated, Table S2b. These proteins were described to participate in RNA processes (41), transport-related processes (27), organization of the cytoskeleton (13), DNA processes (10), cell cycle (9), signal transduction (9), brain development (9), metabolic changes (7), immunity (6), apoptosis-related pathways (5), protein folding (5), protein ubiquitination (4), cell adhesion (3), ion homeostasis (3), aging (2), protein localization (1), and cell proliferation (1), Figure 9a,b (middle panels). The high number of proteins exhibiting the decreased protein expression levels suggests a state of disturbed cell homeostasis. Notably, decreased level of ubiquitin carboxyl-terminal hydrolase 30 (*Usp30*, ↓2.6-fold), small ubiquitin-related modifier 2 (*Sumo2*, ↓2.3-fold), Ube 2l3 protein (*Ube2l3*, ↓2.1-fold), and UBX domain-containing protein 1 (*Ubxn1*, ↓2.1-fold) may indicate attenuation of protein degradation pathways in the etiology of opioid dependence and addiction [37].

LFQ analysis revealed 26 altered proteins in the brain cortex after **LYS744** treatment. This is a similar number to morphine-induced alterations (31) and, noticeably, less than after the administration of the lower dose of **LYS744** (31). Six proteins were upregulated and 20 were downregulated, Table S2b. According to the current annotations in the Uniprot database, these proteins were associated with RNA processing (7), DNA processing (5), cytoskeletal changes (4), transport (3), brain development (3), metabolism (3), apoptosis (2), aging (2), cell cycle (1), immunity (1), protein folding (1), cell adhesion (1), and protein localization (1), Figure 9a,b (lower panels). *S100a9* was the most downregulated protein (↓5.4-fold), which was found to decrease in cortical samples after morphine administration (↓13.2-fold) and treatment with **LYS739** (↓13.4-fold) as well. Apart from its role in inflammation and apoptosis [25], Toleikis et al. [38] described its involvement in neurodegenerative disorders, such as Alzheimer’s and Parkinson’s diseases. Their data have shown that even small concentrations of *S100a9* resulted in the aggregation and formation of α-synuclein (*Snca*) fibrils. *Snca* is the main protein found in amyloid plaques in the brains of patients suffering from Parkinson’s disease. Volcano plots representing significantly altered proteins identified in rat brain cortex after treatment with 10 mg/kg of morphine, **LYS739**, and **LYS744** are depicted in Figure 7B. Hierarchical heatmap clustering of all identified protein expression profiles in rat brain cortex is presented in Figure 8.

### 3.6. LFQ Analysis of Rat Hippocampus after 7-Day Treatment with Morphine, **LYS739**, and **LYS744**: The Dose-Effect of 10 mg/kg

**Figure 10:**
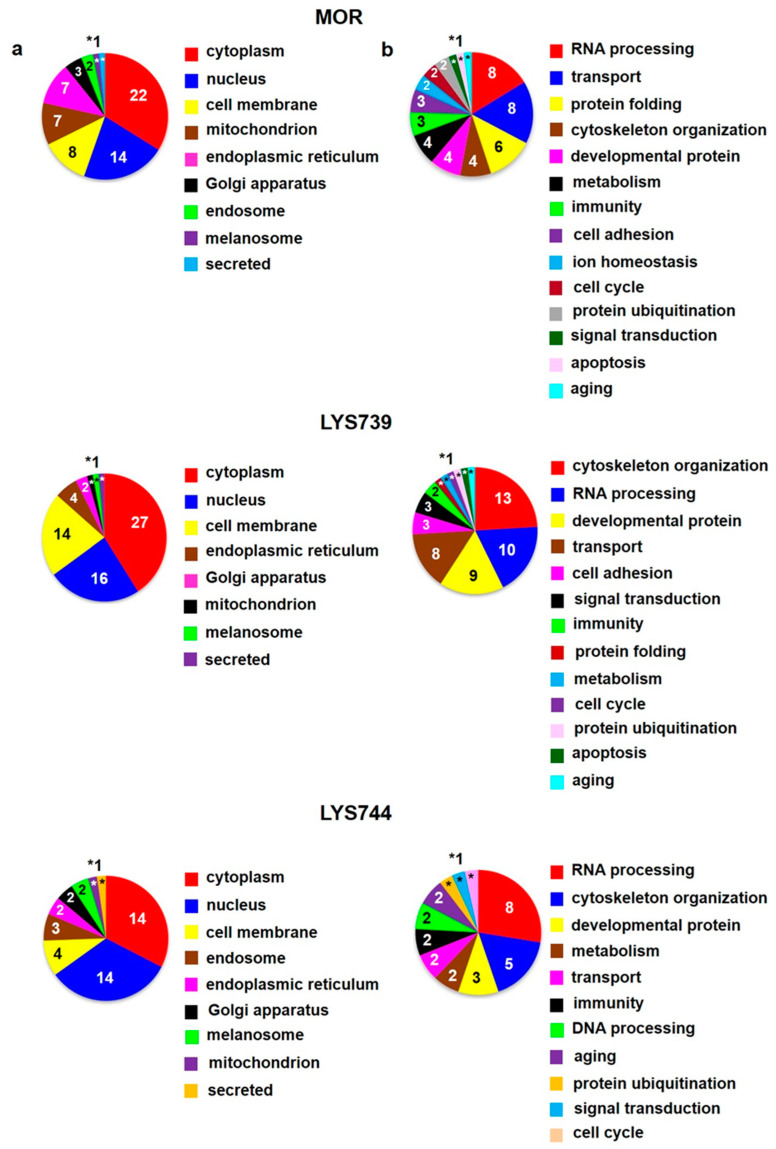
Subcellular localization (**a**) and function (**b**) of significantly altered proteins (at least 2-fold) identified in rat hippocampus after 7-day treatment with 10 mg/kg of **morphine** (**MOR**, **upper** panels), **LYS739** (**middle** panels), and **LYS744** (**lower** panels); according to the current annotations in the Uniprot database (https://www.uniprot.org, accessed on 22 April 2022). The numbers represent proteins found within each subcellular localization and functional category, *1.

Proteomic analysis unveiled 42 altered proteins in rat hippocampus after the treatment with the higher morphine dose; 8 proteins were upregulated, 34 were downregulated, Table S2c. This is three times more than the effect of a lower morphine dose (14). The functional significance of hippocampal proteins was mainly related to RNA processes (8), transport (8), protein folding (6), cytoskeletal changes (4), brain development (4), metabolic changes (4), immunity (3), and cell adhesion (3), Figure 10a,b (upper panels).

Interestingly, 6 decreased proteins were identified as protein chaperones: tubulin-specific chaperone A (*Tbca*, ↓3.7-fold), BAG cochaperone 3 (*Bag3*, ↓2.4-fold), H2-K region express gene 2, rat orthologue (*Pfdn6*, ↓2.2-fold), HscB mitochondrial iron-sulfur cluster co-chaperone (*Hscb*, ↓2.1-fold), RING-type E3 ubiquitin transferase (*Stub1*, ↓2.0-fold), and mitochondrial import inner membrane translocase subunit Tim13 (*Timm13*, ↓2.0-fold). Molecular chaperones prevent protein misfolding and aggregation-induced neurotoxicity. A growing body of evidence suggests that they play a significant role in neuron degeneration and may participate in Parkinson’s disease, Alzheimer’s disease, and Huntington’s disease [39]. Okuyama et al. [40] described that the accumulation of misfolded proteins in the endoplasmic reticulum (ER) induced the unfolded protein response (UPS) contributing to diverse pathological conditions, such as opioid tolerance development. They investigated that pharmacological chaperones 4-phenylbutyric acid (PBA) and tauroursodeoxycholic acid (TUDCA) suppressed the development of morphine tolerance and restored analgesia. Of note is an increased level of proteins involved in cell adhesion and maintenance of tissue integrity: desmoplakin (*Dsp*, ↑7.0-fold), junction plakoglobin (*Jup*, ↑2.3-fold), and integrin beta (*Itgb8*, ↑2.1-fold). Civciristov et al. [41] reported that the stimulation of µ-OR caused an enrichment of proteins associated with the formation of desmosomes.

**LYS739** treatment resulted in the change of 45 proteins, upregulation was found for 20 proteins, 25 proteins were downregulated, Table S2c. When compared with the brain cortex (152), the number of altered proteins in the hippocampus was 3.4 times lower. These proteins were predominantly involved in the cytoskeletal dynamics (13), RNA processing (10), brain development (9), transport (8), and cell adhesion (3), Figure 10a,b (middle panels). Among identified cytoskeletal proteins were: neurofilament light polypeptide (*Nefl*, ↑2.9-fold), tropomyosin alpha-4 chain (*Tpm4*, ↑2.8-fold), Ras/Rap GTPase-activating protein SynGAP (*Syngap1*, ↑2.6-fold), tropomyosin alpha-3 chain (*Tpm3*, ↑2.6-fold), neurofilament medium polypeptide (*Nefm*, ↑2.2-fold), hematopoietic cell-specific LYN substrate 1 (*Hcls1*, ↑2.1-fold), nuclear mitotic apparatus protein 1 (*Numa1*, ↓11.7-fold), myosin-11 (*Myh11*, ↓10.1-fold), vimentin (*Vim*, ↓7.2-fold), transgelin (*Tagln*, ↓7.1-fold), filamin A (*Flna*, ↓2.7-fold), non-muscle caldesmon (*Cald1*, ↓2.1-fold), and microtubule-associated protein (*Map2*, ↓2.0-fold). Neuronal plasticity is required to respond to external stimuli and is achieved by cytoskeletal reorganization controlled by phosphorylation of cytoskeletal proteins [42]. Rothenfluh and Covan [43] discussed that cytoskeleton plays an essential role in neuronal, dendritic, and behavioral plasticity observed in addicted animals. Drastichova et al. [44] identified altered phosphorylated proteins associated with microtubule dynamics and the actin-spectrin network in rat brains isolated from animals even after six months of morphine withdrawal.

The effect of a higher **LYS744** dose on hippocampal proteome was surprisingly decreased from 42 to 26 proteins (6 were upregulated, 20 downregulated) when compared with the lower dose, Table S2c. The functional significance of these proteins was associated with RNA processing (8), cytoskeletal changes (5), brain development (3), metabolism (2), transport (2), immunity (2), DNA processing (2), aging (2), protein ubiquitination (1), signal transduction (1), and cell cycle (1), as shown in Figure 10a,b (lower panels). Cytoskeletal proteins: *Myh11* (↓15.0-fold), *Vim* (↓10.4-fold), *Tagln* (↓4.6-fold), *Flna* (↓2.6-fold), and *Cald1* (↓2.3-fold) were found dysregulated after **LYS739** treatment as well, see above. Volcano plots representing significantly altered proteins identified in rat hippocampus after treatment with 10 mg/kg of morphine, **LYS739**, and **LYS744** are depicted in Figure 7C. Hierarchical heatmap clustering of all identified protein expression profiles in the rat hippocampus is presented in Figure 8.

### 3.7. Summary of Results

#### 3.7.1. Effects of Low Doses of Morphine, **LYS739**, and **LYS744** (3 mg/kg)

LFQ analysis revealed 27 altered proteins in rat *spleen lymphocytes* after 7-day treatment of male rats with 3 mg/kg of morphine. The 12 proteins were upregulated, downregulation was detected for 15 proteins. Their subcellular localization and functional significance unveiled most proteins to be of cytoplasmic origin and functionally related to the organization of cytoskeleton. Fewer proteins were altered by **LYS739**, 5 proteins were upregulated, 15 downregulated. **LYS744** induced more alterations than morphine, 29 proteins were upregulated, and 16 proteins were downregulated. More than half of these proteins were functionally related to actin cytoskeleton organization (11), RNA processing (8), and transport (6).

In the *brain cortex*, morphine administration resulted in the change of 15 proteins; 5 proteins were upregulated, 10 proteins were downregulated. Contrary to the spleen lymphocytes, **LYS739** treatment resulted in a greater number of protein alterations than morphine, with 7 proteins upregulated and 13 downregulated. As in the case of spleen lymphocytes, treatment with **LYS744** exhibited the highest number of alterations in cortical proteome—11 proteins were upregulated, 20 proteins were downregulated.

In the *hippocampus*, LFQ analysis revealed 14 proteins altered by morphine, 8 proteins were upregulated, 6 were downregulated. This change was similar to that found in the brain cortex (15) but lower twice in comparison with spleen lymphocytes (27). The effect of **LYS739** on rat hippocampal protein profile was stronger than that of morphine with 8 proteins upregulated and 28 downregulated. Treatment with **LYS744** unveiled 42 alterations in protein expression levels, which is more than in the brain cortex (31) and similar to that found in spleen lymphocytes (45); 24 proteins were upregulated and 18 proteins were downregulated.

#### 3.7.2. Effects of Higher Doses of Morphine, **LYS739**, and **LYS744** (10 mg/kg)

Proteomic analysis revealed 45 altered proteins in *spleen lymphocytes* after morphine treatment. Upregulation was found for 29 proteins; 16 proteins were downregulated. Their molecular functions and biological processes were mostly involved in transport (9), immunity (8), apoptosis (8), and RNA processing (7). When compared with the effect of a lower dose, the number of proteins associated with immune responses and apoptotic processes was increased, suggesting the state of cell dysfunction and neuroinflammation after chronic morphine administration. **LYS739** treatment resulted in the change of 119 proteins, 51 proteins were upregulated, 68 downregulated. Thus, the number of altered proteins was six times higher than that obtained after the administration of a lower dose. The majority of altered proteins were related to RNA processing (40), transport (16), immunity (11), metabolism (10), and cytoskeletal changes. Higher dose of **LYS744** caused the most significant change in protein expression levels of spleen lymphocytes. Four times more proteins were altered (182 proteins, 88 were upregulated, 94 downregulated) in comparison with the lower dose of **LYS744**. The identified proteins were primarily associated with RNA processing (58), transport (25), immune responses (16), metabolic changes (13), cytoskeletal changes (13), and apoptotic processes (12).

In the *brain cortex*, the effect of a higher morphine dose on protein composition was increased from 15 to 31 proteins (9 proteins were upregulated, 22 downregulated) when compared with a lower dose. The functional significance of these proteins was related to RNA processing (10), DNA processing (6), cytoskeletal changes (5), transport (4), and apoptosis (4). **LYS739** treatment unveiled 152 altered cortical proteins, which is 4.9 times more than morphine and 7.6 times more than **LYS739** lower dose. Interestingly, only 30 proteins exhibited significant upregulation, 122 proteins were downregulated. These proteins were described to participate in RNA processes (41), transport-related processes (27), organization of the cytoskeleton (13), DNA processes (10), cell cycle (9), signal transduction (9), brain development (9), metabolic changes (7), immunity (6), apoptosis-related pathways (5), protein folding (5), protein ubiquitination (4), cell adhesion (3), ion homeostasis (3), aging (2), protein localization (1), and cell proliferation (1). The high number of proteins exhibiting the decreased expression levels suggests a state of disturbed cell homeostasis. **LYS744** treatment resulted in the change of 26 proteins, with 6 proteins upregulated and 20 downregulated.

In the *hippocampus*, proteomic analysis unveiled 42 altered proteins after the treatment with the higher morphine dose, 8 proteins were upregulated, 34 were downregulated. This is three times more than the effect of a lower morphine dose (14). The functional significance of hippocampal proteins was mainly related to RNA processes (8), transport (8), protein folding (6), cytoskeletal changes (4), brain development (4), metabolic changes (4), immunity (3), and cell adhesion (3). **LYS739** treatment resulted in the change of 45 proteins, upregulation was found for 20 proteins, 25 proteins were downregulated. When compared with the brain cortex (152), the number of altered proteins in the hippocampus was 3.4 times lower. The altered proteins were predominantly involved in the cytoskeletal dynamics (13), RNA processing (10), brain development (9), transport (8), and cell adhesion (3). The effect of a higher dose of **LYS744** on hippocampal proteome was surprisingly decreased from 42 to 26 proteins when compared with the lower dose (6 proteins were upregulated, 20 downregulated). The functional significance of these proteins was associated with RNA processing (8), cytoskeletal changes (5), brain development (3), metabolism (2), transport (2), immunity (2), DNA processing (2), aging (2), protein ubiquitination (1), signal transduction (1), and cell cycle (1).

## 4. Conclusions

Overall, 7-day treatment with 3 mg/kg of **LYS744** caused the most significant effect on altered proteins (at least 2-fold) in spleen lymphocytes, rat brain cortex, and hippocampus in comparison with morphine and **LYS739** treatment. When administered a higher dose (10 mg/kg), the number of alterations in lymphocytes was increased by treatment with both analogs, **LYS739** and **LYS744**. The higher dose of **LYS739** caused extensive protein alterations in cortical samples and slightly increased the number of altered proteins in the rat hippocampus. Considering its specific multifunctional activities (µ-OR/δ-OR agonism and κ-OR partial antagonism), our results indicate the possible involvement of multiple opioid receptors in protein regulations in these brain areas. However, the higher dose of **LYS744** did not increase the number of altered proteins in the brain cortex and hippocampus. For the same reason, the κ-OR antagonism of **LYS744** seems to be related to the reduced number of protein alterations in the central nervous system areas. Interestingly, the effect of κ-OR antagonism of **LYS744** was opposite in the peripheral region (spleen lymphocytes).

## Data Availability

Data are contained within the article and its Supplementary Files.

## Supplementary Materials

The following supporting information can be downloaded at: https://www.mdpi.com/article/10.3390/biomedicines10081969/s1, Table S1: Subcellular localization and function of altered proteins isolated from rat spleen lymphocytes, brain cortex, and hippocampus after 7-day treatment with morphine, **LYS739** and **LYS744** (3 mg/kg) identified by label-free quantification (MaxLFQ); Table S2: Subcellular localization and function of altered proteins isolated from rat spleen lymphocytes, brain cortex, and hippocampus after 7-day treatment with morphine, **LYS739** and **LYS744** (10 mg/kg) identified by label-free quantification (MaxLFQ).
