# Supplementary material for: The Dose-Dependent Effects of Multifunctional Enkephalin Analogs on the Protein Composition of Rat Spleen Lymphocytes, Cortex, and Hippocampus; Comparison with Changes Induced by Morphine
Source: Biomedicines. 2022 Aug 14;10(8):1969. doi: 10.3390/biomedicines10081969 (PMC9406115; doi:10.3390/biomedicines10081969)
Supplement: Supplementary file 1 [file biomedicines-10-01969-s001.zip › Table S1.pdf]

Table S1:

a) Subcellular localization and function of altered proteins isolated from **rat spleen lymphocytes** after 7-day treatment with **morphine**, **LYS739** and **LYS744** (***3 mg/kg***) identified by label-free quantification (MaxLFQ).

Accession number	Protein name	Gene	Change (fold)	p value	Subcellular localization	Molecular functions and biological processes- keywords
**Lymphocytes -MORPHINE – 3 mg/kg, 7 days:**						
UP-regulated						
**1**-Q63633	Solute carrier family 12 member 5	**Slc12a5**	↑41	0.0008	Cell membrane	Ion transport
**2**-Q05764	Beta-adducin	**Add2**	↑4.0	0.0076	Cell membrane, cytoskeleton	Actin filament bundle assembly
**3**-Q9QWN8	Spectrin beta chain, non-erythrocytic 2	**Sptbn2**	↑3.9	0.0280	Cytoplasm, cytoskeleton	Actin filament capping
**4**-Q6AZ54	Hemogen	**Hemgn**	↑3.4	<0.0001	Nucleus	Cell differentiation
**5**-P40615	H/ACA ribonucleoprotein complex subunit 4	**Dkc1**	↑2.6	0.0095	Nucleus	RNA processing
**6**-Q5PPN5	Tubulin polymerization-promoting protein family member 3	**Tppp3**	↑2.6	0.0189	Cytoplasm, cytoskeleton	Microtubule polymerization
**7**-P35465	Serine/threonine-protein kinase PAK 1	**Pak1**	↑2.4	0.0255	Cytoplasm, cell membrane, nucleus	Apoptosis, exocytosis
**8**-P24135	1-phosphatidylinositol 4,5-bisphosphate phosphodiesterase gamma-2	**Plcg2**	↑2.4	<0.0001	Cytoplasm, cell membrane	Signal transduction, lipid metabolism
**9**-A0A140UHX6	Spectrin beta chain	**Sptbn2**	↑2.0	0.0199	Cytoplasm, cytoskeleton	Actin filament capping
**10**-Q99JD4	CLIP-associating protein 2	**Clasp2**	↑2.0	0.0127	Cell membrane, cytoplasm, GA*	Cell cycle
**11**-D3Z9Z0	Ankyrin-1	**Ank1**	↑2.0	0.0131	Cytoskeleton, ER**, nucleus	Vesicle-mediated transport
**12**-B0BNN3	Carbonic anhydrase 1	**Ca1**	↑2.0	<0.0001	Cytoplasm	Reversible hydration of carbon dioxide
Down-regulated						
**1**-P30337	N-chimaerin	**Chn1**	↓12.3	0.0386	Cytoplasm	Signal transduction, neurogenesis
**2**-Q4QRB4	Tubulin beta-3 chain	**Tubb3**	↓5.1	0.0399	Cytoplasm, cytoskeleton	Cytoskeleton organization
**3**-P11275	Calcium/calmodulin-dependent protein kinase type II subunit alpha	**Camk2a**	↓4.7	0.0389	Cell junction, synapse	Calcium ion transport, protein phosphorylation
**4**-P31596	Excitatory amino acid transporter 2	**Slc1a2**	↓4.3	0.0049	Cell membrane	Amino-acid transport
**5**-P0C170	Histone H2A type 1-E	**N/A**	↓3.7	0.0073	Nucleus	DNA processing
**6**-D4A7H9	Charged multivesicular body protein 7	**Chmp7**	↓2.9	0.0360	Cytoplasm, endosome, nucleus	Transport, nucleus organization
**7**-Q62950	Dihydropyrimidinase-related protein 1	**Crmp1**	↓2.8	0.0252	Cytoplasm, cytoskeleton	Cytoskeleton organization, axon guidance
**8**-P31000	Vimentin	**Vim**	↓2.8	0.0016	Cell membrane, cytoskeleton, nucleus	Aging, intermediate filament organization

**9**-P07151	Beta-2-microglobulin	**B2m**	↓2.7	0.0017	Secreted	Immunity
**10**-P63312	Thymosin beta-10	**Tmsb10**	↓2.7	0.0134	Cytoplasm, cytoskeleton	Cytoskeleton organization
**11**-P10960	Sulfated glycoprotein 1	**Psap**	↓2.7	0.0171	Lysosome	Glycosphingolipid metabolism, signal transduction
**12**-P09495	Tropomyosin alpha-4 chain	**Tpm4**	↓2.3	0.0242	Cytoplasm, cytoskeleton	Actin filament organization
**13**-D4AD33	RNA guanine-7 methyltransferase-activating subunit	**Ramac**	↓2.2	0.0014	Nucleus	RNA processing
**14**-Q64122	Myosin regulatory light polypeptide 9	**Myl9**	↓2.0	0.0055	Cytoplasm, cytoskeleton	Motor protein, cell locomotion
**15**-Q5BK20	Jupiter microtubule associated homolog 2	**Jpt2**	↓2.0	0.0033	Cytoplasm, nucleus	Protein phosphorylation

Lymphocytes -LYS739 – 3 mg/kg, 7 days

UP-regulated

**1**-D3ZSY4	Eosinophil peroxidase	**Epx**	↑2.7	0.0002	Secreted	Heme binding, oxidative stress response
**2**-P13832	Myosin regulatory light chain RLC-A	**Rlc-a**	↑2.5	0.0031	Cytoplasm, cytoskeleton	Motor protein, regulation of cell shape
**3**-P40615	H/ACA ribonucleoprotein complex subunit 4	**Dkc1**	↑2.2	0.0149	Nucleus	RNA processing
**4**-Q4FZX5	Methionine-R-sulfoxide reductase B2, mitochondrial	**Msrb2**	↑2.2	0.0233	Mitochondrion	Protein repair, oxidative stress response
**5**-Q925G1	Hepatoma-derived growth factor-related protein 2	**Hdgfrp2**	↑2.0	0.0184	Nucleus	Regulation of cell growth, RNA processing

Down-regulated

**1**-P31596	Excitatory amino acid transporter 2	**Slc1a2**	↓4.0	0.0289	Cell membrane	Amino-acid transport
**2**-P09812	Glycogen phosphorylase, muscle form	**Pygm**	↓4.0	0.0043	ER, cytoplasm	Glycogen metabolism
**3**-D4A206	Treacle ribosome biogenesis factor 1	**Tcof1**	↓3.0	0.0028	Cytoplasm, nucleus	Regulation of translation
**4**-P09951	Synapsin-1	**Syn1**	↓2.7	0.0323	GA	Neurotransmitter release cycle
**5**-P02401	60S acidic ribosomal protein P2	**Rplp2**	↓2.6	<0.0001	Cytoplasm, ribosome	Regulation of translation
**6**-A0A0G2K2M9	Serine/arginine repetitive matrix 2	**Srrm2**	↓2.6	0.0086	Nucleus	RNA processing
**7**-P08081	Clathrin light chain A	**Clta**	↓2.4	0.0016	Cytoplasm, cytoskeleton, cell membrane	Cell cycle, protein transport
**8**-D4A4P3	Complex I-B12	**Ndufb3**	↓2.3	0.0079	Mitochondrion	Electron transport chain
**9**-Q66HF8	Aldehyde dehydrogenase X, mitochondrial	**Aldh1b1**	↓2.2	0.0222	Mitochondrion	Ethanol oxidation, lipid peroxidation
**10**-P07151	Beta-2-microglobulin	**B2m**	↓2.2	0.0034	Secreted	Immunity
**11**-P09495	Tropomyosin alpha-4 chain	**Tpm4**	↓2.1	0.0307	Cytoplasm, cytoskeleton	Actin filament organization
**12**-Q3LRZ1	CREB-regulated transcription coactivator 2	**Crtc2**	↓2.0	0.0175	Cytoplasm, nucleus	Regulation of transcription
**13**-A1L1K4	CDC42 small effector protein 2	**Cdc42se2**	↓2.0	0.0425	Cell membrane, cytoplasm, cytoskeleton	Cell shape, signal transduction
**14**-P10960	Sulfated glycoprotein 1	**Psap**	↓2.0	0.0183	Lysosome	Glycosphingolipid metabolism, signal transduction

**15**-F1LNI5	Protein phosphatase 1G	**Ppm1g**	↓2.0	0.0450	Cytoplasm, cell membrane	Cell cycle

Lymphocytes -LYS744 – 3 mg/kg, 7 days

UP-regulated

**1**-E9PU01	DNA helicase	**Chd4**	↑3.8	0.0411	Nucleus, cytoplasm	Chromatin organization
**2**-P14046	Alpha-1-inhibitor 3	**A1i3**	↑3.5	0.0178	Secreted	Protease inhibitor
**3**-P97710	Tyrosine-protein phosphatase non-receptor type substrate 1	**Sirpa**	↑3.3	0.0038	Cell membrane	Cell-matrix adhesion, cytoskeleton organization
**4**-D3ZKT0	Phosphatidate cytidylyltransferase, mitochondrial	**Tamm41**	↑2.7	0.0454	Cell membrane, mitochondrion	Lipid metabolism
**5**-Q64548	Reticulon-1	**Rtn1**	↑2.6	0.0339	ER membrane, GA membrane	Regulation of amyloid-beta formation
**6**-D3ZFD0	Unconventional myosin-XVIIIa	**Myo18a**	↑2.4	0.0043	Cytoplasm, GA	Motor protein, Golgi organization
**7**-A0A140UHX6	Spectrin beta chain	**Sptb**	↑2.4	0.0076	Cytoplasm, cytoskeleton	Actin capping
**8**-D3Z9Z0	Ankyrin 1	**Ank1**	↑2.4	0.0065	Cytoskeleton, cell membrane, ER, nucleus	ER to Golgi vesicle-mediated transport
**9**-Q63355	Unconventional myosin-Ic	**Myo1c**	↑2.4	0.0190	Cell membrane, cytoplasm	Protein transport, motor protein
**10**-Q9WU82	Catenin beta-1	**Ctnnb1**	↑2.4	0.0353	Cell membrane, cytoplasm, nucleus	Cell adhesion, neurogenesis
**11**-F1LP64	E3 ubiquitin-protein ligase TRIP12	**Trip12**	↑2.3	0.0010	Nucleus	DNA processing
**12**-B2RYU7	Cbx5 protein	**Cbx5**	↑2.3	0.0019	Nucleus	Regulation of transcription
**13**-Q66HG8	Protein Red	**Ik**	↑2.3	0.0136	Cytoplasm, cytoskeleton, nucleus	RNA processing
**14**-P02688	Myelin basic protein	**Mbp**	↑2.3	0.0004	Cell membrane	Aging, myelination
**15**-D4ABI7	Very-long-chain (3R)-3-hydroxyacyl-CoA dehydratase	**Hcd3**	↑2.3	0.0058	ER, cell membrane	Lipid metabolism
**16**-P09951	Synapsin-1	**Syn1**	↑2.3	0.0291	GA	Neurotransmitter release cycle
**17**-B2GUY4	Dematin actin-binding protein	**Dmtn**	↑2.3	0.0098	Cytoplasm, cytoskeleton, cell membrane	Actin cytoskeleton organization
**18**-Q5RKH0	Putative oxidoreductase GLYR1	**Glyr1**	↑2.2	0.0071	Nucleus	Regulation of transcription
**19**-Q9QYF3	Unconventional myosin-Va	**Myo5a**	↑2.2	0.0104	Cytoplasm, cytoskeleton, GA, ER, endosome	Actin filament organization, protein transport
**20**-O35274	Neurabin-2	**Ppp1r9b**	↑2.2	0.0164	Cell membrane, cytoplasm, nucleus	Actin filament organization, neurogenesis
**21**-Q6P9V9	Tubulin alpha-1B chain	**Tuba1b**	↑2.2	0.0329	Cytoplasm, cytoskeleton	Cytoskeleton organization
**22**-Q5XIB5	Coiled-coil domain-containing protein 86	**Ccdc86**	↑2.2	0.0002	Nucleus	Citrullinated by PADI4
**23**-P22985	Xanthine dehydrogenase/oxidase	**Xdh**	↑2.2	0.0496	Cytoplasm, peroxisome, secreted	Purine degradation, aging
**24**-Q63356	Unconventional myosin-Ie	**Myo1e**	↑2.1	0.0028	Cytoplasmic vesicle, cytoskeleton	Actin filament organization, endocytosis
**25**-P13832	Myosin regulatory light chain RLC-A	**Rlc-a**	↑2.0	0.0116	Cytoplasm, cytoskeleton	Regulation of cell shape, motor protein
**26**-P55009	Allograft inflammatory factor 1	**Aif1**	↑2.0	0.0115	Cell membrane, cytoplasm, cytoskeleton	Actin filament polymerization, inflammatory response
**27**-B2RYI2	Signal recognition particle subunit SRP68	**Srp68**	↑2.0	0.0406	Cytoplasm	Protein targeting to the rough ER membrane
**28**-D3ZSY4	Eosinophil peroxidase	**Epx**	↑2.0	0.0002	Secreted	Neutrophil degranulation, oxidative stress response

**29**-Q6AYA2	VRK serine/threonine kinase 1	**Vrk1**	↑2.0	0.0013	Cytoplasm, nucleus, GA	Nuclear envelope breakdown, Golgi disassembly
Down-regulated						
**1**-A0A0G2JSV6	Globin c2	**Hba-a2**	↓11.6	0.0052	Cytoplasm, secreted	Oxygen transport
**2**-P13596	Neural cell adhesion molecule 1	**Ncam1**	↓4.5	0.0148	Cell membrane	Cell adhesion, aging
**3**-Q05175	Brain acid soluble protein 1	**Basp1**	↓4.5	0.0028	Cell membrane	Regulation of transcription
**4**-P63219	Guanine nucleotide-binding protein G(I)/G(S)/G(O) subunit gamma-5	**Gng5**	↓3.3	0.0266	Cell membrane	Signal transduction
**5**-Q5BK20	Jupiter microtubule associated homolog 2	**Jpt2**	↓3.1	<0.0001	Cytoplasm, nucleus	Protein phosphorylation
**6**-D4AD33	RNA guanine-7 methyltransferase-activating subunit	**Ramac**	↓2.7	<0.0001	Nucleus	RNA processing
**7**-Q6IN36	WAS/WASL-interacting protein family member 1	**Wipf1**	↓2.4	0.0086	Cytoplasm, cytoskeleton	Actin cytoskeleton organization
**8**-D4A7H9	Charged multivesicular body protein 7	**Chmp7**	↓2.4	0.0361	Cytoplasm, endosome, nucleus	Transport, nucleus organization
**9**-Q62806	Zinc finger protein 148	**Znf148**	↓2.4	0.0009	Nucleus	Regulation of transcription
**10**-Q03344	ATPase inhibitor, mitochondrial	**Atpif1**	↓2.3	0.0003	Mitochondrion	Regulation of ATP metabolic process
**11**-P18291	Granzyme B	**Gzmb**	↓2.2	0.0018	Lysosome, secreted	Apoptosis
**12**-Q792Q4	Cysteine-rich PDZ-binding protein	**Cript**	↓2.2	0.0135	Cytoplasm, cell junction	Protein localization
**13**-A0A0G2K2M9	Serine/arginine repetitive matrix 2	**Srrm2**	↓2.2	0.0004	Nucleus	RNA processing
**14**-O08769	Cyclin-dependent kinase inhibitor 1B	**Cdkn1b**	↓2.1	0.0203	Cytoplasm, nucleus, endosome	Apoptosis, response to drug
**15**-M0R9Z5	Interferon regulatory factor 2-binding protein 2	**Irf2bp2**	↓2.1	0.0174	Nucleus	Regulation of transcription
**16**-G3V7E3	Granzyme A	**Gzma**	↓2.0	<0.0001	Nucleus	Apoptosis

b) Subcellular localization and function of altered proteins isolated from **rat brain cortex** after 7-day treatment with **morphine**, **LYS739** and **LYS744** (***3 mg/kg***) identified by label-free quantification (MaxLFQ).

Accession number	Protein name	Gene	Change (fold)	p value	Subcellular localization	Molecular functions and biological processes- keywords
**CORTEX -MORPHINE – 3 mg/kg, 7 days**						
UP-regulated						
**1**-D3Z8E0	Ribosomal protein S6 kinase	**Rps6ka3**	↑2.7	0.0074	Cytoplasm, nucleus, ribosome	Signal transduction, apoptosis
**2**-M0R9Q1	RNA-binding motif protein 14	**Rbm14**	↑2.7	0.009	Cytoplasm, nucleus	Signal transduction, immunity

**3**-D3ZKT0	Phosphatidate cytidylyltransferase, mitochondrial	**Tamm41**	↑2.1	0.0043	Cell membrane, mitochondrion	Lipid metabolism
**4**-Q99P82	Claudin-11	**Cldn11**	↑2.1	0.0004	Cell membrane	Cell adhesion
**5**-D3ZQ18	NCCRP1, F-box-associated domain-containing	**Nccrp1**	↑2.1	0.0129	Cytoplasm, nucleus	Protein ubiquitination
Down-regulated						
**1**-A0A0G2JSV6	Globin c2	**Hba-a2**	↓13.6	0.0150	Cytoplasm, secreted	Oxygen transport
**2**-P50116	Protein S100-A9	**S100a9**	↓3.7	0.0273	Cell membrane, cytoplasm, secreted	Actin cytoskeleton organization, apoptosis, immunity
**3**-B2RZ74	U1 small nuclear ribonucleoprotein 70 kDa	**Snrnp70**	↓3.5	0.0002	Cytoplasm, nucleus	RNA processing
**4**-D3ZQE8	Exportin 5	**Xpo5**	↓2.4	0.0215	Cytoplasm	Protein export
**5**-P02625	Parvalbumin alpha	**Pvalb**	↓2.4	0.0016	Cytoplasm, nucleus	Calcium-binding protein
**6**-Q9JID1	Programmed cell death protein 4	**Pdcd4**	↓2.3	0.0401	Cytoplasm, nucleus	Apoptosis
**7**-Q4KLZ3	DAZ-associated protein 1	**Dazap1**	↓2.3	0.0058	Cytoplasm, nucleus	RNA processing
**8**-Q63910	Alpha globin	**Hba-a3**	↓2.2	0.0021	Cytoplasm, secreted	Oxygen transport
**9**-E9PU28	Inosine-5-monophosphate dehydrogenase 2	**Impdh2**	↓2.2	0.0017	Cytoplasm, nucleus	Purine biosynthesis, RNA/DNA metabolism
**10**-Q3B8Q1	Nucleolar RNA helicase 2	**Ddx21**	↓2.2	0.0068	Cytoplasm, nucleus, mitochondrion	Immunity, RNA processing

CORTEX -LYS739 – 3 mg/kg, 7 days

UP-regulated						
**1**-C0JPT7	Filamin A	**Flna**	↑3.5	0.0427	Cell membrane, cytoplasm, nucleus, GA	Actin cytoskeleton organization
**2**-D3ZQ18	NCCRP1, F-box-associated domain-containing	**Nccrp1**	↑3.0	0.0033	Cytoplasm	Protein ubiquitination
**3**-A0A0G2JT64	Aldo-keto reductase family 1, member B8	**Akr1b10**	↑2.9	0.0038	Cytoplasm, mitochondrion	Estrogen biosynthesis
**4**-D3ZX38	Prefoldin 1	**Pfdn1**	↑2.4	0.0015	Cytoplasm	Protein folding, actin cytoskeleton organization
**5**-A0A0G2JYU6	Formin-like 1	**Fmnl1**	↑2.3	0.0337	Cell membrane, cytoplasm	Actin cytoskeleton organization
**6**-D3ZKT0	Phosphatidate cytidylyltransferase, mitochondrial	**Tamm41**	↑2.1	0.0059	Cell membrane, mitochondrion	Lipid metabolism
**7**-B4F7A1	Complex III assembly factor LYRM7	**Lyrm7**	↑2.0	0.0040	Mitochondrion	Chaperone
Down-regulated						
**1**-P50116	Protein S100-A9	**S100a9**	↓10.5	0.0176	Cell membrane, cytoplasm, secreted	Actin cytoskeleton organization, apoptosis, immunity
**2**-Q00438	Polypyrimidine tract-binding protein 1	**Ptbp1**	↓8.4	0.0078	Nucleus	RNA processing, neurogenesis
**3**-B2RZ74	U1 small nuclear ribonucleoprotein 70 kDa	**Snrnp70**	↓6.5	0.0002	Cytoplasm, nucleus	RNA processing

**4**-D4ABT8	Heterogeneous nuclear ribonucleprotein U-like 2	**Hnrnpul2**	↓5.4	0.0043	Nucleus	RNA binding
**5**-G3V7Q7	IQ motif-containing GTPase-activating protein 1	**Iqgap1**	↓5.2	0.0052	Cell membrane, cytoplasm, nucleus	Actin cytoskeleton organization, cell migration
**6**-F1LRS8	CD2-associated protein	**Cd2ap**	↓4.7	0.0015	Cytoplasm, cytoskeleton	Actin cytoskeleton organization, cell cycle
**7**-Q62780	Probable ATP-dependent RNA helicase DDX46	**Ddx46**	↓4.2	0.0310	Cell membrane, nucleus	RNA processing
**8**-Q5M860	Rho GDP dissociation inhibitor beta	**Arhgdib**	↓3.7	0.0136	Cell membrane, cytoplasm	Signal transduction
**9**-B1WC49	Api5 protein	**Api5**	↓3.2	0.0032	Nucleus	Apoptosis
**10**-Q3B8Q1	Nucleolar RNA helicase 2	**Ddx21**	↓3.2	0.0030	Cytoplasm, nucleus, mitochondrion	Immunity, RNA processing
**11**-G3V7T6	Splicing factor 3b, subunit 1	**Sf3b1**	↓2.2	0.0061	Nucleus	RNA processing
**12**-Q63797	Proteasome activator complex subunit 1	**Psme1**	↓2.0	0.0224	Cytoplasm, nucleus	Antigen processing, cell cycle
**13**-B5DES0	Small nuclear ribonucleoprotein Sm D2	**Snrpd2**	↓2.0	0.0213	Cytoplasm, nucleus	RNA processing

CORTEX -LYS744 – 3 mg/kg, 7 days

UP-regulated

**1**-C0JPT7	Filamin A	**Flna**	↑3.9	0.0099	Cell membrane, cytoplasm, nucleus, GA	Actin cytoskeleton organization
**2**-Q4V8E4	Cilia- and flagella-associated protein 36	**Cfap36**	↑3.0	0.0037	Cytoplasm, nucleus	Protein N-terminus binding
**3**-D3Z8E0	Ribosomal protein S6 kinase	**Rps6ka3**	↑2.9	0.0017	Cytoplasm, nucleus, ribosome	Signal transduction, apoptosis
**4**-D3ZQ18	NCCRP1, F-box-associated domain-containing	**Nccrp1**	↑2.9	0.0011	Cytoplasm	Protein ubiquitination
**5**-D3ZX38	Prefoldin 1	**Pfdn1**	↑2.6	0.0182	Cytoplasm	Protein folding, actin cytoskeleton organization
**6**-D3ZKT0	Phosphatidate cytidylyltransferase, mitochondrial	**Tamm41**	↑2.2	0.0188	Cell membrane, mitochondrion	Lipid metabolism
**7**-A0A0G2JY26	DnaJ heat shock protein family (Hsp40) member C6	**Dnajc6**	↑2.2	0.0230	Cytoplasm, synapse	Clathrin-mediated endocytosis
**8**-Q9JKS6	Protein piccolo	**Pclo**	↑2.1	0.0120	Cell junction, synapse	Regulation of exocytosis
**9**-Q99J82	Integrin-linked protein kinase	**Ilk**	↑2.0	0.0054	Cell membrane, cytoplasm	Signal transduction, aging
**10**-Q5I0K8	28S ribosomal protein S7, mitochondrial	**Mrps7**	↑2.0	0.0024	Mitochondrion	Translation
**11**-O88778	Protein bassoon	**Bsn**	↑2.0	0.0023	Cell junction, cytoplasm, synapse	Retrograde axonal transport

Down-regulated

**1**-Q00438	Polypyrimidine tract-binding protein 1	**Ptbp1**	↓14.4	0.0031	Nucleus	RNA processing, neurogenesis
**2**-P50116	Protein S100-A9	**S100a9**	↓5.4	0.0138	Cell membrane, cytoplasm, secreted	Actin cytoskeleton organization, apoptosis, immunity
**3**-B2RZ74	U1 small nuclear ribonucleoprotein 70 kDa	**Snrnp70**	↓4.4	0.0106	Cytoplasm, nucleus	RNA processing
**4**-F1LRI5	GCN1 activator of EIF2AK4	**Gcn1l1**	↓4.3	0.0102	Cytoplasm	Regulation of translation
**5**-P23562	Band 3 anion transport protein	**Slc4a1**	↓3.5	0.0390	Cell membrane	Ion transport

**6**-Q63798	Proteasome activator complex subunit 2	**Psme2**	↓3.2	0.0309	Cytoplasm, nucleus	Antigen processing, cell cycle
**7**-F1LRS8	CD2-associated protein	**Cd2ap**	↓3.2	0.0172	Cytoplasm, cytoskeleton	Actin cytoskeleton organization, cell cycle
**8**-Q63797	Proteasome activator complex subunit 1	**Psme1**	↓3.0	0.0102	Cytoplasm, nucleus	Antigen processing, cell cycle
**9**-Q5U2Y1	General transcription factor II-I	**Gtf2i**	↓2.9	0.0409	Cytoplasm, nucleus	Regulation of transcription
**10**-A0A0G2JSV6	Globin c2	**Hba-a2**	↓2.6	0.0045	Cytoplasm, secreted	Oxygen transport
**11**-Q62667	Major vault protein	**Mvp**	↓2.4	0.0047	Cytoplasm, nucleus	Signal transduction
**12**-Q4KLZ3	DAZ-associated protein 1	**Dazap1**	↓2.4	0.0267	Cytoplasm, nucleus	RNA processing
**13**-P17475	Alpha-1-antiproteinase	**Serpina1**	↓2.3	0.0009	Secreted	Inflammatory response
**14**-Q5XIE0	Acidic leucine-rich nuclear phosphoprotein 32 family member E	**Anp32e**	↓2.3	0.0461	Cytoplasm, nucleus	Protein folding, apoptosis
**15**-P0C643	RAS guanyl-releasing protein 2	**Rasgrp2**	↓2.3	0.0198	Cell membrane, cytoplasm, synapse	Signal transduction
**16**-P25886	60S ribosomal protein L29	**Rpl29**	↓2.3	0.0013	Ribosome, synapse	Aging, translation
**17**-Q63041	Alpha-1-macroglobulin	**A1m**	↓2.0	0.0003	Secreted	Protease inhibitor
**18**-Q6IMY8	Heterogeneous nuclear ribonucleoprotein U	**Hnrnpu**	↓2.0	0.0031	Nucleus, cytoplasm	RNA processing, cell cycle
**19**-Q3B8Q1	Nucleolar RNA helicase 2	**Ddx21**	↓2.0	0.0093	Cytoplasm, nucleus, mitochondrion	Immunity, RNA processing
**20**-D3ZQG6	Tripartite motif-containing protein 2	**Trim2**	↓2.0	0.0012	Cytoplasm	Protein ubiquitination, apoptosis

c) Subcellular localization and function of altered proteins isolated from **rat hippocampus** after 7-day treatment with **morphine**, **LYS739** and **LYS744** (***3 mg/kg***) identified by label-free quantification (MaxLFQ).

Accession number	Protein name	Gene	Change (fold)	p value	Subcellular localization	Molecular functions and biological processes- keywords
**HIPPOCAMPUS -MORPHINE – 3 mg/kg, 7 days**						
UP-regulated						
**1**-A0A0G2K9C0	Vasodilator-stimulated phosphoprotein	**Vasp**	↑4.1	0.0497	Cytoplasm, cytoskeleton	Actin cytoskeleton organization
**2**-M0R7B4	H1.3 linker histone, cluster member	**Hist1h1d**	↑2.8	<0.0001	Nucleus	DNA processing, nucleus assembly
**3**-P31232	Transgelin	**Tagln**	↑2.7	<0.0001	Cytoplasm	Actin filament binding
**4**-Q00566	Methyl-CpG-binding protein 2	**Mecp2**	↑2.6	0.009	Nucleus	RNA processing
**5**-Q5U2Y1	General transcription factor II-I	**Gtf2i**	↑2.2	0.0213	Cytoplasm, nucleus	Regulation of transcription
**6**-A1A5S2	CXXC-type zinc finger protein 1	**Cxxc1**	↑2.2	0.0210	Nucleus	Regulation of transcription
**7**-P27791	cAMP-dependent protein kinase catalytic subunit alpha	**Prkaca**	↑2.0	0.0441	Cell membrane, cytoplasm, mitochondrion	Signal transduction

**8**-P25886	60S ribosomal protein L29	**Rpl29**	↑2.0	0.0051	Ribosome, synapse	Aging, translation

Down-regulated

**1**-Q6AXS3	Protein DEK	**Dek**	↓2.7	0.0121	Nucleus	Chromatin organization
**2**-A0A0G2JSV6	Globin c2	**Hba-a2**	↓2.7	0.0158	Cytoplasm, secreted	Oxygen transport
**3**-F1LMV6	Desmoplakin	**Dsp**	↓2.5	0.0001	Cytoplasm, cytoskeleton	Desmosome organization, cell-cell adhesion
**4**-Q64542	Plasma membrane calcium-transporting ATPase 4	**Atp2b4**	↓2.5	0.0109	Cell membrane	Calcium transport, hippocampus development
**5**-P23565	Alpha-internexin	**Ina**	↓2.2	0.0015	Cytoplasm, cytoskeleton	Cytoskeleton organization, developmental protein
**6**-Q6P0K8	Junction plakoglobin	**Jup**	↓2.2	0.0003	Cytoplasm, cytoskeleton, cell junction	Cell-cell adhesion, desmosome assembly

HIPPOCAMPUS -LYS739 – 3 mg/kg, 7 days

UP-regulated

**1**-M0R7B4	H1.3 linker histone, cluster member	**Hist1h1d**	↑4.5	<0.0001	Nucleus	DNA processing, nucleus assembly
**2**-Q00566	Methyl-CpG-binding protein 2	**Mecp2**	↑3.9	0.0056	Nucleus	RNA processing
**3**-Q6MG61	Chloride intracellular channel protein 1	**Clic1**	↑3.5	0.0095	Cell membrane, cytoplasm, nucleus	Ion transport
**4**-Q5XIP6	Flap endonuclease 1	**Fen1**	↑3.0	0.0339	Mitochondrion, nucleus	DNA processing
**5**-Q03344	ATPase inhibitor, mitochondrial	**Atpif1**	↑2.5	0.0031	Mitochondrion	Regulation of ATP metabolic process
**6**-D3ZQG6	Tripartite motif-containing protein 2	**Trim2**	↑2.2	<0.0001	Cytoplasm	Protein ubiquitination, apoptosis
**7**-Q62703	Reticulocalbin-2	**Rcn2**	↑2.1	0.0113	Endoplasmic reticulum	Calcium ion binding
**8**-P07150	Annexin A1	**Anxa1**	↑2.0	0.0022	Cell membrane, cytoplasm, endosome, nucleus	Immunity, signal transduction

Down-regulated

**1**-D4A510	SWI/SNF-related, matrix-associated, actin-dependent regulator of chromatin, subfamily c, member 2	**Smarcc2**	↓4.8	0.0364	Nucleus	Regulation of transcription
**2**-C0JPT7	Filamin A	**Flna**	↓4.2	0.0238	Cell membrane, cytoplasm, nucleus, GA	Actin cytoskeleton organization
**3**-Q5FVI4	Cell cycle exit and neuronal differentiation protein 1	**Cend1**	↓3.9	<0.0001	Cell membrane	Neuronal differentiation
**4**-Q62733	Lamina-associated polypeptide 2, isoform beta	**Tmpo**	↓3.7	0.0280	Nucleus	Regulation of transcription
**5**-P16884	Neurofilament heavy polypeptide	**Nefh**	↓3.7	0.0016	Cytoplasm, cytoskeleton	Brain development, cytoskeleton organization
**6**-P37361	Metallothionein-3	**Mt3**	↓3.5	0.0040	Cytoplasm, mitochondrion, nucleus, ER	Ion homeostasis, brain development, oxidative stress
**7**-P24594	Insulin-like growth factor-binding protein 5	**Igfbp5**	↓3.0	0.0025	Secreted	Aging, signal transduction

**8**-B2RYJ4	L-aminoadipate-semialdehyde dehydrogenase-phosphopantetheinyl transferase	**Aasdhppt**	↓3.0	0.0232	Cytoplasm	Vitamin B5 metabolism
**9**-D3ZRG7	RNA-binding motif protein 26	**Rbm26**	↓2.9	<0.0001	Nucleus	RNA processing
**10**-D3ZUV3	Eukaryotic translation initiation factor 2A	**Eif2a**	↓2.8	0.0126	Cytoplasm	Regulation of translation
**11**-P06302	Prothymosin alpha	**Ptma**	↓2.7	0.0099	Nucleus	Cell differentiation, apoptosis
**12**-Q62876	Synaptogyrin-1	**Syngr1**	↓2.7	0.0013	Melanosome	Exocytosis
**13**-P49806	Regulator of G-protein signaling 10	**Rgs10**	↓2.6	0.0032	Cytoplasm, nucleus	Signal transduction
**14**-Q91XQ4	DNA-directed RNA polymerase II subunit GRINL1A	**Polr2m**	↓2.6	0.0087	Nucleus	Regulation of transcription, signal transduction
**15**-D4AAZ6	60S ribosomal protein L37a	**Rpl37a**	↓2.5	0.0100	Cytoplasm	Regulation of translation
**16**-Q2LAP6	Testin	**Tes**	↓2.4	0.0114	Cytoplasm	Cell proliferation, cell adhesion
**17**-P02625	Parvalbumin alpha	**Pvalb**	↓2.4	0.0014	Cytoplasm, nucleus	Calcium-binding protein
**18**-P05505	Cytochrome c oxidase subunit 3	**Mtco3**	↓2.3	0.0331	Mitochondrion	Respiratory electron transport
**19**-P63055	Purkinje cell protein 4	**Pcp4**	↓2.3	0.0188	Cytoplasm, cytoskeleton	Signal transduction, apoptosis
**20**-Q63186	Translation initiation factor eIF-2B subunit delta	**Eif2b4**	↓2.2	0.0274	Cytoplasm	Regulation of translation, brain development
**21**-Q925G1	Hepatoma-derived growth factor-related protein 2	**Hdgfrp2**	↓2.2	0.0192	Nucleus	Regulation of cell growth, RNA processing
**22**-B5DES0	Small nuclear ribonucleoprotein Sm D2	**Snrpd2**	↓2.1	0.0217	Cytoplasm, nucleus	RNA processing
**23**-Q63910	Alpha globin	**Hba-a3**	↓2.1	0.0007	Cytoplasm, secreted	Oxygen transport
**24**-D3ZXH7	Aly/REF export factor	**Alyref**	↓2.1	0.0192	Cytoplasm, nucleus	RNA processing
**25**-P23565	Alpha-internexin	**Ina**	↓2.0	<0.0001	Cytoplasm, cytoskeleton	Cytoskeleton organization, developmental protein
**26**-Q64542	Plasma membrane calcium-transporting ATPase 4	**Atp2b4**	↓2.0	0.0289	Cell membrane	Calcium transport, hippocampus development
**27**-P30009	Myristoylated alanine-rich C-kinase substrate	**Marcks**	↓2.0	0.0029	Cytoplasm, cytoskeleton, cell membrane	Actin filament organization, brain development
**28**-D4A9L2	Serine/arginine-rich splicing factor 1	**Srsf1**	↓2.0	0.0201	Cytoplasm, nucleus	RNA processing

HIPPOCAMPUS-LYS744 – 3 mg/kg, 7 days

UP-regulated

**1**-E9PTB2	Transcription elongation factor SPT5	**Supt5h**	↑4.5	0.0036	Nucleus	Regulation of transcription
**2**-Q71UF4	Histone-binding protein RBBP7	**Rbbp7**	↑4.4	0.0404	Nucleus	Regulation of transcription
**3**-A0A096MIX2	RNA helicase	**Ddx17**	↑3.9	0.0067	Cytoplasm, nucleus	RNA processing
**4**-Q6MG61	Chloride intracellular channel protein 1	**Clic1**	↑3.2	0.0372	Cell membrane, cytoplasm, nucleus	Ion transport
**5**-Q9JM53	Apoptosis-inducing factor 1, mitochondrial	**Aifm1**	↑3.1	0.0187	Cytoplasm, mitochondrion, nucleus	Apoptosis
**6**-M0R7B4	H1.3 linker histone, cluster member	**Hist1h1d**	↑2.7	0.0002	Nucleus	DNA processing, nucleus assembly
**7**-Q00566	Methyl-CpG-binding protein 2	**Mecp2**	↑2.6	0.0134	Nucleus	RNA processing

**8**-Q6UPE1	Electron transfer flavoprotein-ubiquinone oxidoreductase, mitochondrial	**Etfdh**	↑2.6	0.0016	Mitochondrion	Electron transport chain
**9**-Q03344	ATPase inhibitor, mitochondrial	**Atpif1**	↑2.4	0.0037	Mitochondrion	Regulation of ATP metabolic process
**10**-Q62745	CD81 antigen	**Cd81**	↑2.4	0.0274	Cell membrane	Immunity
**11**-D3ZZ21	Complex I-B17	**Ndufb6**	↑2.4	0.0003	Mitochondrion	Respiratory electron transport
**12**-P00406	Cytochrome c oxidase subunit 2	**Mtco2**	↑2.3	0.0008	Mitochondrion	Respiratory electron transport
**13**-P35559	Insulin-degrading enzyme	**Ide**	↑2.2	0.0040	Cell membrane, cytoplasm, secreted	Insulin metabolism, proteolysis
**14**-Q00959	Glutamate receptor ionotropic, NMDA 2A	**Grin2a**	↑2.1	0.0156	Cell membrane, synapse	Ion transport, brain development
**15**-Q63881	Potassium voltage-gated channel subfamily D member 2	**Kcnd2**	↑2.1	0.0054	Cell membrane, synapse	Ion transport
**16**-D4ADE5	Histone-lysine N-methyltransferase SETD7	**Setd7**	↑2.1	0.0092	Nucleus	Regulation of transcription
**17**-P21396	Amine oxidase [flavin-containing] A	**Maoa**	↑2.0	0.0109	Mitochondrion	Catecholamine metabolism
**18**-P01015	Angiotensinogen	**Agt**	↑2.0	0.0462	Secreted	Vasoconstrictor, aging
**19**-Q63345	Myelin-oligodendrocyte glycoprotein	**Mog**	↑2.0	<0.0001	Cell membrane	Cell adhesion, aging
**20**-P07722	Myelin-associated glycoprotein	**Mag**	↑2.0	0.0005	Cell membrane	Cell adhesion, axon regeneration
**21**-Q4V7A0	WD repeat-containing protein 61	**Wdr61**	↑2.0	0.0219	Cytoplasm, nucleus	Regulation of transcription
**22**-P25886	60S ribosomal protein L29	**Rpl29**	↑2.0	0.0083	Ribosome, synapse	Aging, translation
**23**-Q99P82	Claudin-11	**Cldn11**	↑2.0	0.0029	Cell membrane	Cell adhesion
**24**-Q07647	Solute carrier family 2, facilitated glucose transporter member 3	**Slc2a3**	↑2.0	0.0268	Cell membrane	Glucose transport
Down-regulated						
**1**-C0JPT7	Filamin A	**Flna**	↓5.5	0.0115	Cell membrane, cytoplasm, nucleus, GA	Actin cytoskeleton organization
**2**-P16884	Neurofilament heavy polypeptide	**Nefh**	↓4.2	0.0004	Cytoplasm, cytoskeleton	Brain development, cytoskeleton organization
**3**-Q91XQ4	DNA-directed RNA polymerase II subunit GRINL1A	**Polr2m**	↓3.3	0.0048	Nucleus	Regulation of transcription, signal transduction
**4**-Q6AYJ8	Tumor necrosis factor alpha-induced protein 8-like protein 2	**Tnfaip8l2**	↓2.9	0.0488	Cytoplasm	Immunity, apoptosis
**5**-P02625	Parvalbumin alpha	**Pvalb**	↓2.7	0.0007	Cytoplasm, nucleus	Calcium-binding protein
**6**-P24594	Insulin-like growth factor-binding protein 5	**Igfbp5**	↓2.7	0.0305	Secreted	Aging, signal transduction
**7**-P63055	Purkinje cell protein 4	**Pcp4**	↓2.5	<0.0001	Cytoplasm, cytoskeleton	Signal transduction, apoptosis
**8**-D3ZRG7	RNA-binding motif protein 26	**Rbm26**	↓2.4	0.0089	Nucleus	RNA processing
**9**-F1LMV6	Desmoplakin	**Dsp**	↓2.4	<0.0001	Cytoplasm, cytoskeleton	Desmosome organization, cell-cell adhesion
**10**-B5DES0	Small nuclear ribonucleoprotein Sm D2	**Snrpd2**	↓2.3	0.0023	Cytoplasm, nucleus	RNA processing
**11**-Q5FVI4	Cell cycle exit and neuronal differentiation protein 1	**Cend1**	↓2.2	0.0036	Cell membrane	Neuronal differentiation
**12**-Q62876	Synaptogyrin-1	**Syngr1**	↓2.2	0.0054	Melanosome	Exocytosis
**13**-Q6P0K8	Junction plakoglobin	**Jup**	↓2.1	<0.0001	Cytoplasm, cytoskeleton, cell junction	Cell-cell adhesion, desmosome assembly

**14**-Q63910	Alpha globin	**Hba-a3**	↓2.1	0.0079	Cytoplasm, secreted	Oxygen transport
**15**-P06302	Prothymosin alpha	**Ptma**	↓2.1	0.0483	Nucleus	Cell differentiation, apoptosis
**16**-P21818	Stathmin-2	**Stmn2**	↓2.0	0.0053	Cytoplasm, endosome, GA	Regulator of microtubule stability
**17**-Q9EPJ0	Nuclear ubiquitous casein and cyclin-dependent kinase substrate 1	**Nucks1**	↓2.0	<0.0001	Nucleus	DNA processing
**18**-Q5M9G1	Protein HEXIM1	**Hexim1**	↓2.0	0.0009	Cytoplasm, nucleus	Immunity, regulation of transcription

*GA – Golgi apparatus

**ER – Endoplasmic reticulum
