# Supplementary material for: The Dose-Dependent Effects of Multifunctional Enkephalin Analogs on the Protein Composition of Rat Spleen Lymphocytes, Cortex, and Hippocampus; Comparison with Changes Induced by Morphine
Source: Biomedicines. 2022 Aug 14;10(8):1969. doi: 10.3390/biomedicines10081969 (PMC9406115; doi:10.3390/biomedicines10081969)
Supplement: Supplementary file 1 [file biomedicines-10-01969-s001.zip › Table S2.pdf]

Table S2:

a) Subcellular localization and function of altered proteins isolated from **rat spleen lymphocytes** after 7-day treatment with **morphine**, **LYS739** and **LYS744** (***10 mg/kg***) identified by label-free quantification (MaxLFQ).

Accession number	Protein name	Gene	Change (fold)	p value	Subcellular localization	Molecular functions and biological processes- keywords
**Lymphocytes -MORPHINE - 10 mg/kg, 7 days**						
UP-regulated						
**1**-P18590	Interferon-induced GTP-binding protein Mx3	**Mx3**	↑69.0	0.0019	Cytoplasm	Membrane fusion, receptor internalization
**2**-D4A678	Spectrin, alpha, erythrocytic 1	**Spta1**	↑13.7	0.0008	Cytoplasm, cytoskeleton	Actin cytoskeleton organization, homeostasis
**3**-Q05764	Beta-adducin	**Add2**	↑7.2	0.0023	Cell membrane, cytoskeleton	Actin filament bundle assembly
**4**-Q6AYE7	Interferon-induced protein with tetratricopeptide repeats 3	**Ifit3**	↑4.9	0.0002	Cytoplasm, mitochondrion	Regulation of cell proliferation, apoptosis
**5**-D4A559	Dematin actin-binding protein	**Dmtn**	↑3.8	0.0013	Cell membrane, cytoplasm	Actin cytoskeleton organization
**6**-A0A0G2K320	Sialic acid-binding Ig-like lectin 1	**Siglec1**	↑3.8	0.0085	Cell membrane, endosome	Endocytosis, apoptosis
**7**-F1M0R1	Ring finger protein 213	**Rnf213**	↑3.5	0.0281	Cytoplasm, nucleus	Protein ubiquitination
**8**-D3Z9Z0	Ankyrin-1	**Ank1**	↑3.5	0.0002	Cytoskeleton, ER*, nucleus	Vesicle-mediated transport
**9**-Q4V8H9	Interferon-induced protein with tetratricopeptide repeats 2	**Ifit2**	↑3.4	0.0128	Cytoplasm, ER	Regulation of apoptosis
**10**-G3V7N4	Tyrosine-protein kinase	**Syk**	↑3.0	0.0211	Cytoplasm, nucleus, cell membrane	Immunity, signal transduction
**11**-O08873	MAP kinase-activating death domain protein	**Madd**	↑2.8	0.0221	Cell membrane, cytoplasm	Apoptosis, synaptic vesicle transport
**12**-B5DF57	Protein-glutamine gamma-glutamyltransferase	**Epb42**	↑2.5	0.0009	Cell membrane, cytoplasm	Spleen development, ion homeostasis
**13**-F8WFT7	Anion exchange protein	**Slc4a1**	↑2.5	<0.0001	Cell membrane	Ion transport, anion exchange
**14**-Q5U2Z5	Cap-specific mRNA (nucleoside-2-O-)-methyltransferase 1	**Cmtr1**	↑2.4	0.0414	Nucleus	RNA processing
**15**-D4A929	WD repeat-containing protein 81	**Wdr81**	↑2.4	0.0226	Cytoplasm, endosome, lysosome	Protein stabilization, transport
**16**-P85845	Fascin	**Fscn1**	↑2.4	<0.0001	Cytoplasm, cytoskeleton	Actin cytoskeleton organization
**17**-G3V6W5	RNA helicase	**Ifih1**	↑2.4	0.0008	Cytoplasm	Immunity
**18**-Q63910	Alpha globin	**Hba-a3**	↑2.3	0.0002	Cytoplasm, secreted	Oxygen transport
**19**-P47967	Galectin-5	**Lgals5**	↑2.3	0.0008	Cytoplasm, nucleus	Cell proliferation, apoptosis
**20**-Q5U2R8	Interferon activated gene 204	**Mnda**	↑2.2	0.0019	Nucleus	Immunity, apoptosis
**21**-D4A3X3	ISG15 ubiquitin-like modifier	**Isg15**	↑2.2	0.0329	Cytoplasm, nucleus, secreted	ISG15 antiviral mechanism, protein ubiquitination
**22**-O09178	AMP deaminase 3	**Ampd3**	↑2.2	0.0167	Cytoplasm	Nucleotide metabolism
**23**-P14942	Glutathione S-transferase alpha-4	**Gsta4**	↑2.2	0.0054	Cytoplasm	Glutathione metabolism

**24**-Q99JC6	Tap-binding protein	**Tapbp**	↑2.1	0.0085	ER, GA**	Antigen processing, regulation of gene expression
**25**-Q5S255	Tyrosine-protein kinase	**Btk**	↑2.1	0.0102	Cytoplasm, nucleus	Immunity, signal transduction
**26**-G3V915	Ribonuclease L	**Rnasel**	↑2.1	0.0328	Nucleus	RNA processing
**27**-Q5M883	Chloride intracellular channel protein 2	**Clic2**	↑2.1	0.0260	Cytoplasm, cell membrane	Ion transport, ion homeostasis
**28**-M0R8V0	UHRF1-binding protein 1-like	**Uhrf1bp1l**	↑2.0	0.0098	Cytoplasm, endosome	Protein homodimerization activity
**29**-A0A0G2JVW6	Sterile alpha motif domain-containing 9	**Samd9**	↑2.0	0.0071	Cytoplasm, endosome	Spleen development, endosomal vesicle fusion
Down-regulated						
**1**-P02688	Myelin basic protein	**Mbp**	↓26.0	0.0037	Cell membrane	Aging, myelination
**2**-F1M0Z1	Triple functional domain protein	**Trio**	↓7.4	0.0082	Cytoplasm	Regulation of cell differentiation, axon guidance
**3**-D3ZIE4	FYN-binding protein 1	**Fyb1**	↓4.4	0.0151	Cytoplasm, nucleus	Immunity, adapter protein
**4**-Q05175	Brain acid soluble protein 1	**Basp1**	↓4.4	0.0307	Cell membrane	Calmodulin binding, regulation of transcription
**5**-Q91Z79	Liprin-alpha-3	**Ppfia3**	↓3.2	0.0300	Cytoplasm	Exocytosis, neurotransmitter secretion
**6**-P51650	Succinate-semialdehyde dehydrogenase, mitochondrial	**Aldh5a1**	↓3.1	0.0277	Mitochondrion	Glutamate metabolism, succinate metabolism
**7**-Q3ZBA0	Tectonin beta-propeller repeat-containing protein 1	**Tecpr1**	↓2.9	0.0327	Lysosome	Autophagy
**8**-D3ZVW3	Zinc finger CCCH-type-containing 4	**Zc3h4**	↓2.3	0.0495	Cytoplasm, nucleus	Metal ion binding
**9**-A8QJL8	Myc-associated zinc finger protein	**Maz**	↓2.2	0.0203	Cytoplasm, nucleus	Apoptosis, regulation of transcription
**10**-D4A4Z9	Kinectin 1	**Ktn1**	↓2.2	0.0115	ER	Protein transport
**11**-B4F7C2	Tubulin beta chain	**Tubb4a**	↓2.1	0.0151	Cytoplasm, cytoskeleton	Cytoskeleton organization
**12**-P20761	Ig gamma-2B chain C region	**Igh-1a**	↓2.1	0.0004	Cell membrane, secreted	Immunity
**13**-P35763	Perforin-1	**Prf1**	↓2.0	<0.0001	Cell membrane, endosome, lysosome	Apoptosis, immunity
**14**-Q00566	Methyl-CpG-binding protein 2	**Mecp2**	↓2.0	0.0069	Nucleus	Regulation of transcription, brain development
**15**-Q66HG8	Protein Red	**Ik**	↓2.0	0.0001	Cytoplasm, cytoskeleton, nucleus	RNA processing
**16**-M0R9Z5	Interferon regulatory factor 2-binding protein 2	**Irf2bp2**	↓2.0	<0.0001	Nucleus	Regulation of transcription

Lymphocytes -LYS739 - 10 mg/kg, 7 days

UP-regulated						
**1**-D3ZES7	Plexin A4	**Plxna4**	↑32.6	0.0291	Cell membrane	Regulation of axonogenesis, axon guidance
**2**-P20762	Ig gamma-2C chain C region	***N/A***	↑27.5	0.0003	Secreted	Immunity
**3**-P54100	Proto-oncogene vav	**Vav1**	↑9.5	0.0107	Cell-cell junction	Immunity, cell development
**4**-Q63041	Alpha-1-macroglobulin	**A1m**	↑8.1	0.0010	Secreted	Protease inhibitor

**5**-P14046	Alpha-1-inhibitor 3	**A1i3**	↑7.9	<0.0001	Secreted	Protease inhibitor
**6**-A0A096P6L8	Fibronectin	**Fn1**	↑4.5	0.0130	Cell membrane, secreted	Cell adhesion, cell shape
**7**-D4A8D5	Filamin B	**Flnb**	↑4.2	0.0006	Cytoplasm, nucleus	Actin cytoskeleton organization
**8**-Q5S255	Tyrosine-protein kinase	**Btk**	↑3.4	0.0002	Cytoplasm, nucleus	Immunity, signal transduction
**9**-G3V928	Prolow-density lipoprotein receptor-related protein 1	**Lrpi**	↑3.4	0.0027	Cell membrane, cytoplasm, GA, nucleus	Endocytosis, developmental protein
**10**-P0DMW1	Heat shock 70 kDa protein 1B	**Hspa1a**	↑3.1	0.0024	Cytoplasm, cytoskeleton	Chaperone, stress response
**11**-Q05764	Beta-adducin	**Add2**	↑3.1	0.0154	Cell membrane, cytoskeleton	Actin filament bundle assembly
**12**-Q9QX71	Napsin	**Napsa**	↑3.1	0.0007	Lysosome, secreted	Proteolysis
**13**-D3ZJ01	RAB-11 binding and LisH domain, coiled-coil and HEAT repeat-containing	**Relch**	↑2.9	0.0053	GA, endosome	Cholesterol transport
**14**-F1M9C0	Non-specific serine/threonine protein kinase	**Mapkapk2**	↑2.9	0.0139	Cytoplasm, nucleus	Signal transduction, inflammatory response
**15**-Q5U2R8	Interferon activated gene 204	**Mnda**	↑2.8	0.0022	Nucleus	Immunity, apoptosis
**16**-P24135	1-phosphatidylinositol 4,5-bisphosphate phosphodiesterase gamma-2	**Plcg2**	↑2.8	<0.0001	Cytoplasm, cell membrane	Signal transduction, lipid metabolism
**17**-Q62698	Cytoplasmic dynein 1 light intermediate chain 2	**Dync1li2**	↑2.7	0.0068	Cytoplasm, cytoskeleton	Motor protein, microtubule cytoskeleton organization
**18**-P04904	Glutathione S-transferase alpha-3	**Gsta3**	↑2.6	0.0063	Cytoplasm	Lipid metabolism, aging
**19**-Q4FZV0	Beta-mannosidase	**Manba**	↑2.6	0.0054	Lysosome	Carbohydrate metabolism
**20**-Q63448	Peroxisomal acyl-coenzyme A oxidase 3	**Acox3**	↑2.5	0.0034	Peroxisome	Lipid metabolism
**21**-D4A678	Spectrin, alpha, erythrocytic 1	**Spta1**	↑2.5	0.0048	Cytoplasm, cytoskeleton	Actin cytoskeleton organization, homeostasis
**22**-F1LZG6	LPS-responsive beige-like anchor protein	**Lrba**	↑2.5	0.0005	Cytoplasm, GA, ER, lysosome	Protein localization
**23**-P08430	UDP-glucuronosyltransferase 1-6	**Ugt1a6**	↑2.5	0.0341	ER	Xenobiotic metabolism
**24**-Q9WV57	Macrophage-expressed gene 1 protein	**Mpeg1**	↑2.4	0.0099	Cell membrane, cytoplasmic vesicle	Immunity
**25**-Q62753	Syntaxin-binding protein 2	**Stxbp2**	↑2.4	0.0043	Cytoplasm, cell membrane, lysosome	Exocytosis, protein transport
**26**-P11662	NADH-ubiquinone oxidoreductase chain 2	**Mtnd2**	↑2.4	0.0112	Mitochondrion	Respiratory electron transport
**27**-A0A0G2JUG7	IQ motif and SEC7 domain-containing protein 1	**Iqsec1**	↑2.3	0.0062	Cell junction, cytoplasm, nucleus	Neuronal development
**28**-Q99JC6	Tap-binding protein	**Tapbp**	↑2.3	0.0030	ER, GA	Antigen processing, regulation of gene expression
**29**-F1LSN8	Natural resistance-associated macrophage protein 1	**Slc11a1**	↑2.3	0.0012	Cell membrane, endosome, lysosome	Ion transport
**30**-Q920P0	L-xylulose reductase	**Dcxr**	↑2.3	0.0006	Cell membrane	Carbohydrate metabolism
**31**-Q9ESN0	Protein Niban	**Fam129a**	↑2.3	0.0002	Cytoplasm, cell membrane	Stress response, regulation of translation
**32**-Q5XI42	Aldehyde dehydrogenase family 3 member B1	**Aldh3b1**	↑2.3	0.0013	Cell membrane	Lipid metabolism
**33**-P25286	V-type proton ATPase 116 kDa subunit a1	**Atp6v0a1**	↑2.2	0.0282	Cell membrane, melanosome	Hydrogen ion transport
**34**-D3ZQ57	Plexin B2	**Plxnb2**	↑2.2	0.0013	Cell membrane	Brain development, regulation of cell shape
**35**-Q63745	Protein-tyrosine-phosphatase	**Ptpn12**	↑2.2	0.0301	Cytoplasm, nucleus	Protein dephosphorylation, tissue regeneration
**36**-B5DF45	TNF receptor-associated factor 6	**Traf6**	↑2.2	0.0022	Cytoplasm, nucleus	Protein ubiquitination, immunity
**37**-F1LSG8	Syndetin	**Vps50**	↑2.2	0.0121	Endosome, cell membrane	Protein transport, endocytic recycling
**38**-D4A7R3	Nucleoporin 205	**Nup205**	↑2.2	0.0309	Nucleus	Ion transport

**39**-D3Z8I4	Mitogen-activated protein kinase kinase kinase kinase 1	**Map4k1**	↑2.1	0.0029	Cell membrane	Signal transduction
**40**-Q498C8	Protein RER1	**Rer1**	↑2.1	0.0131	GA, cell membrane	Retrograde vesicle-mediated transport
**41**-Q5I0D5	Phospholysine phosphohistidine inorganic pyrophosphate phosphatase	**Lhpp**	↑2.1	0.0203	Cytoplasm, nucleus	Phosphate-containing compound metabolism
**42**-D3ZDR7	Thymocyte selection-associated family member 2	**Themis2**	↑2.1	0.0280	Cytoplasm, nucleus	Regulation of B cell activation
**43**-D3ZU55	Forkhead box K1	**Foxk1**	↑2.1	0.0115	Nucleus	Glucose metabolism
**44**-B5DEJ3	Alpha-mannosidase	**Man2b2**	↑2.1	0.0211	Lysosome	Mannose metabolism
**45**-Q641Z2	Tyrosine-protein phosphatase non-receptor type 9	**Ptpn9**	↑2.1	0.0143	Cytoplasm	Protein localization, protein phosphatase
**46**-P53669	LIM domain kinase 1	**Limk1**	↑2.0	0.0163	Cytoplasm, cytoskeleton, nucleus	Actin cytoskeleton organization
**47**-P85845	Fascin	**Fscn1**	↑2.0	0.0003	Cytoplasm, cytoskeleton	Actin cytoskeleton organization
**48**-Q6XLI7	Copine-1-like	**Rbm12**	↑2.0	0.0151	Nucleus	RNA processing
**49**-D4A3G2	Cyclin-dependent kinase 11B	**Cdk11b**	↑2.0	0.0013	Nucleus	Apoptosis
**50**-Q9R1T5	Aspartoacylase	**Aspa**	↑2.0	0.0154	Cytoplasm, nucleus	Aspartate and asparagine metabolism
**51**-B0BMT9	Sqrdl protein	**Sqor**	↑2.0	0.0191	Mitochondrion	Hydrogen sulfide metabolism
Down-regulated						
**1**-P47819	Glial fibrillary acidic protein	**Gfap**	↓8.7	0.0009	Cytoplasm, cytoskeleton	Intermediate filament organization, protein transport
**2**-Q811U3	ELKS/Rab6-interacting/CAST family member 1	**Erc1**	↓7.4	0.0132	Cytoplasm, GA, cell membrane	Protein transport, retrograde transport
**3**-Q9JJT1	Glucocorticoid-attenuated response gene 16 product	**Ifit1**	↓7.1	0.0045	Cytoplasm	Response to interferons, response to virus
**4**-Q91Z79	Liprin-alpha-3	**Ppfia3**	↓4.5	0.0037	Cytoplasm	Exocytosis, neurotransmitter secretion
**5**-Q6AXT7	RNA-binding protein 42	**Rbm42**	↓4.5	0.0024	Cytoplasm, nucleus	RNA processing
**6**-Q62715	Neutrophil antibiotic peptide NP-2	**Defa**	↓4.2	0.0272	Secreted	Immunity
**7**-F1LMV6	Desmoplakin	**Dsp**	↓3.9	0.0301	Cytoplasm, cytoskeleton	Desmosome organization, cell-cell adhesion
**8**-P31016	Disks large homolog 4	**Dlg4**	↓3.8	0.0085	Cytoplasm, cell membrane	Protein folding, cell-cell adhesion
**9**-A1A5S2	CXXC-type zinc finger protein 1	**Cxxc1**	↓3.4	0.0028	Nucleus	Regulation of transcription
**10**-Q3B7T9	Rab11 family-interacting protein 1	**Rab11fip1**	↓3.4	0.0164	Endosome	Protein transport
**11**-Q05175	Brain acid soluble protein 1	**Basp1**	↓3.3	0.0225	Cell membrane	Regulation of transcription
**12**-P06687	Sodium/potassium-transporting ATPase subunit alpha-3	**Atp1a3**	↓3.3	0.0291	Cell membrane	Sodium/potassium transport
**13**-Q00566	Methyl-CpG-binding protein 2	**Mecp2**	↓3.3	0.0001	Nucleus	RNA processing
**14**-D3ZAY8	Pinin	**Pnn**	↓3.2	0.0298	Nucleus	RNA processing
**15**-Q63016	Large neutral amino acids transporter small subunit 1	**Slc7a5**	↓3.2	0.0015	Cell membrane, lysosome	Amino-acid transport
**16**-D3ZXP3	Histone H2A	**H2afx**	↓3.2	0.0005	Nucleus	DNA processing
**17**-F1M1R4	RNA-binding motif protein 27	**Rbm27**	↓3.1	0.0264	Nucleus	RNA processing
**18**-Q68FR7	Protein LTV1 homolog	**Ltv1**	↓3.0	0.0085	Cytoplasm, nucleus	RNA processing

**19**-D4A2D8	LSM7 homolog, U6 small nuclear RNA and mRNA degradation-associated	**Lsm7**	↓2.9	0.0002	Nucleus	RNA processing
**20**-D3ZBE8	UPF3B, regulator of nonsense mediated mRNA decay	**Upf3b**	↓2.8	0.0002	Cytoplasm, nucleus	RNA processing
**21**-Q8K585	High mobility group protein HMG-I/HMG-Y	**Hmga1**	↓2.8	0.0049	Nucleus	Regulation of transcription
**22**-P18437	Non-histone chromosomal protein HMG-17	**Hmgn2**	↓2.8	0.0006	Cytoplasm, nucleus	Regulation of transcription, chromation organization
**23**-Q62733	Lamina-associated polypeptide 2, isoform beta	**Tmpo**	↓2.7	0.0001	Nucleus	Regulation of transcription
**24**-B2GV37	Zinc finger protein 593	**Zfp593**	↓2.6	0.0012	Nucleus	Regulation of transcription
**25**-A8QJL8	Myc-associated zinc finger protein	**Maz**	↓2.6	0.0103	Cytoplasm, nucleus	Apoptosis, regulation of transcription
**26**-Q6TQE1	Zinc finger CCCH domain-containing protein 18	**Zc3h18**	↓2.5	0.0006	Nucleus	RNA processing
**27**-Q4QQU6	Survival of motor neuron-related-splicing factor 30	**Smndc1**	↓2.5	0.0021	Nucleus	RNA processing, apoptosis
**28**-F1LWZ8	LEM domain-containing 3	**Lemd3**	↓2.5	0.0007	Nucleus, cell membrane	Nucleus organization, cell cycle
**29**-Q5U2N1	Negative elongation factor complex member A	**Nelfa**	↓2.5	0.0004	Cytoplasm, nucleus	Regulation of transcription
**30**-Q66HG8	Protein Red	**Ik**	↓2.5	0.0001	Cytoplasm, cytoskeleton, nucleus	RNA processing
**31**-D3ZSM5	Sp2 transcription factor	**Sp2**	↓2.4	0.0082	Nucleus	Regulation of transcription
**32**-Q642C0	DnaJ homolog subfamily C member 8	**Dnajc8**	↓2.4	0.0030	Nucleus	Chaperone, RNA processing
**33**-Q5XIW8	U4/U6.U5 tri-snRNP-associated protein 1	**Sart1**	↓2.4	0.0003	Nucleus	RNA processing
**34**-A0A0G2JW88	Microtubule-associated protein	**Map4**	↓2.4	0.0003	Cytoplasm, cytoskeleton	Microtubule cytoskeleton organization
**35**-P02262	Histone H2A type 1	***N/A***	↓2.4	<0.0001	Nucleus	DNA processing
**36**-Q8K3X0	Protein CASC3	**Casc3**	↓2.4	0.0089	Cytoplasm, nucleus	RNA processing
**37**-Q5XJW2	Growth arrest and DNA damage-inducible proteins-interacting protein 1	**Gadd45gip1**	↓2.4	0.0094	Mitochondrion, nucleus	Cell cycle, mitochondrial protein synthesis
**38**-D3ZY40	PCF11 cleavage and polyadenylation factor subunit	**Pcf11**	↓2.3	0.0002	Cytoplasm, nucleus, mitochondrion	RNA processing
**39**-D3ZWF5	Transcription and mRNA export factor ENY2	**Eny2**	↓2.3	<0.0001	Nucleus	RNA processing, protein transport
**40**-A0A0G2K4R1	Protein phosphatase 1 regulatory subunit	**Ppp1r12c**	↓2.3	0.0281	Cytoplasm	Signal transduction
**41**-P62329	Thymosin beta-4	**Tmsb4x**	↓2.3	0.0013	Cytoplasm, cytoskeleton	Actin cytoskeleton organization
**42**-Q5RKG9	Eukaryotic translation initiation factro 4B	**Eif4b**	↓2.3	0.0012	Cytoplasm	Translation initiation factor activity
**43**-Q5PQQ2	WW domain-binding protein 11	**Wbp11**	↓2.3	0.0004	Cytoplasm, nucleus	RNA processing
**44**-D4A0Y6	Marker of proliferation Ki-67	**Mki67**	↓2.3	0.0013	Nucleus	Cell cycle
**45**-F1LRS8	CD2-associated protein	**Cd2ap**	↓2.3	0.0029	Cytoplasm, cytoskeleton	Actin cytoskeleton organization, cell cycle
**46**-Q99P99	Histone deacetylase 4	**Hdac4**	↓2.3	0.0249	Cytoplasm, nucleus	Regulation of transcription, histone deacetylation
**47**-M0R991	Natural killer cell triggering receptor	**Nktr**	↓2.2	0.0045	Cytoplasm, nucleus	Protein folding
**48**-Q5BJP2	Spliceosome-associated protein CWC15 homolog	**Cwc15**	↓2.2	0.0008	Nucleus	RNA processing
**49**-P50554	4-aminobutyrate aminotransferase, mitochondrial	**Abat**	↓2.2	0.0108	Mitochondrion	Neurotransmitter degradation, aging
**50**-D3ZC98	Cyclin-T1	**Ccnt1**	↓2.2	0.0017	Nucleus	Regulation of transcription
**51**-Q00715	Histone H2B type 1	***N/A***	↓2.2	0.0017	Nucleus	DNA processing
**52**-Q9EPJ0	Nuclear ubiquitous casein and cyclin-dependent kinase substrate 1	**Nucks1**	↓2.1	0.0121	Nucleus	DNA processing

**53**-B2RZ79	Iron-sulfur cluster assembly enzyme	**Iscu**	↓2.1	0.0106	Cytoplasm, mitochondrion	Iron ion homeostasis
**54**-D3ZSC8	DnaJ homolog subfamily C member 17	**Dnajc17**	↓2.1	0.0123	Cytoplasm, nucleus	Chaperone, regulation of transcription
**55**-P41777	Nucleolar and coiled-body phosphoprotein 1	**Nolc1**	↓2.1	0.0076	Cytoplasm, nucleus	Regulation of translation, nucleolus organization
**56**-P18291	Granzyme B	**Gzmb**	↓2.1	<0.0001	Lysosome, secreted	Apoptosis
**57**-B2GV14	Taxilin alpha	**Txlna**	↓2.1	0.0039	Cytoplasm	B cell activation
**58**-Q0PXQ8	Interferon regulatory factor	**Irf2**	↓2.1	0.0027	Nucleus	Regulation of transcription, immunity
**59**-Q1RP77	Nucleolar protein 16	**Nop16**	↓2.1	0.0044	Nucleus	Ribosomal large subunit biogenesis
**60**-Q4FZU3	Nuclear speckle splicing regulatory protein 1	**Nsrp1**	↓2.1	0.0050	Nucleus	RNA processing
**61**-P69736	Endothelial differentiation-related factor 1	**Edf1**	↓2.0	0.0041	Cytoplasm, nucleus	Regulation of transcription, cell differentiation
**62**-D4ACZ2	Paired box 2	**Pax2**	↓2.0	0.0264	Nucleus	Regulation of transcription, apoptosis, aging
**63**-Q5I0H9	Protein disulfide-isomerase A5	**Pdia5**	↓2.0	0.0028	ER	Protein folding, isomerase
**64**-Q63014	A-kinase anchor protein 8	**Akap8**	↓2.0	0.0194	Cytoplasm, nucleus	Immunity, regulation of transcription, transport
**65**-Q5HZF2	WW domain-binding protein 4	**Wbp4**	↓2.0	0.0002	Nucleus	RNA processing
**66**-D3ZDP2	Mitochondrial ribosomal protein L58	**Mrpl58**	↓2.0	0.0014	Mitochondrion	Regulation of translation
**67**-D4ABH1	SR-related CTD-associated factor 11	**Scaf11**	↓2.0	0.0013	Nucleus	RNA processing
**68**-D3ZR17	U4/U6.U5 small nuclear ribonucleoprotein 27 kDa protein	**Snrnp27**	↓2.0	0.0002	Nucleus	RNA processing

Lymphocytes -LYS744 - 10 mg/kg, 7 days

UP-regulated

**1**-O35543	Hematopoietic prostaglandin D synthase	**Hpgds**	↑9.7	0.0099	Cytoplasm	Lipid metabolism, prostaglandin metabolism
**2**-D4A678	Spectrin, alpha, erythrocytic 1	**Spta1**	↑9.1	0.0002	Cytoplasm, cytoskeleton	Actin cytoskeleton organization, homeostasis
**3**-Q05764	Beta-adducin	**Add2**	↑8.2	<0.0001	Cell membrane, cytoskeleton	Actin filament bundle assembly
**4**-Q63041	Alpha-1-macroglobulin	**A1m**	↑6.3	0.0016	Secreted	Protease inhibitor
**5**-P14046	Alpha-1-inhibitor 3	**A1i3**	↑4.2	0.0003	Secreted	Protease inhibitor
**6**-Q1AAU6	Arf-GAP with SH3 domain, ANK repeat and PH domain-containing protein 1	**Asap1**	↑4.1	0.0300	Cell membrane, cytoplasm	Signal transduction, regulation of GTPase activity
**7**-A0A096P6L8	Fibronectin	**Fn1**	↑4.0	0.0073	Cell membrane, secreted	Cell adhesion, cell shape
**8**-D4AEH9	4-alpha-glucanotransferase	**Agl**	↑3.7	0.0002	Cytoplasm	Glycogen metabolism
**9**-P54100	Proto-oncogene vav	**Vav1**	↑3.7	0.0473	Cell-cell junction	Immunity, cell development
**10**-D4A8D5	Filamin B	**Flnb**	↑3.5	0.0004	Cytoplasm, nucleus	Actin cytoskeleton organization
**11**-Q5U2R8	Interferon activated gene 204	**Mnda**	↑3.4	0.0002	Nucleus	Immunity, apoptosis
**12**-B5DF29	Replication factor C (Activator 1) 5	**Rfc5**	↑3.4	0.0049	Nucleus	DNA processing
**13**-D3Z9Z0	Ankyrin-1	**Ank1**	↑3.4	<0.0001	Cytoskeleton, ER**, nucleus	Vesicle-mediated transport

**14**-D4A929	WD repeat-containing protein 81	**Wdr81**	↑3.3	0.0077	Cytoplasm, endosome, lysosome	Protein stabilization, transport
**15**-D4A7R3	Nucleoporin 205	**Nup205**	↑3.1	0.0143	Nucleus	Ion transport
**16**-Q5M830	Replication factor C (Activator 1) 3	**Rfc3**	↑3.0	0.0141	Nucleus	DNA processing
**17**-G3V928	Prolow-density lipoprotein receptor-related protein 1	**Lrpi**	↑2.9	0.0110	Cell membrane, cytoplasm, GA, nucleus	Endocytosis, developmental protein
**18**-P08430	UDP-glucuronosyltransferase 1-6	**Ugt1a6**	↑2.8	0.0085	ER	Xenobiotic metabolism
**19**-A0A096MKG2	Nucleolar protein 6	**Nol6**	↑2.8	0.0056	Nucleus	RNA processing
**20**-D3ZKG7	Inositol polyphosphate-5-phosphatase F	**Inpp5f**	↑2.7	0.0150	Endosome, cell membrane	Phosphatidylinositol metabolism
**21**-F1LZG6	LPS-responsive beige-like anchor protein	**Lrba**	↑2.7	0.0001	Cytoplasm, GA, ER, lysosome	Protein localization
**22**-B5DFK0	Leng4 protein	**Mboat7**	↑2.7	0.0010	Cell membrane	Phosphatidylinositol metabolism
**23**-P85845	Fascin	**Fscn1**	↑2.7	<0.0001	Cytoplasm, cytoskeleton	Actin cytoskeleton organization
**24**-Q63448	Peroxisomal acyl-coenzyme A oxidase 3	**Acox3**	↑2.7	0.0222	Peroxisome	Lipid metabolism
**25**-A0A140UHX6	Spectrin beta chain	**Sptb**	↑2.7	0.0009	Cytoplasm, cytoskeleton	Actin capping
**26**-P25286	V-type proton ATPase 116 kDa subunit a1	**Atp6v0a1**	↑2.6	0.0052	Cell membrane, melanosome	Hydrogen ion transport
**27**-G3V9Q4	Serine/threonine kinase 38	**Stk38**	↑2.6	0.0071	Cytoplasm, nucleus	Signal transduction
**28**-D4ACK1	Nucleoporin 214	**Nup214**	↑2.6	0.0226	Nucleus	Regulation of cell cycle
**29**-Q9WV57	Macrophage-expressed gene 1 protein	**Mpeg1**	↑2.6	0.0070	Cell membrane, cytoplasmic vesicle	Immunity
**30**-F1LXT3	UTP20 small subunit processome component	**Utp20**	↑2.6	0.0115	Nucleus, cell membrane	RNA processing
**31**-B5DF57	Protein-glutamine gamma-glutamyltransferase	**Epb42**	↑2.6	0.0007	Cell membrane, cytoplasm	Spleen development, ion homeostasis
**32**-F1LSG8	Syndetin	**Vps50**	↑2.5	0.0026	Endosome, cell membrane	Protein transport, endocytic recycling
**33**-Q63745	Protein-tyrosine-phosphatase	**Ptpn12**	↑2.5	0.0019	Cytoplasm, nucleus	Protein dephosphorylation, tissue regeneration
**34**-P53669	LIM domain kinase 1	**Limk1**	↑2.5	0.0014	Cytoplasm, cytoskeleton, nucleus	Actin cytoskeleton organization
**35**-Q5S255	Tyrosine-protein kinase	**Btk**	↑2.5	0.0007	Cytoplasm, nucleus	Immunity, signal transduction
**36**-P70712	Kynureninase	**Kynu**	↑2.5	0.0007	Cytoplasm	Tryptophan metabolism
**37**-Q5M9F8	N-terminal kinase-like protein	**Scyl1**	↑2.5	0.0030	Cytoplasm, ER, GA	ER-Golgi transport
**38**-P22509	rRNA 2-O-methyltransferase fibrillarin	**Fbl**	↑2.5	<0.0001	Nucleus	RNA processing
**39**-P23606	Protein-glutamine gamma-glutamyltransferase K	**Tgm1**	↑2.5	0.0062	Cell membrane	Cell proliferation
**40**-F8WFT7	Anion exchange protein	**Slc4a1**	↑2.5	<0.0001	Cell membrane	Ion transport
**41**-D3ZYQ9	E3 ubiquitin protein ligase	**Rnf20**	↑2.4	0.0186	Nucleus	Protein ubiquitination, chromatin regulator
**42**-A0A096MJ28	Integrator complex subunit 2	**Ints2**	↑2.4	0.0455	Nucleus	RNA processing
**43**-P63081	V-type proton ATPase 16 kDa proteolipid subunit	**Atp6v0c**	↑2.4	0.0110	Cell membrane	Ion transport
**44**-D3ZBP4	Protein-methionine sulfoxide oxidase MICAL1	**Mical1**	↑2.4	0.0032	Cytoplasm, cytoskeleton	Actin filament depolymerization, apoptosis
**45**-Q63356	Unconventional myosin-Ie	**Myo1e**	↑2.4	0.0129	Cytoplasmic vesicle, cytoskeleton	Actin filament organization, endocytosis
**46**-A0A0G2JUG7	IQ motif and SEC7 domain-containing protein 1	**Iqsec1**	↑2.3	0.0363	Cell junction, cytoplasm, nucleus	Neuronal development
**47**-Q9QX71	Napsin	**Napsa**	↑2.3	0.0039	Lysosome, secreted	Proteolysis

**48**-Q5XI42	Aldehyde dehydrogenase family 3 member B1	**Aldh3b1**	↑2.3	0.0012	Cell membrane	Lipid metabolism
**49**-Q9R1N3	Sodium bicarbonate cotransporter 3	**Slc4a7**	↑2.3	0.0262	Cell membrane	Ion transport
**50**-P08413	Calcium/calmodulin-dependent protein kinase type II subunit beta	**Camk2b**	↑2.3	0.0256	Cytoplasm, cytoskeleton	Neurogenesis, actin cytoskeleton organization
**51**-B0BMT9	Sqrdl protein	**Sqor**	↑2.3	0.0004	Mitochondrion	Hydrogen sulfide metabolism
**52**-D3ZAQ4	Proteasome assembly chaperone 2	**Psmg2**	↑2.3	0.0263	Cytoplasm, nucleus	Chaperone, apoptosis
**53**-P14942	Glutathione S-transferase alpha-4	**Gsta4**	↑2.2	0.0048	Cytoplasm	Glutathione metabolism
**54**-D3Z8I4	Mitogen-activated protein kinase kinase kinase kinase 1	**Map4k1**	↑2.2	0.0005	Cell membrane	Signal transduction
**55**-P25977	Nucleolar transcription factor 1	**Ubtf**	↑2.2	0.0030	Nucleus	Regulation of transcription
**56**-G3V915	Ribonuclease L	**Rnasel**	↑2.2	0.0155	Nucleus	RNA processing
**57**-D3ZDR7	Thymocyte selection-associated family member 2	**Themis2**	↑2.2	0.0106	Cytoplasm, nucleus	Regulation of B cell activation
**58**-D4A980	MALT1 paracaspase	**Malt1**	↑2.2	0.0175	Cytoplasm, nucleus	Cell proliferation, apoptosis, immunity
**59**-P20762	Ig gamma-2C chain C region	***N/A***	↑2.2	0.0438	Secreted	Immunity
**60**-O09178	AMP deaminase 3	**Ampd3**	↑2.2	0.0135	Cytoplasm	Nucleotide metabolism
**61**-P97690	Structural maintenance of chromosomes protein 3	**Smc3**	↑2.2	0.0176	Nucleus	Cell cycle, DNA processing
**62**-P05508	NADH-ubiquinone oxidoreductase chain 4	**Mtnd4**	↑2.2	0.0007	Mitochondrion	Respiratory electron transport
**63**-Q920P0	L-xylulose reductase	**Dcxr**	↑2.2	0.0018	Cell membrane	Carbohydrate metabolism
**64**-A0A0G2JY08	Unconventional myosin-XVIIIa	**Myo18a**	↑2.2	<0.0001	Cytoplasm, GA	Motor protein, Golgi organization
**65**-D4A020	Mediator of RNA polymerase II transcription subunit 14	**Med14**	↑2.2	0.0211	Nucleus	Regulation of transcription
**66**-F1LM60	ArfGAP with RhoGAP domain, ankyrin repeat and PH domain 1	**Arap1**	↑2.2	0.0280	Cytoplasm	Signal transduction
**67**-Q8CHJ1	Phosphatidylinositol glycan anchor biosynthesis class U protein	**Pigu**	↑2.2	0.0345	ER	Protein localization, GPI-anchor biosynthesis
**68**-P50545	Tyrosine-protein kinase HCK	**Hck**	↑2.2	0.0006	Cytoplasm, cell membrane, lysosome, nucleus, GA	Cell differentiation, exocytosis, immunity, apoptosis
**69**-Q63355	Unconventional myosin-Ic	**Myo1c**	↑2.2	0.0365	Cell membrane, cytoplasm	Protein transport, motor protein
**70**-B2RYP8	Gamma-tubulin complex component	**Tubgcp2**	↑2.2	0.0395	Cytoplasm, cytoskeleton	Microtubule organization, brain development
**71**-F1LTT7	DENN-domain containing 4B	**Dennd4b**	↑2.2	0.0035	Cytoplasm, nucleus, GA	Signal transduction
**72**-Q6XLI7	Copine-1-like	**Rbm12**	↑2.2	0.0087	Nucleus	RNA processing
**73**-D3ZU51	Ribonuclease P/MRP subunit p30	**Rpp30**	↑2.1	0.0142	Nucleus	RNA processing
**74**-Q62753	Syntaxin-binding protein 2	**Stxbp2**	↑2.1	0.0456	Cytoplasm, cell membrane, lysosome	Exocytosis, protein transport
**75**-G3V9T0	Protein inhibitor of activated STAT 1 (Predicted)	**Pias1**	↑2.1	0.0011	Nucleus	Protein modification
**76**-D4ACL8	Serine-protein kinase ATM	**Atm**	↑2.1	0.0225	Cytoplasm, nucleus	Signal transduction, apoptosis, immunity
**77**-D3Z8V4	NCK associated protein 1 like (Predicted)	**Nckap1l**	↑2.1	0.0004	Cytoplasm	Cell homeostasis, apoptosis
**78**-P61621	Protein transport protein Sec61 subunit alpha isoform 1	**Sec61a1**	↑2.1	0.0178	ER	Protein transport, translocation
**79**-D3ZQ57	Plexin B2	**Plxnb2**	↑2.1	0.0026	Cell membrane	Brain development, regulation of cell shape
**80**-Q63189	Bone marrow proteoglycan	**Prg2**	↑2.0	0.0002	Cytoplasm	Immunity

**81**-A3KNA0	RNA helicase aquarius	**Aqr**	↑2.0	0.0006	Nucleus	RNA processing
**82**-P49187	Mitogen-activated protein kinase 10	**Mapk10**	↑2.0	0.0240	Cytoplasm, cell membrane, nucleus, mitochondrion	Signal transduction, biological rhythms
**83**-P51952	Cyclin-dependent kinase 7	**Cdk7**	↑2.0	0.0064	Cytoplasm, nucleus	Cell cycle, regulation of transcription
**84**-F1LQM8	DNA mismatch repair protein	**Msh3**	↑2.0	0.0026	Nucleus	DNA processing
**85**-Q8R3Z7	EH-domain-containing 4	**Edh4**	↑2.0	0.0042	Cell membrane, endosome	Endocytosis
**86**-P11505	Plasma membrane calcium-transporting ATPase 1	**Atp2b1**	↑2.0	0.0086	Cell membrane, cytoplasmic vesicle	Ion transport, aging
**87**-P03889	NADH-ubiquinone oxidoreductase chain 1	**Mtnd1**	↑2.0	0.0135	Mitochondrion	Respiratory electron transport
**88**-G3V660	NOL1/NOP2/Sun domain family, member 5 (Predicted)	**Nsun5**	↑2.0	0.0395	Nucleus	RNA processing
Down-regulated						
**1**-P06302	Prothymosin alpha	**Ptma**	↓9.7	0.0085	Nucleus	Cell differentiation, apoptosis
**2**-Q5BJT0	Arginine and glutamate-rich protein 1	**Arglu1**	↓8.7	0.0066	Cytoplasm, mitochondrion, nucleus	Regulation of transcription
**3**-D3ZVW3	Zinc finger CCCH-type-containing 4	**Zc3h4**	↓5.6	0.0025	Cytoplasm, nucleus	Metal ion binding
**4**-Q5EB96	Septin-1	**Sept1**	↓4.9	0.0067	Cytoplasm, cytoskeleton	Cell cycle, exocytosis
**5**-Q6AXT7	RNA-binding protein 42	**Rbm42**	↓4.4	0.0026	Cytoplasm, nucleus	RNA processing
**6**-P31016	Disks large homolog 4	**Dlg4**	↓4.2	0.0048	Cytoplasm, cell membrane	Protein folding, cell-cell adhesion
**7**-P06687	Sodium/potassium-transporting ATPase subunit alpha-3	**Atp1a3**	↓4.2	0.0170	Cell membrane	Sodium/potassium transport
**8**-D4A2B0	DNA polymerase delta-interacting protein 3	**Poldip3**	↓4.1	0.0008	Nucleus	Regulation of transcription
**9**-D3ZAY8	Pinin	**Pnn**	↓4.0	0.0488	Nucleus	RNA processing
**10**-D3ZC98	Cyclin-T1	**Ccnt1**	↓4.0	0.0004	Nucleus	Regulation of transcription
**11**-P18291	Granzyme B	**Gzmb**	↓3.9	<0.0001	Lysosome, secreted	Apoptosis
**12**-Q4V7G0	T-cell receptor T3 zeta chain	**Cd247**	↓3.4	<0.0001	Cell membrane	Protein localization
**13**-Q99P99	Histone deacetylase 4	**Hdac4**	↓3.3	0.0002	Cytoplasm, nucleus	Regulation of transcription, histone deacetylation
**14**-Q91Z79	Liprin-alpha-3	**Ppfia3**	↓3.3	0.0099	Cytoplasm	Exocytosis, neurotransmitter secretion
**15**-B2GV74	Kinesin light chain	**Klc2**	↓3.2	0.0459	Cytoplasm, cytoskeleton	Lysosome localization, motor protein
**16**-F1M1R4	RNA-binding motif protein 27	**Rbm27**	↓3.2	0.0246	Nucleus	RNA processing
**17**-A1A5S2	CXXC-type zinc finger protein 1	**Cxxc1**	↓3.1	0.0009	Nucleus	Regulation of transcription
**18**-D3ZHV1	RCG35421, isoform CRA_b	**Tnip1**	↓3.1	0.0003	Cytoplasm, nucleus	Cell-cell adhesion, inflammatory response
**19**-D4ADB4	CGG triplet repeat binding protein 1 (Predicted)	**Cggbp1**	↓3.0	0.0087	Nucleus	Regulation of transcription
**20**-Q62733	Lamina-associated polypeptide 2, isoform beta	**Tmpo**	↓3.0	0.0004	Nucleus	Regulation of transcription
**21**-Q05175	Brain acid soluble protein 1	**Basp1**	↓3.0	0.0317	Cell membrane	Regulation of transcription
**22**-G3V8S9	Cathelicidin antimicrobial peptide	**Camp**	↓3.0	0.0002	Secreted	Immunity

**23**-D4AD01	RCG49513	**Zmat2**	↓2.9	0.0017	Nucleus	RNA processing
**24**-A9UMW0	Ubiquitin-like protein 5	**Ubl5**	↓2.9	0.0440	Nucleus, cytoplasm	RNA processing
**25**-D3ZIE4	FYN-binding protein 1	**Fyb1**	↓2.8	0.0333	Cytoplasm, nucleus	Immunity
**26**-Q00566	Methyl-CpG-binding protein 2	**Mecp2**	↓2.8	0.0006	Nucleus	RNA processing
**27**-Q68FR7	Protein LTV1 homolog	**Ltv1**	↓2.8	0.0412	Cytoplasm, nucleus	RNA processing
**28**-B2GV37	Zinc finger protein 593	**Zfp593**	↓2.8	0.0003	Nucleus	Regulation of transcription
**29**-Q6PCT5	Polyglutamine-binding protein 1	**Pqbp1**	↓2.7	0.0150	Nucleus	Immunity, regulation of transcription
**30**-A0A0G2JW88	Microtubule-associated protein	**Map4**	↓2.7	<0.0001	Cytoplasm, cytoskeleton	Microtubule cytoskeleton organization
**31**-P35763	Perforin-1	**Prf1**	↓2.6	<0.0001	Cell membrane, endosome, lysosome	Apoptosis, immunity
**32**-Q62714	Neutrophil antibiotic peptide NP-4	**Np4**	↓2.6	0.0034	Secreted	Immunity
**33**-D3ZSC8	DnaJ homolog subfamily C member 17	**Dnajc17**	↓2.6	0.0013	Cytoplasm, nucleus	Chaperone, regulation of transcription
**34**-P62329	Thymosin beta-4	**Tmsb4x**	↓2.6	0.0004	Cytoplasm, cytoskeleton	Actin cytoskeleton organization
**35**-Q66HG8	Protein Red	**Ik**	↓2.5	<0.0001	Cytoplasm, cytoskeleton, nucleus	RNA processing
**36**-F1LWZ8	LEM domain-containing 3	**Lemd3**	↓2.5	0.0002	Nucleus, cell membrane	Nucleus organization, cell cycle
**37**-M0R991	Natural killer cell triggering receptor	**Nktr**	↓2.5	0.0063	Cytoplasm, nucleus	Protein folding
**38**-D4ABH1	SR-related CTD-associated factor 11	**Scaf11**	↓2.5	<0.0001	Nucleus	RNA processing
**39**-Q642C0	DnaJ homolog subfamily C member 8	**Dnajc8**	↓2.5	0.0011	Nucleus	Chaperone, RNA processing
**40**-D3ZFB2	LUC7-like 3 pre-mRNA-splicing factor	**Luc7l3**	↓2.5	0.0009	Nucleus	RNA processing
**41**-Q53UA7	Serine/threonine-protein kinase TAO3	**Taok3**	↓2.4	<0.0001	Cytoplasm	Signal transduction, DNA processing
**42**-D3ZUL1	Coiled-coil domain containing 124	**Ccdc124**	↓2.4	0.0005	Nucleus	Regulation of transcription
**43**-P50411	Protein phosphatase inhibitor 2	**Ppp1r2**	↓2.4	0.0332	Cytoplasm	Glycogen metabolism, protein phosphatase inhibitor
**44**-Q5XJW2	Growth arrest and DNA damage-inducible proteins-interacting protein 1	**Gadd45gip1**	↓2.4	0.0351	Mitochondrion, nucleus	Cell cycle, mitochondrial protein synthesis
**45**-P47971	Neuronal pentraxin-1	**Nptx1**	↓2.4	0.0235	Cytoplasmic vesicle	Axonogenesis involved in innervation
**46**-B0BMS9	PRKR-interacting protein 1	**Prkrip1**	↓2.4	0.0044	Nucleus	RNA processing
**47**-D4ADT5	DEAD (Asp-Glu-Ala-Asp) box polypeptide 58 (Predicted)	**Ddx58**	↓2.4	0.0118	Cytoplasm, cytoskeleton	Immunity, regulation of cell migration
**48**-D4A4T0	RING-type E3 ubiquitin transferase	**Stub1**	↓2.4	0.0155	Cytoplasm, ER, nucleus	Chaperone, protein ubiquitination
**49**-Q4FZU3	Nuclear speckle splicing regulatory protein 1	**Nsrp1**	↓2.4	0.0010	Nucleus	RNA processing
**50**-D3ZMR1	Mitochondrial import receptor subunit TOM7 homolog	**Tomm7**	↓2.3	0.0488	Mitochondrion	Protein transport
**51**-A8QJL8	Myc-associated zinc finger protein	**Maz**	↓2.3	0.0012	Cytoplasm, nucleus	Apoptosis, regulation of transcription
**52**-M0R9Z5	Interferon regulatory factor 2-binding protein 2	**Irf2bp2**	↓2.3	0.0002	Nucleus	Regulation of transcription
**53**-D3ZGY1	PYM homolog 1, exon junction complex-associated factor	**Pym1**	↓2.3	0.0164	Cytoplasm	Regulation of translation
**54**-Q8CJF4	Granzyme A	**Gzma**	↓2.3	0.0003	Nucleus	Apoptosis
**55**-Q5U2N1	Negative elongation factor complex member A	**Nelfa**	↓2.3	0.0083	Cytoplasm, nucleus	Regulation of transcription
**56**-D4AD33	RNA guanine-7 methyltransferase-activating subunit	**Ramac**	↓2.3	0.0237	Nucleus	RNA processing

**57**-M0RC54	Biorientation of chromosomes in cell division 1-like 1	**Bod1l1**	↓2.3	0.0433	Cytoplasm, cytoskeleton, nucleus	DNA processing
**58**-D3ZPQ3	XIAP-associated factor 1	**Xaf1**	↓2.3	0.0022	Nucleus	Apoptosis
**59**-Q5RKG9	Eukaryotic translation initiation factor 4B	**Eif4b**	↓2.3	0.0002	Cytoplasm	Translation initiation factor activity
**60**-Q792Q4	Cysteine-rich PDZ-binding protein	**Cript**	↓2.2	0.0124	Cytoplasm, cell junction	Protein localization
**61**-D3ZWF5	Transcription and mRNA export factor ENY2	**Eny2**	↓2.2	0.0038	Nucleus	RNA processing, protein transport
**62**-A0A0G2K904	RCG56631, isoform CRA_d	**Sltm**	↓2.2	0.0007	Nucleus	Regulation of transcription
**63**-P37805	Transgelin-3	**Tagln3**	↓2.2	0.0042	Nucleus, cytoplasm, cytoskeleton	Regulation of transcription
**64**-Q5XIW8	U4/U6.U5 tri-snRNP-associated protein 1	**Sart1**	↓2.2	0.0011	Nucleus	RNA processing
**65**-P69736	Endothelial differentiation-related factor 1	**Edf1**	↓2.2	0.0008	Cytoplasm, nucleus	Regulation of transcription, cell differentiation
**66**-Q5BJP2	Spliceosome-associated protein CWC15 homolog	**Cwc15**	↓2.2	0.0006	Nucleus	RNA processing
**67**-Q4QQU6	Survival of motor neuron-related-splicing factor 30	**Smndc1**	↓2.2	0.0478	Nucleus	RNA processing, apoptosis
**68**-Q5BJN8	Protein SDE2 homolog	**Sde2**	↓2.2	0.0268	Nucleus	Cell cycle, DNA processing
**69**-Q8R2E7	FAS-associated death domain protein	**Fadd**	↓2.1	0.0191	Cytoplasm, nucleus, cell membrane	Apoptosis, immunity
**70**-P30836	L-selectin	**Sell**	↓2.1	0.0082	Cell membrane	Cell adhesion
**71**-Q6TQE1	Zinc finger CCCH domain-containing protein 18	**Zc3h18**	↓2.1	0.0004	Nucleus	RNA processing
**72**-Q6MG48	Protein PRRC2A	**Prrc2a**	↓2.1	0.0006	Cytoplasm, nucleus	Cell differentiation
**73**-Q5I0E0	RNA methyltransferase	**Mepce**	↓2.1	0.0241	Nucleus	Regulation of transcription
**74**-Q5BJB3	Coiled-coil-helix-coiled-coil-helix domain containing 2	**Chchd2**	↓2.1	0.0032	Mitochondrion, nucleus	Oxidative stress response
**75**-G3V8C9	Nuclear receptor coactivator 6	**Ncoa6**	↓2.1	0.0197	Cytoplasm, nucleus	Regulation of transcription
**76**-B2RZ74	U1 small nuclear ribonucleoprotein 70 kDa	**Snrnp70**	↓2.1	0.0032	Cytoplasm, nucleus	RNA processing
**77**-P05964	Protein S100-A6	**S100a6**	↓2.1	0.0421	Cell membrane, cytoplasm, nucleus	Actin filament organization
**78**-P13668	Stathmin	**Stmn1**	↓2.1	0.0171	Cytoplasm, cytoskeleton	Signal transduction, neurogenesis
**79**-D4ACF1	Eukaryotic translation initiation factor 4E nuclear import factor 1	**Eif4enif1**	↓2.1	0.0012	Cytoplasm	Regulation of translation
**80**-Q498C9	RCG33491, isoform CRA_b	**Zfp207**	↓2.1	<0.0001	Cytoplasm, cytoskeleton, nucleus	Mitotic spindle assembly, protein stabilization
**81**-D4A913	Cofactor required for Sp1 transcriptional activation subunit 7	**Med26**	↓2.1	0.0369	Nucleus	Regulation of transcription
**82**-Q5BJK8	Golgi integral membrane protein 4	**Golim4**	↓2.1	0.0163	Endosome, GA, cell membrane	Protein transport
**83**-D3ZCL3	U1 small nuclear ribonucleoprotein C	**Snrpc**	↓2.1	<0.0001	Nucleus	RNA processing
**84**-B0BN48	Cyclin-dependent kinase 2-associated protein	**Cdk2ap1**	↓2.1	0.0437	Cytoplasm, nucleus	Regulation of protein phosphorylation
**85**-P49797	Regulator of G-protein signaling 3	**Rgs3**	↓2.1	0.0494	Cytoplasm, cell membrane, nucleus	Signal transduction
**86**-Q8K585	High mobility group protein HMG-I/HMG-Y	**Hmga1**	↓2.1	0.0151	Nucleus	Regulation of transcription
**87**-M0R6E6	Scaffold attachment factor B2	**Safb2**	↓2.0	0.0018	Nucleus	Regulation of transcription
**88**-D3ZC82	Nuclear FMR1-interacting protein 2	**Nufip2**	↓2.0	0.0027	Cytoplasm, nucleus	RNA processing
**89**-Q9JJT1	Glucocorticoid-attenuated response gene 16 product	**Ifit1**	↓2.0	0.0152	Cytoplasm	Response to interferons, response to virus
**90**-Q8VIL3	ZW10 interactor	**Zwint**	↓2.0	0.0019	Nucleus	Cell cycle, cell division

**91**-Q498U4	SAP domain-containing ribonucleoprotein	**Sarnp**	↓2.0	0.0029	Nucleus	RNA processing
**92**-A0A0G2K2M9	Serine/arginine repetitive matrix 2	**Srrm2**	↓2.0	0.0011	Nucleus	RNA processing
**93**-Q63014	A-kinase anchor protein 8	**Akap8**	↓2.0	0.0032	Cytoplasm, nucleus	Immunity, regulation of transcription, transport
**94**-Q63560	Microtubule-associated protein 6	**Map6**	↓2.0	0.0184	Cytoplasm, cytoskeleton, GA	Microtubule cytoskeleton organization

b) Subcellular localization and function of altered proteins isolated from **rat brain cortex** after 7-day treatment with **morphine**, **LYS739** and **LYS744** (***10 mg/kg***) identified by label-free quantification (MaxLFQ).

**Accession number**	**Protein name**	**Gene**	**Change (fold)**	**p value**	**Subcellular localization**	**Molecular functions and biological processes- keywords**
**CORTEX -MORPHINE - 10 mg/kg, 7 days**						
UP-regulated						
**1**-Q5XIP6	Flap endonuclease 1	**Fen1**	↑8.4	0.0032	Mitochondrion, nucleus	DNA processing
**2**-Q64349	Myelin and lymphocyte protein	**Mal**	↑5.0	0.0004	Cell membrane	Myelination, protein transport
**3**-D4AE49	Mtr4 exosome RNA helicase	**Mtrex**	↑3.2	0.0178	Nucleus	RNA processing
**4**-Q4FZS2	BUB3 mitotic checkpoint protein	**Bub3**	↑3.2	0.0044	Nucleus	Protein localization, chromosome segregation
**5**-Q75WE7	von Willebrand factor A domain-containing protein 5A	**Vwa5a**	↑3.0	0.0138	Nucleus	Tumor supressor
**6**-Q5M7V8	Thyroid hormone receptor-associated protein 3	**Thrap3**	↑2.5	0.0129	Nucleus	RNA processing
**7**-D4A648	Non-specific serine/threonine protein kinase	**Stk4**	↑2.4	0.0017	Cytoplasm, nucleus	Apoptosis, signal transduction
**8**-O35831	Cyclin-dependent kinase 17	**Cdk17**	↑2.2	0.0018	Cytoplasm, nucleus	Regulation of transcription, protein phosphorylation
**9**-P09495	Tropomyosin alpha-4 chain	**Tpm4**	↓2.1	0.0007	Cytoplasm, cytoskeleton	Actin filament organization
Down-regulated						
**1**-P50116	Protein S100-A9	**S100a9**	↓13.2	0.0002	Cell membrane, cytoplasm, secreted	Actin cytoskeleton organization, apoptosis, immunity
**2**-Q62733	Lamina-associated polypeptide 2, isoform beta	**Tmpo**	↓7.3	0.0002	Nucleus	Regulation of transcription
**3**-Q63014	A-kinase anchor protein 8	**Akap8**	↓7.2	0.0280	Cytoplasm, nucleus	Immunity, regulation of transcription, protein transport
**4**-B2RZ74	U1 small nuclear ribonucleoprotein 70 kDa	**Snrnp70**	↓7.1	0.0061	Cytoplasm, nucleus	RNA processing
**5**-Q6AYC4	Macrophage-capping protein	**Capg**	↓6.1	0.0127	Cytoplasm, nucleus	Actin polymerization/depolymerization
**6**-D3ZBN0	Histone H1.5	**Hist1h1b**	↓5.8	0.0037	Nucleus	DNA processing, nucleus organization

**7**-D3Z8V4	NCK associated protein 1 like (Predicted)	**Nckap1l**	↓4.4	0.0020	Cytoplasm	Cell homeostasis, apoptosis
**8**-P31000	Vimentin	**Vim**	↓4.0	0.0013	Cell membrane, cytoskeleton, nucleus	Aging, intermediate filament organization
**9**-P0C0S7	Histone H2A.Z	**H2az1**	↓3.3	0.0138	Nucleus	DNA processing
**10**-P18437	Non-histone chromosomal protein HMG-17	**Hmgn2**	↓3.2	<0.0001	Cytoplasm, nucleus	Regulation of transcription, chromation organization
**11**-B2RYB8	Integrin beta	**Itgb2**	↓3.1	0.0156	Cell membrane	Cell adhesion, aging
**12**-Q00729	Histone H2B type 1-A	**H2bc1**	↓2.9	0.0002	Nucleus	DNA processing
**13**-Q62780	Probable ATP-dependent RNA helicase DDX46	**Ddx46**	↓2.8	0.0185	Cell membrane, nucleus	RNA processing
**14**-P02262	Histone H2A type 1	***N/A***	↓2.4	<0.0001	Nucleus	DNA processing
**15**-O70377	Synaptosomal-associated protein 23	**Snap23**	↓2.4	0.0154	Cell membrane	Exocytosis
**16**-D3ZMR1	Mitochondrial import receptor subunit TOM7 homolog	**Tomm7**	↓2.3	0.0087	Mitochondrion	Protein transport
**17**-Q00566	Methyl-CpG-binding protein 2	**Mecp2**	↓2.2	0.0196	Nucleus	RNA processing
**18**-P62804	Histone H4	**H4c2**	↓2.2	<0.0001	Nucleus	DNA processing
**19**-Q04940	Neurogranin	**Nrgn**	↓2.1	0.0183	Cytoplasm	Brain development, synaptic plasticity
**20**-D3ZBE8	UPF3B, regulator of nonsense mediated mRNA decay	**Upf3b**	↓2.1	0.0158	Cytoplasm, nucleus	RNA processing
**21**-Q9JID1	Programmed cell death protein 4	**Pdcd4**	↓2.0	0.0015	Cytoplasm, nucleus	Apoptosis
**22**-P62329	Thymosin beta-4	**Tmsb4x**	↓2.0	0.0002	Cytoplasm, cytoskeleton	Actin cytoskeleton organization

CORTEX -LYS739 - 10 mg/kg, 7 days

UP-regulated

**1**-Q5XIP6	Flap endonuclease 1	**Fen1**	↑10.1	0.0108	Mitochondrion, nucleus	DNA processing
**2**-Q64349	Myelin and lymphocyte protein	**Mal**	↑9.5	0.0018	Cell membrane	Myelination, protein transport
**3**-Q75UQ2	Craniofacial development protein 1	**Cfdp1**	↑4.7	<0.0001	Nucleus	Brain development
4-Q7TQN4	Nuclear factor kappaB subunit p65	**Rela**	↑4.0	0.0096	Cytoplasm, nucleus	RNA processing, immunity, aging
**5**-Q75WE7	von Willebrand factor A domain-containing protein 5A	**Vwa5a**	↑3.9	0.0238	Nucleus	Tumor supressor
**6**-Q2LAP6	Testin	**Tes**	↑3.8	0.0103	Cytoplasm	Cell proliferation, cell adhesion
**7**-P09495	Tropomyosin alpha-4 chain	**Tpm4**	↑3.8	<0.0001	Cytoplasm, cytoskeleton	Actin filament organization
**8**-Q6XLI7	Copine-1-like	**Rbm12**	↑3.7	0.0285	Nucleus	RNA processing
**9**-Q4FZS2	BUB3 mitotic checkpoint protein	**Bub3**	↑3.7	0.0015	Nucleus	Protein localization, chromosome segregation
**10**-D4A5R0	Peptidyl-prolyl cis-trans isomerase	**Ppih**	↑3.6	0.0129	Cytoplasm, nucleus	RNA processing, protein folding
**11**-B5DFJ3	RNA helicase	**Ddx23**	↑3.6	0.0106	Nucleus	RNA processing
**12**-Q7M730	Sodium channel subunit beta-4	**Scn4b**	↑3.1	0.0008	Cell membrane	Ion transport
**13**-Q63610	Tropomyosin alpha-3 chain	**Tpm3**	↑3.1	<0.0001	Cytoplasm, cytoskeleton	Actin filament organization, brain development

**14**-Q9Z0T0	Thiopurine S-methyltransferase	**Tpmt**	↑3.0	0.0385	Cytoplasm	Xenobiotic metabolism, methylation
**15**-P09811	Glycogen phosphorylase, liver form	**Pygl**	↑3.0	0.0103	Cytoplasm	Glycogen metabolism
**16**-O35115	Four and a half LIM domains protein 2	**Fhl2**	↑2.9	0.0019	Cytoplasm, nucleus	RNA processing
**17**-Q6AZ25	Tropomyosin 1, alpha	**Tpm1**	↑2.9	<0.0001	Cytoplasm, cytoskeleton	Actin filament organization
**18**-Q4QQT3	CUGBP Elav-like family member 1	**Celf1**	↑2.8	0.0009	Cytoplasm, nucleus	RNA processing, brain development
**19**-O35831	Cyclin-dependent kinase 17	**Cdk17**	↑2.5	<0.0001	Cytoplasm, nucleus	Regulation of transcription, protein phosphorylation
**20**-P23593	Apolipoprotein D	**Apod**	↑2.4	0.0118	Secreted	Lipid transport, aging, brain development
**21**-P05504	ATP synthase subunit a	**Mt-Atp6**	↑2.3	0.0057	Mitochondrion	ATP synthesis, ion transport
**22**-P01048	T-kininogen 1	**Map1**	↑2.2	0.0459	Secreted	Protease inhibitor
**23**-Q63016	Large neutral amino acids transporter small subunit 1	**Slc7a5**	↑2.2	0.0020	Cell membrane, lysosome	Amino-acid transport
**24**-P10818	Cytochrome c oxidase subunit 6A1, mitochondrial	**Cox6a1**	↑2.2	0.0044	Mitochondrion	Respiratory electron transport
**25**-D3ZHK8	Cytoplasmic polyadenylation element-binding protein 2	**Cpeb2**	↑2.1	0.0276	Cytoplasm, nucleus	RNA processing, response to oxidative stress
**26**-F1M4A4	Kinesin-like protein KIF1A	**Kif1a**	↑2.1	0.0001	Cytoplasm, cytoskeleton, cell membrane	Anterograde axonal transport, motor protein
**27**-Q99JE4	Ras-specific guanine nucleotide-releasing factor 2	**Rasgrf2**	↑2.1	0.0058	Cell membrane, cytoplasm, ER	Signal transduction, response to ER stress
**28**-A0JN29	Endoplasmic reticulum junction formation protein lunapark	**Lnp**	↑2.1	0.0126	ER	ER organization
**29**-Q704E8	ATP-binding cassette sub-family B member 7, mitochondrial	**Abcb7**	↑2.0	0.0116	Mitochondrion	Transmembrane transport, iron ion homeostasis
**30**-P31423	Metabotropic glutamate receptor 4	**Grm4**	↑2.0	0.0015	Cell membrane	Signal transduction, apoptosis, learning
Down-regulated						
**1**-Q566E4	Heterogeneous nuclear ribonucleoprotein R	**Hnrnpr**	↓18.0	0.0001	Cytoplasm, nucleus	RNA processing
**2**-Q09167	Serine/arginine-rich splicing factor 5	**Srsf5**	↓17.4	0.0036	Nucleus	RNA processing
**3**-P50116	Protein S100-A9	**S100a9**	↓13.4	<0.0001	Cell membrane, cytoplasm, secreted	Actin cytoskeleton organization, apoptosis, immunity
**4**-Q8K5B5	Amino acid transporter	**Slc1a2**	↓13.3	0.0001	Cell membrane	Amino-acid transport
**5**-Q9JID1	Programmed cell death protein 4	**Pdcd4**	↓11.8	<0.0001	Cytoplasm, nucleus	Apoptosis
**6**-Q62771	Signal transducer and activator of transcription 5A	**Stat5a**	↓10.7	0.0029	Cytoplasm, nucleus	RNA processing, signal transduction
**7**-D3ZBN0	Histone H1.5	**Hist1h1b**	↓8.9	0.0045	Nucleus	DNA processing, nucleus organization
**8**-Q62733	Lamina-associated polypeptide 2, isoform beta	**Tmpo**	↓7.8	0.0002	Nucleus	Regulation of transcription
**9**-F7EY92	Methyl-CpG binding domain protein 3 (Predicted), isoform CRA_c	**Mbd3**	↓6.5	0.0067	Cytoplasm, nucleus	DNA processing, aging, brain development
**10**-D3ZUL1	Coiled-coil domain containing 124	**Ccdc124**	↓6.0	0.0088	Nucleus	Regulation of transcription
**11**-Q9JIL8	DNA repair protein RAD50	**Rad50**	↓5.7	0.0437	Nucleus	DNA processing, cell cycle
**12**-D3Z8V4	NCK associated protein 1 like (Predicted)	**Nckap1l**	↓5.6	0.0155	Cytoplasm	Cell homeostasis, apoptosis
**13**-A0A0G2K9C0	Vasodilator-stimulated phosphoprotein	**Vasp**	↓5.2	0.0300	Cytoplasm, cytoskeleton	Actin cytoskeleton organization
**14**-F1M981	Phosphatidylinositol 3,4,5-trisphosphate 5-phosphatase 1	**Inpp5d**	↓5.1	0.0154	Cytoplasm, cytoskeleton, cell membrane	Immunity, apoptosis, signal transduction

**15**-Q99P99	Histone deacetylase 4	**Hdac4**	↓5.1	0.0022	Cytoplasm, nucleus	Regulation of transcription, histone deacetylation
**16**-O55035	Peptidyl-prolyl cis-trans isomerase G	**Ppig**	↓5.1	0.0169	Nucleus	Protein folding
**17**-P18437	Non-histone chromosomal protein HMG-17	**Hmgn2**	↓5.1	<0.0001	Cytoplasm, nucleus	Regulation of transcription, chromation organization
**18**-Q00729	Histone H2B type 1-A	**H2bc1**	↓4.6	<0.0001	Nucleus	DNA processing
**19**-Q6MGA6	Proteasome subunit beta	**Psmb9**	↓4.4	0.0214	Cytoplasm, nucleus	Immunity, proteolysis
**20**-P02262	Histone H2A type 1	***N/A***	↓4.4	<0.0001	Nucleus	DNA processing
**21**-O08837	Cell division cycle 5-like protein	**Cdc5l**	↓4.2	0.0262	Cytoplasm, nucleus	Regulation of transcription, cell cycle
**22**-P0C0S7	Histone H2A.Z	**H2az1**	↓4.0	0.0471	Nucleus	DNA processing
**23**-P69736	Endothelial differentiation-related factor 1	**Edf1**	↓4.0	0.0036	Cytoplasm, nucleus	Regulation of transcription, cell differentiation
**24**-Q9JKS6	Protein piccolo	**Pclo**	↓3.6	<0.0001	Cell junction, synapse	Regulation of exocytosis
**25**-Q4KLG9	AN1-type zinc finger protein 2B	**Zfand2b**	↓3.6	0.0004	ER	Protein targeting to ER
**26**-F1LMV6	Desmoplakin	**Dsp**	↓3.6	0.0046	Cytoplasm, cytoskeleton	Desmosome organization, cell-cell adhesion
**27**-A0JPP1	Dr1-associated corepressor	**Drap1**	↓3.5	0.0068	Nucleus	Regulation of transcription
**28**-Q7TQ20	DnaJ homolog subfamily C member 2	**Dnajc2**	↓3.5	0.0003	Cytoplasm, nucleus	Regulation of transcription, chaperone
**29**-P62804	Histone H4	**H4c2**	↓3.5	<0.0001	Nucleus	DNA processing
**30**-F1MAF2	A-kinase-anchoring protein 17A	**Akap17a**	↓3.5	0.0175	Cytoplasm, nucleus	RNA processing
**31**-Q6U6G5	Zinc finger CCCH domain-containing protein 15	**Zc3h15**	↓3.4	0.0102	Cytoplasm, nucleus	Metal ion binding
**32**-Q5U2R6	Putative monooxygenase p33MONOX	**P33monox**	↓3.4	0.0002	Cytoplasm	Neuronal differentiation, oxidoreductase
**33**-Q1RP77	Nucleolar protein 16	**Nop16**	↓3.4	0.0016	Nucleus	Ribosomal large subunit biogenesis
**34**-B4F7A1	Complex III assembly factor LYRM7	**Lyrm7**	↓3.3	0.0005	Mitochondrion	Chaperone
**35**-O88751	Calcium-binding protein 1	**Cabp1**	↓3.3	0.0011	Cytoplasm, cytoskeleton	Developmental protein, visual perception
**36**-D4A9L2	Serine/arginine-rich splicing factor 1	**Srsf1**	↓3.2	0.0033	Cytoplasm, nucleus	RNA processing
**37**-O08838	Amphiphysin	**Amph**	↓3.1	0.0101	Cytoplasm, cytoskeleton, cell membrane	Endocytosis
**38**-D3ZGL1	Rho GTPase-activating protein 25	**Arhgap25**	↓3.1	0.0054	Cell membrane	Signal transduction
**39**-Q64LC9	RNA-binding protein 4B	**Rbm4**	↓3.0	0.0202	Nucleus	RNA processing
**40**-P62853	40S ribosomal protein S25	**Rps25**	↓2.9	0.0002	Cytoplasm, nucleus	RNA processing, ribonucleoprotein
**41**-P63090	Pleiotrophin	**Ptn**	↓2.9	<0.0001	Secreted	Growth factor, brain development
**42**-D4A4Z9	Kinectin 1	**Ktn1**	↓2.9	<0.0001	ER	Protein transport
**43**-B5DEK0	Regulation of nuclear pre-mRNA domain-containing 1B	**Rprd1b**	↓2.9	0.0103	Nucleus	RNA processing, cell cycle
**44**-A0A0G2JUA5	AHNAK nucleoprotein	**Ahnak**	↓2.8	<0.0001	Cytoplasm, nucleus, cell membrane	RNA processing
**45**-Q8R2H0	V-type proton ATPase subunit G	**Atp6v1g2**	↓2.8	0.0091	Cell membrane	Proton transmembrane transport
**46**-P84817	Mitochondrial fission 1 protein	**Fis1**	↓2.7	0.0057	Mitochondrion, peroxisome	Apoptosis, mitochondrial fusion
**47**-M0R7B4	H1.3 linker histone, cluster member	**Hist1h1d**	↓2.7	<0.0001	Nucleus	DNA processing, nucleus assembly
**48**-Q9EPJ0	Nuclear ubiquitous casein and cyclin-dependent kinase substrate 1	**Nucks1**	↓2.7	<0.0001	Nucleus	DNA processing

**49**-Q811U3	ELKS/Rab6-interacting/CAST family member 1	**Erc1**	↓2.7	0.0017	Cytoplasm, GA, cell membrane	Protein transport, retrograde transport
**50**-Q4QQV8	Charged multivesicular body protein 5	**Chmp5**	↓2.6	<0.0001	Cytoplasm, endosome, cell membrane	Protein transport, endosome to lysosome transport
**51**-Q66H20	Polypyrimidine tract-binding protein 2	**Ptbp2**	↓2.6	0.0075	Nucleus	RNA processing
**52**-P60841	Alpha-endosulfine	**Ensa**	↓2.6	<0.0001	Cytoplasm	Cell cycle
**53**-B0BN72	Protein FAM195B	**Mcrip1**	↓2.6	0.0005	Cytoplasm, nucleus	Regulation of epithelial to mesenchymal transition
**54**-P04646	60S ribosomal protein L35a	**Rpl35a**	↓2.6	0.0036	Cytoplasm	Cytoplasmic translation
**55**-F1LQH2	Nuclear factor NF-kappa-B p105 subunit	**Nfkb1**	↓2.6	0.0217	Cytoplasm, nucleus	Regulation of transcription
**56**-B2RZ79	Iron-sulfur cluster assembly enzyme	**Iscu**	↓2.6	0.0006	Cytoplasm, mitochondrion	Iron ion homeostasis
**57**-Q6AYC4	Macrophage-capping protein	**Capg**	↓2.6	0.0394	Cytoplasm, nucleus	Actin polymerization/depolymerization
**58**-D3ZPG5	Ubiquitin carboxyl-terminal hydrolase 30	**Usp30**	↓2.6	0.0009	Mitochondrion	Protein ubiquitination
**59**-D3ZC82	Nuclear FMR1-interacting protein 2	**Nufip2**	↓2.5	<0.0001	Cytoplasm, nucleus	RNA processing
**60**-P21818	Stathmin-2	**Stmn2**	↓2.5	0.0004	Cytoplasm, endosome, GA	Regulator of microtubule stability
**61**-A0A0G2JZZ4	Selenoprotein M	**Selenom**	↓2.5	0.0008	ER, GA	Hormone metabolic process, tissue development
**62**-D4A2D3	RCR-type E3 ubiquitin transferase	**Mycbp2**	↓2.5	0.0114	Cell projection, cytoplasm	Protein ubiquitination
**63**-P34926	Microtubule-associated protein 1A	**Map1a**	↓2.4	<0.0001	Cytoplasm, cytoskeleton	Microtubule cytoskeleton organization
**64**-Q03344	ATPase inhibitor, mitochondrial	**Atpif1**	↓2.4	0.0003	Mitochondrion	Regulation of ATP metabolic process
**65**-Q99068	Alpha-2-macroglobulin receptor-associated protein	**Lrpap1**	↓2.4	<0.0001	GA, ER, endosome	Endocytosis, signal transduction
**66**-P27321	Calpastatin	**Cast**	↓2.4	<0.0001	Cytoplasm, nucleus	Protease inhibitor, aging, brain development
**67**-P11517	Hemoglobin subunit beta-2	***N/A***	↓2.4	<0.0001	Cytoplasm	Oxygen transport
**68**-Q91XQ4	DNA-directed RNA polymerase II subunit GRINL1A	**Polr2m**	↓2.4	0.0044	Nucleus	Regulation of transcription, signal transduction
**69**-Q8VIL3	ZW10 interactor	**Zwint**	↓2.4	<0.0001	Nucleus	Cell cycle, cell division
**70**-Q925G0	RNA-binding protein 3	**Rbm3**	↓2.4	0.0037	Cytoplasm, nucleus	RNA processing, stress response
**71**-D4A1B8	Dynactin subunit 3	**Dctn3**	↓2.3	0.0075	Cytoplasm, cytoskeleton	Cytoskeleton-dependent cytokinesis
**72**-Q157S1	CREB-regulated transcription coactivator 1	**Crtc1**	↓2.3	<0.0001	Cytoplasm, nucleus	Regulation of transcription, energy homeostasis
**73**-Q9Z0W5	Protein kinase C and casein kinase substrate in neurons protein 1	**Pascin1**	↓2.3	<0.0001	Cell membrane, cytoplasm	Actin filament organization, endocytosis
**74**-P61959	Small ubiquitin-related modifier 2	**Sumo2**	↓2.3	0.0090	Nucleus	Protein ubiquitination, regulation of transcription
**75**-Q27W01	RNA-binding protein 8A	**Rbm8a**	↓2.3	0.0051	Cytoplasm, nucleus	RNA processing
**76**-P83565	39S ribosomal protein L40, mitochondrial	**Mrpl40**	↓2.3	0.0039	Mitochondrion	Regulation of translation, ribonucleoprotein
**77**-B0K008	Eukaryotic translation initiation factor 1	**Eif1**	↓2.3	0.0074	Cytoplasm, nucleus	Regulation of translation
**78**-P07171	Calbindin	**Calb1**	↓2.3	0.0185	Cytoplasm, cytoskeleton, nucleus	Vitamin D metabolism, learning, memory
**79**-O35543	Hematopoietic prostaglandin D synthase	**Hpgds**	↓2.3	0.0434	Cytoplasm	Lipid metabolism, prostaglandin metabolism
**80**-Q63364	E3 ubiquitin-protein ligase Praja-2	**Pja2**	↓2.3	0.0316	Cytoplasm, cell membrane, ER, GA	Immunity, protein ubiquitination
**81**-Q5XII0	Mammalian ependymin-related protein 1	**Epdr1**	↓2.3	0.0061	Lysosome, secreted	Cell-matrix adhesion
**82**-Q00566	Methyl-CpG-binding protein 2	**Mecp2**	↓2.3	0.0191	Nucleus	RNA processing

**83**-Q8K1Q4	Leucine zipper putative tumor suppressor 3	**Lzts3**	↓2.3	<0.0001	Cytoplasm, cytoskeleton, cell junction	Regulation of dendritic spine morphogenesis
**84**-Q62785	28 kDa heat- and acid-stable phosphoprotein	**Pdap1**	↓2.3	<0.0001	Cytoplasm	Signal transduction
**85**-B2RYW7	Signal recognition particle 14 kDa protein	**Srp14**	↓2.2	0.0007	Cytoplasm	Protein targeting to ER
**86**-D3ZQL7	Tubulin polymerization-promoting protein	**Tppp**	↓2.2	0.0090	Cytoplasm, cytoskeleton, GA, nucleus	Microtubule cytoskeleton organization, cell cycle
**87**-Q5RKG9	Eukaryotic translation initiation factor 4B	**Eif4b**	↓2.2	<0.0001	Cytoplasm	Translation initiation factor activity
**88**-P04937	Fibronectin	**Fn1**	↓2.2	0.0160	Secreted	Cell adhesion, cell shape
**89**-P63055	Purkinje cell protein 4	**Pcp4**	↓2.2	<0.0001	Cytoplasm, cytoskeleton	Signal transduction, apoptosis
**90**-F1MA98	Nucleoprotein TPR	**Tpr**	↓2.2	<0.0001	Nucleus, cytoplasm, cell membrane	Cell cycle, mRNA transport
**91**-Q5PQM2	Kinesin light chain 4	**Klc4**	↓2.2	0.0226	Cytoplasm, cytoskeleton	Motor protein
**92**-P63100	Calcineurin subunit B type 1	**Ppp3r1**	↓2.2	0.0007	Cell membrane, cytoplasm	Protein import into nucleus, calcium ion binding
**93**-B5DF60	Eukaryotic translation initiation factor 4C	**Eif1ax**	↓2.2	0.0017	Cytoplasm	Regulation of translation
**94**-Q712U5	cAMP-regulated phosphoprotein 19	**Arpp19**	↓2.2	<0.0001	Cytoplasm	Cell cycle, cell division
**95**-Q5BJB3	Coiled-coil-helix-coiled-coil-helix domain containing 2	**Chchd2**	↓2.2	0.0003	Mitochondrion, nucleus	Oxidative stress response, mitochondrion organization
**96**-Q5I034	Protein CUSTOS	**Custos**	↓2.1	<0.0001	Nucleus	Developmental protein
**97**-D4A9Z8	Chromatin-modifying protein 4B-like 1	**Chmp4bl1**	↓2.1	<0.0001	Endosome, cell membrane	Late endosome to vacuole transport
**98**-Q9EPJ3	28S ribosomal protein S26, mitochondrial	**Mrps26**	↓2.1	0.0008	Mitochondrion	Regulation of translation
**99**-A0A0G2KAS8	Non-specific serine/threonine protein kinase	**Wnk2**	↓2.1	0.0224	Cytoplasm	Signal transduction, ion homeostasis
**100**-Q5XI72	Eukaryotic translation initiation factor 4H	**Eif4h**	↓2.1	<0.0001	Cytoplasm	Regulation of translation
**101**-Q62839	Golgin subfamily A member 2	**Golga2**	↓2.1	0.0004	GA, cytoplasm, cytoskeleton	ER to Golgi vesicle-mediated transport, cell cycle
**102**-B2RZA9	Ube 2l3 protein	**Ube2l3**	↓2.1	<0.0001	Cytoplasm, nucles	Protein ubiquitination, nucleus
**103**-Q498U4	SAP domain-containing ribonucleoprotein	**Sarnp**	↓2.1	0.0462	Nucleus	RNA processing
**104**-Q810U0	Coiled-coil domain-containing protein 50	**Ccdc50**	↓2.1	0.0068	Cytoplasm, cytoskeleton	Sensory perception of sound
**105**-M0R919	Prefoldin subunit 3	**Vbp1**	↓2.1	0.0015	Cytoplasm	Protein folding
**106**-Q8VHK2	Caskin-1	**Caskin1**	↓2.1	<0.0001	Cytoplasm	Signal transduction
**107**-Q99MC0	Protein phosphatase 1 regulatory subunit 14A	**Ppp1r14a**	↓2.1	0.0387	Cytoplasm	Protein phosphatase inhibitor
**108**-Q9JHZ4	GRIP1-associated protein 1	**Gripap1**	↓2.1	0.0001	Endosome, cell junction	Protein transport
**109**-Q5PPN5	Tubulin polymerization-promoting protein family member 3	**Tppp3**	↓2.1	<0.0001	Cytoplasm, cytoskeleton	Microtubule cytoskeleton organization
**110**-P28494	Alpha-mannosidase 2	**Man2a1**	↓2.1	0.0364	GA	Mannose metabolism
**111**-A0A0G2JTW9	Hemoglobin, beta adult s chain	**Hbb-b1**	↓2.1	0.0005	Cytoplasm, secreted	Oxygen transport
**112**-F1LNK0	Microtubule-associated protein	**Map2**	↓2.1	<0.0001	Cytoplasm, cytoskeleton	Microtubule cytoskeleton organization
**113**-D3ZC15	Capping protein regulator and myosin 1 linker 2	**Carmil2**	↓2.1	0.0024	Cytoplasm	Actin filament network formation
**114**-Q499N6	UBX domain-containing protein 1	**Ubxn1**	↓2.1	<0.0001	Cytoplasm	Protein ubiquitination
**115**-Q63042	FAD-linked sulfhydryl oxidase ALR	**Gfer**	↓2.1	0.0098	Mitochondrion	Growth factor, apoptosis
**116**-D4AE79	Charged multivesicular body protein 1a	**Chmp1a**	↓2.1	<0.0001	Cytoplasm, endosome, cell membrane	Endosome transport, protein transport

**117**-D3ZXK3	Transforming, acidic coiled-coil-containing protein 2	**Tacc2**	↓2.0	0.0021	Cytoplasm, cytoskeleton	Microtubule cytoskeleton organization
**118**-P0C5H9	Mesencephalic astrocyte-derived neurotrophic factor	**Manf**	↓2.0	<0.0001	ER, secreted	Growth factor, neuron projection development
**119**-Q792I0	Protein lin-7 homolog C	**Lin7c**	↓2.0	0.0031	Cell membrane, cell junction	Exocytosis
**120**-Q9Z1W6	Protein LYRIC	**Mtdh**	↓2.0	<0.0001	Cell membrane, cytoplasm, ER, nucleus	Regulation of transcription, apoptosis
**121**-O35179	Endophilin-A1	**Sh3gl2**	↓2.0	0.0430	Cytoplasm, cell membrane, endosome	Endocytosis
**122**-D4A7N1	MICOS complex subunit Mic25	**Chchd6**	↓2.0	0.0018	Mitochondrion	Cristae formation

CORTEX -LYS744 - 10 mg/kg, 7 days

UP-regulated

**1**-Q64349	Myelin and lymphocyte protein	**Mal**	↑5.7	0.0011	Cell membrane	Myelination, protein transport
**2**-Q4FZS2	BUB3 mitotic checkpoint protein	**Bub3**	↑4.0	0.0004	Nucleus	Protein localization, chromosome segregation
**3**-Q9JIL8	DNA repair protein RAD50	**Rad50**	↑4.0	0.0038	Nucleus	DNA processing, cell cycle
**4**-Q6AXS3	Protein DEK	**Dek**	↑3.5	0.0031	Nucleus	Chromatin organization
**5**-P05504	ATP synthase subunit a	**Mt-Atp6**	↑3.1	0.0057	Mitochondrion	ATP synthesis, ion transport
**6**-Q4QQT3	CUGBP Elav-like family member 1	**Celf1**	↑3.0	0.0055	Cytoplasm, nucleus	RNA processing, brain development

Down-regulated

**1**-P50116	Protein S100-A9	**S100a9**	↓5.4	0.0064	Cell membrane, cytoplasm, secreted	Actin cytoskeleton organization, apoptosis, immunity
**2**-D3Z8V4	NCK associated protein 1 like (Predicted)	**Nckap1l**	↓5.2	0.0005	Cytoplasm	Cell homeostasis, apoptosis
**3**-Q62780	Probable ATP-dependent RNA helicase DDX46	**Ddx46**	↓4.2	0.0248	Cell membrane, nucleus	RNA processing
**4**-Q7TQ20	DnaJ homolog subfamily C member 2	**Dnajc2**	↓4.1	0.0122	Cytoplasm, nucleus	Regulation of transcription, chaperone
**5**-Q6AYC4	Macrophage-capping protein	**Capg**	↓3.6	0.0090	Cytoplasm, nucleus, melanosome	Actin polymerization/depolymerization
**6**-Q00729	Histone H2B type 1-A	**H2bc1**	↓3.6	0.0026	Nucleus	DNA processing
**7**-D4A9D6	DEAH box protein 9	**Dhx9**	↓3.2	0.0268	Nucleus	Helicase, regulation of transcription
**8**-P18437	Non-histone chromosomal protein HMG-17	**Hmgn2**	↓3.1	<0.0001	Cytoplasm, nucleus	Regulation of transcription, chromation organization
**9**-P31000	Vimentin	**Vim**	↓3.0	0.0172	Cell membrane, cytoskeleton, nucleus	Aging, intermediate filament organization
**10**-P49134	Integrin beta-1	**Itgb1**	↓2.7	0.0199	Endosome, cell membrane, cell junction	Cell adhesion, receptor internalization
**11**-Q5XIK2	Thioredoxin-related transmembrane protein 2	**Tmx2**	↓2.7	0.0008	Mitochondrion, ER	Brain development
**12**-P02262	Histone H2A type 1	***N/A***	↓2.5	<0.0001	Nucleus	DNA processing
**13**-M0R7B4	H1.3 linker histone, cluster member	**Hist1h1d**	↓2.5	<0.0001	Nucleus	DNA processing, nucleus assembly
**14**-Q641Z4	Cyclin-dependent kinase 9	**Cdk9**	↓2.5	0.0090	Cytoplasm, nucleus	Regulation of transcription

**15**-Q00566	Methyl-CpG-binding protein 2	**Mecp2**	↓2.2	0.0128	Nucleus	RNA processing
**16**-P62804	Histone H4	**H4c2**	↓2.1	<0.0001	Nucleus	DNA processing
**17**-Q01827	Synaptic vesicular amine transporter	**Slc18a2**	↓2.1	0.0021	Cell membrane	Neurotransmitter transport, aging
**18**-A0A0G2JZZ4	Selenoprotein M	**Selenom**	↓2.1	0.0002	ER, GA	Hormone metabolic process, tissue development
**19**-Q6EV70	GDP-fucose protein O-fucosyltransferase 1	**Pofut1**	↓2.1	0.0057	ER	Carbohydrate metabolism
**20**-P19527	Neurofilament light polypeptide	**Nefl**	↓2.0	0.0001	Cytoplasm, cytoskeleton	Microtubule cytoskeleton organization, brain development

c) Subcellular localization and function of altered proteins isolated from **rat hippocampus** after 7-day treatment with **morphine**, **LYS739** and **LYS744** (***10 mg/kg***) identified by label-free quantification (MaxLFQ).

**Accession number**	**Protein name**	**Gene**	**Change (fold)**	**p value**	**Subcellular localization**	**Molecular functions and biological processes- keywords**
**HIPPOCAMPUS -MORPHINE - 10 mg/kg, 7 days**						
UP-regulated						
**1**-F1LMV6	Desmoplakin	**Dsp**	↑7.0	0.0131	Cytoplasm, cytoskeleton	Desmosome organization, cell-cell adhesion
**2**-Q63688	25-hydroxycholesterol 7-alpha-hydroxylase	**Cyp7b1**	↑3.8	0.0034	ER	Cholesterol metabolism
**3**-Q6P756	Adaptin ear-binding coat-associated protein 2	**Necap2**	↑3.3	0.0228	Cell membrane	Endocytosis, protein transport
**4**-B5DFJ3	RNA helicase	**Ddx23**	↑2.4	0.0263	Nucleus	RNA processing
**5**-Q6P0K8	Junction plakoglobin	**Jup**	↑2.3	<0.0001	Cytoplasm, cytoskeleton, cell junction	Cell-cell adhesion, desmosome assembly
**6**-Q6J1Y9	Ubiquitin carboxyl-terminal hydrolase 19	**Usp19**	↑2.2	0.0011	ER	Protein ubiquitination
**7**-D4A997	HIV TAT specific factor 1 (Predicted)	**Htatsf1**	↑2.1	0.0201	Nucleus	RNA processing
**8**-D3ZP06	Integrin beta	**Itgb8**	↑2.1	0.0094	Cell membrane	Cell adhesion, immunity
Down-regulated						
**1**-B2RZ74	U1 small nuclear ribonucleoprotein 70 kDa	**Snrnp70**	↓10.8	0.0005	Cytoplasm, nucleus	RNA processing
**2**-Q63862	Myosin-11	**Myh11**	↓5.9	<0.0001	Cytoplasm, melanosome	Cytoskeletal motor activity
**3**-Q64349	Myelin and lymphocyte protein	**Mal**	↓3.8	0.0401	Cell membrane	Myelination, protein transport
**4**-F7FF45	Nuclear mitotic apparatus protein 1	**Numa1**	↓3.7	0.0330	Cytoplasm, cytoskeleton, GA, nucleus	Microtubule bundle formation
**5**-Q6PEC1	Tubulin-specific chaperone A	**Tbca**	↓3.7	<0.0001	Cytoplasm, cytoskeleton	Protein folding

**6**-P18437	Non-histone chromosomal protein HMG-17	**Hmgn2**	↓3.5	<0.0001	Cytoplasm, nucleus	Regulation of transcription, chromation organization
**7**-G3V752	RNA cytidine acetyltransferase	**Nat10**	↓3.5	0.0003	Nucleus	RNA processing
**8**-D3ZWF5	Transcription and mRNA export factor ENY2	**Eny2**	↓3.2	0.0015	Nucleus	RNA processing, protein transport
**9**-Q5FVI4	Cell cycle exit and neuronal differentiation protein 1	**Cend1**	↓3.0	<0.0001	Cell membrane	Neuronal differentiation
**10**-P18588	Interferon-induced GTP-binding protein Mx1	**Mx1**	↓2.9	0.0243	Cytoplasm, nucleus, ER	Immunity
**11**-P31232	Transgelin	**Tagln**	↓2.8	<0.0001	Cytoplasm	Actin filament binding
**12**-O35179	Endophilin-A1	**Sh3gl2**	↓2.8	0.0073	Cytoplasm, cell membrane, endosome	Endocytosis
**13**-D4ABK7	Heterogeneous nuclear ribonucleoprotein H3	**Hnrnph3**	↓2.7	0.0127	Nucleus	RNA processing
**14**-B2RYS2	Cytochrome b-c1 complex subunit 7	**Uqcrb**	↓2.6	<0.0001	Mitochondrion	Electron transport chain
**15**-B2RZ79	Iron-sulfur cluster assembly enzyme	**Iscu**	↓2.6	0.0008	Cytoplasm, mitochondrion	Iron ion homeostasis
**16**-P37361	Metallothionein-3	**Mt3**	↓2.5	0.0166	Cytoplasm, mitochondrion, nucleus, ER	Ion homeostasis, brain development, oxidative stress
**17**-E9PST5	Apoptotic chromatin condensation inducer 1	**Acin1**	↓2.5	0.0255	Cytoplasm, nucleus, cell membrane	Apoptosis
**18**-P60841	Alpha-endosulfine	**Ensa**	↓2.5	<0.0001	Cytoplasm	Cell cycle
**19**-Q5U2U8	BAG cochaperone 3	**Bag3**	↓2.4	0.0165	Cytoplasm, nucleus, cell membrane	Chaperone, brain development
**20**-P02650	Apolipoprotein E	**Apoe**	↓2.3	0.0048	Secreted	Lipid transport, lipid metabolism, aging
**21**-P11030	Acyl-CoA-binding protein	**Dbi**	↓2.3	0.0035	ER, GA	Acyl-CoA metabolism, brain development
**22**-P11517	Hemoglobin subunit beta-2	***N/A***	↓2.3	<0.0001	Cytoplasm	Oxygen transport
**23**-Q03344	ATPase inhibitor, mitochondrial	**Atpif1**	↓2.3	0.0002	Mitochondrion	Regulation of ATP metabolic process
**24**-Q6MGC4	H2-K region express gene 2, rat orthologue	**Pfdn6**	↓2.2	0.0003	Cytoplasm	Protein folding
**25**-P63219	Guanine nucleotide-binding protein G(I)/G(S)/G(O) subunit gamma-5	**Gng5**	↓2.2	0.0018	Cell membrane	Signal transduction
**26**-Q5PQN0	Neurocalcin-delta	**Ncald**	↓2.2	0.0004	Cytoplasm	Calcium-mediated signaling
**27**-Q5PPN7	Mitochondrial potassium channel	**Ccdc51**	↓2.2	0.0032	Mitochondrion	Ion transport
**28**-P63255	Cysteine-rich protein 1	**Crip1**	↓2.2	0.0002	Cytoplasm	Immunity, zinc ion binding
**29**-P21818	Stathmin-2	**Stmn2**	↓2.1	<0.0001	Cytoplasm, endosome, GA	Regulator of microtubule stability
**30**-D3ZME7	HscB mitochondrial iron-sulfur cluster co-chaperone	**Hscb**	↓2.1	0.0005	Mitochondrion, cytoplasm	Chaperone
**31**-O08837	Cell division cycle 5-like protein	**Cdc5l**	↓2.1	0.0038	Cytoplasm, nucleus	Regulation of transcription, cell cycle
**32**-D4A4T0	RING-type E3 ubiquitin transferase	**Stub1**	↓2.0	0.0007	Cytoplasm, ER, nucleus	Chaperone, protein ubiquitination
**33**-P62076	Mitochondrial import inner membrane translocase subunit Tim13	**Timm13**	↓2.0	0.0003	Mitochondrion	Chaperone
**34**-Q4KLG9	AN1-type zinc finger protein 2B	**Zfand2b**	↓2.0	<0.0001	ER	Protein targeting to ER

HIPPOCAMPUS -LYS739 - 10 mg/kg, 7 days

UP-regulated

**1**-P04177	Tyrosine 3-monooxygenase	**Th**	↑10.0	0.0002	Cytoplasm, cell projection	Catecholamine biosynthesis, brain development
**2**-D4AE49	Mtr4 exosome RNA helicase	**Mtrex**	↑6.0	0.0151	Nucleus	RNA processing
**3**-F1LMV6	Desmoplakin	**Dsp**	↑4.2	0.0368	Cytoplasm, cytoskeleton	Desmosome organization, cell-cell adhesion
**4**-P54100	Proto-oncogene vav	**Vav1**	↑4.2	0.0406	Cell-cell junction	Immunity, cell development
**5**-Q5XIK2	Thioredoxin-related transmembrane protein 2	**Tmx2**	↑3.9	0.0345	Mitochondrion, ER	Brain development
**6**-A0A0G2QC38	Serine and arginine-rich-splicing factor 11	**Srsf11**	↑3.1	0.0155	Nucleus	RNA processing
**7**-P19527	Neurofilament light polypeptide	**Nefl**	↑2.9	<0.0001	Cytoplasm, cytoskeleton	Microtubule cytoskeleton organization, brain development
**8**-P09495	Tropomyosin alpha-4 chain	**Tpm4**	↑2.8	0.0001	Cytoplasm, cytoskeleton	Actin filament organization
**9**-Q63327	Myelin-associated oligodendrocyte basic protein	**Mobp**	↑2.7	0.0008	Cytoplasm	CNS myelin formation, brain development
**10**-P20417	Tyrosine-protein phosphatase non-receptor type 1	**Ptpn1**	↑2.7	0.0245	ER, cell membrane	Signal transduction, endocytosis
**11**-Q9QUH6	Ras/Rap GTPase-activating protein SynGAP	**Syngap1**	↑2.6	0.0011	Cell membrane, cell junction	Signal transduction, brain development
**12**-Q63610	Tropomyosin alpha-3 chain	**Tpm3**	↑2.6	<0.0001	Cytoplasm, cytoskeleton	Actin filament organization, brain development
**13**-P23565	Alpha-internexin	**Ina**	↑2.3	0.0007	Cytoplasm, cytoskeleton	Cytoskeleton organization, developmental protein
**14**-Q99P82	Claudin-11	**Cldn11**	↑2.3	0.0005	Cell membrane	Cell adhesion
**15**-D3ZP06	Integrin beta	**Itgb8**	↑2.2	0.0079	Cell membrane	Cell adhesion, immunity
**16**-P12839	Neurofilament medium polypeptide	**Nefm**	↑2.2	<0.0001	Cytoplasm, cytoskeleton	Neurofilament cytoskeleton organization
**17**-Q68FX4	Hematopoietic cell-specific LYN substrate 1	**Hcls1**	↑2.1	0.0324	Cytoplasm, nucleus, cell membrane	Regulation of actin filament polymerization
**18**-P37377	Alpha-synuclein	**Snca**	↑2.1	0.0272	Cytoplasm, cell membrane, nucleus	Regulation of neurotransmitter release, endocytosis
**19**-D3ZFK5	Microsomal signal peptidase 12 kDa subunit	**Spcs1**	↑2.1	0.0004	ER, cell membrane	Protein targeting to ER
**20**-P48550	G protein-activated inward rectifier potassium channel 2	**Kcnj6**	↑2.0	0.0158	Cell membrane	Ion transport
Down-regulated						
**1**-F7FF45	Nuclear mitotic apparatus protein 1	**Numa1**	↓11.7	0.0017	Cytoplasm, cytoskeleton, GA, nucleus	Microtubule bundle formation
**2**-Q63862	Myosin-11	**Myh11**	↓10.1	<0.0001	Cytoplasm, melanosome	Cytoskeletal motor activity
**3**-P31000	Vimentin	**Vim**	↓7.2	0.0037	Cell membrane, cytoskeleton, nucleus	Aging, intermediate filament organization
**4**-P31232	Transgelin	**Tagln**	↓7.1	<0.0001	Cytoplasm	Actin filament binding
**5**-F1LM60	ArfGAP with RhoGAP domain, ankyrin repeat and PH domain 1	**Arap1**	↓6.2	0.0002	Cytoplasm	Signal transduction
**6**-Q64349	Myelin and lymphocyte protein	**Mal**	↓4.3	0.0293	Cell membrane	Myelination, protein transport
**7**-G3V790	Transcription activator BRG1	**Smarca4**	↓3.7	0.0010	Nucleus	Regulation of transcription
**8**-A0A0G2JUA5	AHNAK nucleoprotein	**Ahnak**	↓3.7	<0.0001	Cytoplasm, nucleus, cell membrane	RNA processing
**9**-E9PST5	Apoptotic chromatin condensation inducer 1	**Acin1**	↓3.5	0.0084	Cytoplasm, nucleus, cell membrane	Apoptosis
**10**-O08837	Cell division cycle 5-like protein	**Cdc5l**	↓3.5	0.0070	Cytoplasm, nucleus	Regulation of transcription, cell cycle
**11**-P18437	Non-histone chromosomal protein HMG-17	**Hmgn2**	↓3.4	0.0050	Cytoplasm, nucleus	Regulation of transcription, chromation organization

**12**-P63255	Cysteine-rich protein 1	**Crip1**	↓3.3	0.0085	Cytoplasm	Immunity, zinc ion binding
**13**-G3V752	RNA cytidine acetyltransferase	**Nat10**	↓3.3	<0.0001	Nucleus	RNA processing
**14**-Q6J2U6	E3 ubiquitin-protein ligase RNF114	**Rnf114**	↓3.0	0.0319	Cytoplasm, nucleus	Protein ubiquitination, developmental protein
**15**-C0JPT7	Filamin A	**Flna**	↓2.7	0.0008	Cell membrane, cytoplasm, nucleus, GA	Actin cytoskeleton organization
**16**-D3ZWF5	Transcription and mRNA export factor ENY2	**Eny2**	↓2.6	0.0005	Nucleus	RNA processing, protein transport
**17**-D4A720	RCG61762, isoform CRA_d	**Srsf7**	↓2.5	0.0136	Nucleus	RNA processing
**18**-P11517	Hemoglobin subunit beta-2	***N/A***	↓2.3	<0.0001	Cytoplasm	Oxygen transport
**19**-D3ZBE8	UPF3B, regulator of nonsense mediated mRNA decay	**Upf3b**	↓2.1	0.0002	Cytoplasm, nucleus	RNA processing
**20**-Q62736	Non-muscle caldesmon	**Cald1**	↓2.1	0.0016	Cytoplasm, cytoskeleton	Actin filament bundle assembly
**21**-D4A4Z9	Kinectin 1	**Ktn1**	↓2.1	<0.0001	ER	Protein transport
**22**-Q6MGC4	H2-K region express gene 2, rat orthologue	**Pfdn6**	↓2.0	0.0003	Cytoplasm	Protein folding
**23**-F1LNK0	Microtubule-associated protein	**Map2**	↓2.0	<0.0001	Cytoplasm, cytoskeleton	Microtubule cytoskeleton organization
**24**-Q8R2H0	V-type proton ATPase subunit G	**Atp6v1g2**	↓2.0	0.0012	Cell membrane	Proton transmembrane transport
**25**-P01355	Cholecystokinin	**Cck**	↓2.0	0.0003	Secreted	Hormone activity, neuron migration

HIPPOCAMPUS -LYS744 - 10 mg/kg, 7 days

UP-regulated

**1**-Q5XIK2	Thioredoxin-related transmembrane protein 2	**Tmx2**	↑3.9	0.0346	Mitochondrion, ER	Brain development
**2**-Q63688	25-hydroxycholesterol 7-alpha-hydroxylase	**Cyp7b1**	↑3.1	0.0039	ER	Cholesterol metabolism
**3**-A0A0G2QC38	Serine and arginine-rich-splicing factor 11	**Srsf11**	↑3.0	0.0158	Nucleus	RNA processing
**4**-Q9JHU0	Dihydropyrimidinase-related protein 5	**Dpysl5**	↑2.8	<0.0001	Cytoplasm	Neuron differentiation
**5**-F1LVV4	Regulator of chromosome condensation 2	**Rcc2**	↑2.2	0.0087	Cytoplasm, nucleus, endosome	Protein localization, cell cycle
**6**-A0A0G2JXD9	DOP1 leucine zipper-like protein B	**Dop1b**	↑2.0	0.0011	Cytoplasm, endosome, GA	Protein transport

Down-regulated

**1**-Q63862	Myosin-11	**Myh11**	↓15.0	0.0001	Cytoplasm, melanosome	Cytoskeletal motor activity
**2**-Q64349	Myelin and lymphocyte protein	**Mal**	↓12.3	0.0159	Cell membrane	Myelination, protein transport
**3**-P31000	Vimentin	**Vim**	↓10.4	0.0001	Cell membrane, cytoskeleton, nucleus	Aging, intermediate filament organization
**4**-B2RZ74	U1 small nuclear ribonucleoprotein 70 kDa	**Snrnp70**	↓6.5	0.0120	Cytoplasm, nucleus	RNA processing
**5**-P31232	Transgelin	**Tagln**	↓4.6	<0.0001	Cytoplasm	Actin filament binding
**6**-Q6J2U6	E3 ubiquitin-protein ligase RNF114	**Rnf114**	↓3.8	0.0261	Cytoplasm, nucleus	Protein ubiquitination, developmental protein

**7**-D3ZMS1	Splicing factor 3b, subunit 2	**Sf3b2**	↓3.6	0.0102	Nucleus	RNA processing
**8**-F1M0V4	THO complex subunit 2	**Thoc2**	↓3.2	0.0274	Nucleus	RNA processing
**9**-D4ABK7	Heterogeneous nuclear ribonucleoprotein H3	**Hnrnph3**	↓3.2	0.0211	Nucleus	RNA processing
**10**-A0A0G2JUA5	AHNAK nucleoprotein	**Ahnak**	↓2.8	<0.0001	Cytoplasm, nucleus, cell membrane	RNA processing
**11**-C0JPT7	Filamin A	**Flna**	↓2.6	0.0007	Cell membrane, cytoplasm, nucleus, GA	Actin cytoskeleton organization
**12**-D4A720	RCG61762, isoform CRA_d	**Srsf7**	↓2.6	0.0111	Nucleus	RNA processing
**13**-P07150	Annexin A1	**Anxa1**	↓2.6	0.0002	Cell membrane, cytoplasm, endosome	Immunity, signal transduction
**14**-P62804	Histone H4	**H4c2**	↓2.5	<0.0001	Nucleus	DNA processing
**15**-Q07936	Annexin A2	**Anxa2**	↓2.4	0.0016	Melanosome, secreted	Endocytosis, membrane raft assembly
**16**-P63255	Cysteine-rich protein 1	**Crip1**	↓2.4	0.0002	Cytoplasm	Immunity, zinc ion binding
**17**-Q62736	Non-muscle caldesmon	**Cald1**	↓2.3	0.0001	Cytoplasm, cytoskeleton	Actin filament bundle assembly
**18**-Q5XIW8	U4/U6.U5 tri-snRNP-associated protein 1	**Sart1**	↓2.2	0.0340	Nucleus	RNA processing
**19**-P08010	Glutathione S-transferase Mu 2	**Gstm2**	↓2.1	0.0012	Cytoplasm	Lipid metabolism, aging
**20**-P02262	Histone H2A type 1	***N/A***	↓2.0	0.0006	Nucleus	DNA processing

*GA – Golgi apparatus

**ER – Endoplasmic reticulum
